# Supplementary material for: Auditing the quality of epidemic decision-making in Somalia: a pilot evaluation
Source: BMJ Open. 2023 Jan 3;13(1):e065122. doi: 10.1136/bmjopen-2022-065122 (PMC9815027; doi:10.1136/bmjopen-2022-065122)
Supplement: Supplementary data [file bmjopen-2022-065122supp001.pdf]

Evaluating the quality of epidemic decision-making in epidemics & humanitarian crises

Standard Operating Procedures (SOP)

Version 1.0
June 30th, 2021

Abdihamid Warsame
Infectious Disease Epidemiology
London School of Hygiene & Tropical Medicine
Abdihamid.warsame@lshtm.ac.uk

Contents

Evaluating epidemic decision-making in humanitarian crises 1
Standard Operating Procedures (SOP) 1
Abbreviations 4
Introduction 5
Rationale 6
Definitions 6
Target audience 6
Evaluation team composition 6
Timeline 7
Section 1- Context analysis 8
1.1 Purpose 8
1.2 Roles and responsibilities 8
1.3 Procedures 8
1.4 Outputs 8
1.5 Associated Documents 8
Section 2- Decision Selection 8
2.1 Purpose 9
2.2 Roles and Responsibilities 9
2.3 Procedures 9
2.4 Outputs 9
2.5 Associated Documents 9
Section 3- Decision Scoring 10
3.1 Purpose 10
3.2 Roles and responsibilities 10
3.3 Procedures 10
3.4 Outputs 10
3.5 Associated Documents 10
Section 4- Dissemination/ Review 11
4.1 Purpose 11
4.2 Roles and responsibilities 11
4.3 Procedures 11
4.4 Outputs 11
4.5 Associated Documents 11
Reference 12

Annex 13
Document Checklist 13
Decision-making evaluation tool .... **Error! Bookmark not defined.**
Background .... **Error! Bookmark not defined.**
Part A- Context analysis .... **Error! Bookmark not defined.**
Part B- Selection of critical decision points .... **Error! Bookmark not defined.**
Key informant interview guide .... **Error! Bookmark not defined.**
Flow chart .... **Error! Bookmark not defined.**

Warsame A, *et al. BMJ Open* 2023; 13:e065122. doi: 10.1136/bmjopen-2022-065122

Abbreviations

CO- Country Office

COVID-19- Coronavirus disease 2019

RO- Regional Office

HQ- headquarters

Sitrep- Situation report

PHSA- Public Health Situation Analysis

RRA- Rapid risk assessment

SMT- Senior Management Team

KII- Key Informant Interview

FGD- Focus Group Discussion

Warsame A, *et al. BMJ Open* 2023; 13:e065122. doi: 10.1136/bmjopen-2022-065122

Introduction

While epidemics of infectious disease continue to pose a considerable threat to populations in low-income and humanitarian settings, the evaluation of these epidemic responses are limited and heterogeneous [1]. Populations in need of humanitarian assistance or living within low income settings continue to grow[2] and the ongoing COVID-19 pandemic has further exacerbated the impact of these crises[3]. Decision-making in the context of an ongoing epidemic is particularly challenging[13,14]. Previous research has identified a limited number of decision-making factors contributing to poor response [15–17]. However, these findings need further study in order to confirm their relevance to a wider array of contexts experiencing ongoing epidemics

Such settings not only must deal with the epidemic directly and its indirect impacts but must also contend with a host of urgent competing emergencies. As such decision-makers are under heavier strain than those in better resourced and stable environments and require adequate support. COVID-19 is unlikely to be the last epidemic faced by decision-makers in such settings and therefore evaluations such as this are especially critical to strengthening future preparedness and improving response.

In decision-making research, there are two common approaches: process and evaluation. Process oriented approach describes how decisions are actually made within a specific context whereas analytical oriented is more prescriptive and frames decision-making as a problem-solving approach intended to lead to the best outcome. In short the former looks at whether a decision is made correctly while the latter considers whether the correct decision is made. This evaluation is predicated on the former approach.

Rationale

- This SOP is intended to evaluate the process of epidemic decision-making rather than the impact of any specific decision. Improved decision-making practises can improve outcomes, timeliness and accountability in epidemic response in humanitarian settings.

- In order to contribute to better humanitarian performance, the UKRI awarded a consortium led by the London School of Hygiene & Tropical Medicine to undertake research and produce evidence to support humanitarian responders. This SOP includes stepwise actions required to be undertaken by evaluators as well as the necessary timeframes, roles and responsibilities and resources required.

Definitions

Decision making: choosing between number of options/course of action or a process of problem solving in a specific context (Campbell 2018)

Evaluation- assessment, as systematic and impartial as possible of an activity, project, programme, strategy, policy, theme, sector, operational area or institutional performance.

Target audience

- This standard operating procedure is intended for the evaluation lead within the humanitarian organization. However, it can also be utilized by third party monitors as part of an overarching review of a humanitarian organizations epidemic response.

Evaluation team composition

Although evaluation should ideally be done by independent third party, staff members in the evaluated organization have a significant role to play in the planning, design, implementation and use of this evaluation.

Timeline

- The evaluation should ideally take place after the first X weeks since the onset of the epidemic. It may also take place immediately after the conclusion of the epidemic

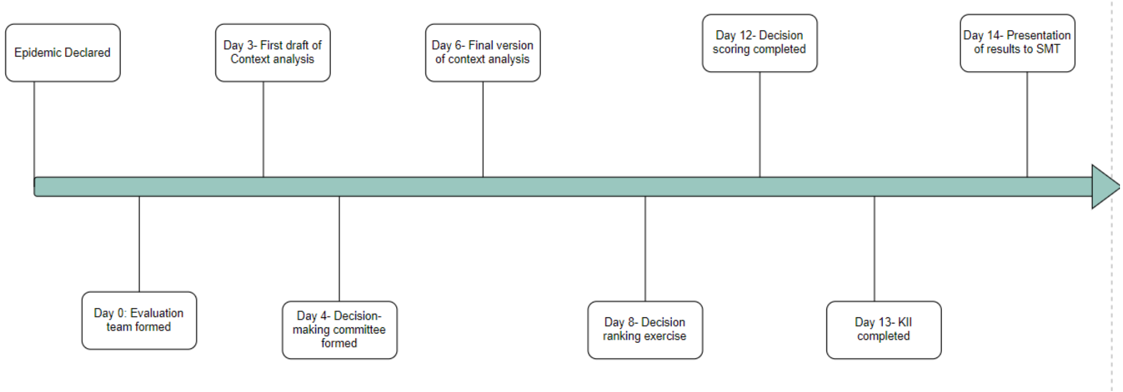

Section 1- Context analysis

1.1 Purpose

- The purpose of the context analysis is to provide a common base from which to understand the epidemic setting and the actions that have been taken.

1.2 Roles and responsibilities

- The evaluation team lead will lead the exercise and convene the evaluation team to undertake the context analysis.
- The evaluation team will identify principle decision-makers to form decision-making committee

1.3 Procedures

- The evaluation team will undertake a secondary data review in order to produce the first draft of the context analysis
- The review should gather all relevant documentation including:
  - Previous Public Health Situation Analyses (PHSA)
  - Rapid risk assessments (RRA)
  - Response planning documentation
  - Organizational organograms
  - Sitreps
  - Health bulletins
  - Communication products
  - Meeting minutes
- The evaluation team will also identify key internal decision-makers involved in the particular epidemic response who have had significant input in the planning, implementation or evaluation of the epidemic response. These maybe situated within the country office or regional or HQ levels. These may include technical experts, organizational leaders and others
- These decision-makers will be invited to form a committee to contribute to the remaining sections of this evaluation
- The evaluation team lead will circulate the context analysis draft and solicit feedback in a timely manner from the decision-makers
- The evaluation team will consolidate context analysis feedback into a final version

1.4 Outputs

- Consolidated and agreed upon context analysis which summarizes epidemiological description of the epidemic, health resources and services available to the population, health system performance, organizational resources expended in the epidemic response to date, as well as challenges faced in the response

1.5 Associated Documents

See Annex for document checklist, participant list and section 1 of the tool

Section 2- Decision Selection

2.1 Purpose

The purpose of this section is to produce a unified list of key decisions to evaluate as prelude to evaluate decision-making in the epidemic response.

2.2 Roles and Responsibilities

- The evaluation team lead is responsible for sharing key documentation and producing an initial listing of key decisions
- The decision-making committee is responsible for reviewing, contributing and evaluating the criticality of decisions

2.3 Procedures

- The evaluation team lead will circulate the context analysis and introduce the characteristics for selecting key decisions to the evaluation team.
- In conjunction with the evaluation team, he/she will produce up to 5 key decisions for initial consideration by key decision-making committee identified through the context analysis
- The key decision-makers will review the proposed decisions
- Decision-makers may contribute further decisions for consideration
- Decision-makers will score each of the proposed decisions based on the scoring criteria
- Evaluation team will summarize (take the mean) the score for each decision and select the top 5 decisions using the following formula

2.4 Outputs

- Unified list of no more than 5 key decisions to be further assessed in section 3.

2.5 Associated Documents

See annex for section 2 of the tool

Warsame A, *et al. BMJ Open* 2023; 13:e065122. doi: 10.1136/bmjopen-2022-065122

Section 3- Decision Scoring

3.1 Purpose

The purpose of this section is to ensure that selected critical decisions are evaluated consistently across the assessment criteria and to produce a summary score for the overall epidemic decision-making.

3.2 Roles and responsibilities

- The evaluation team lead is responsible for explaining the evaluation dimensions and criteria
- The evaluation team is responsible for assessing each decision on the criteria

3.3 Procedures

- For each of the selected decisions (maximum 5) the evaluation team will assess whether the necessary decision-making criteria has been met
- They will compile evidence through review of documentation and score the decision on a scale of 1 (no evidence/not met) to 5 (criteria fully met)
- Where there is little or no evidence of a criteria being met, the evaluation team may revert to decision-making committee to provide evidence
- The decision-making committee members are responsible for providing evidence for any specific criteria as requested by the evaluation team
- The evaluation team lead will review the final score for each decision as well compute an overall score for the exercise

3.4 Outputs

- An agreed upon set of scores for each of the critical decisions as well as an overall score

3.5 Associated Documents

Refer to annex for section3 of the tool

Warsame A, *et al. BMJ Open* 2023; 13:e065122. doi: 10.1136/bmjopen-2022-065122

Section 4- Dissemination/ Review

4.1 Purpose

- The purpose of this section is to review and disseminate the findings of the evaluation in order to improve response processes

4.2 Roles and responsibilities

- The evaluation team lead is responsible for compiling the views of organizational members on the overall evaluation process
- The evaluation team lead is responsible for presenting the findings of the evaluation back to the organization
- The organizational leadership is responsible for working with relevant staff members in implementing improvements

4.3 Procedures

- The evaluation team lead should convene staff who have participated in the evaluation and implement focus group discussion (FGD) or key informant interviews(KII) to elicit feedback on the process
- The evaluation team should present the results of both the exercise and participant views to organizational leadership/ Senior Management Team (SMT)in a brief
- The SMT should implement recommendations derived from the exercise

4.4 Outputs

- Identification of decision rated as strong
- Recommendations of decision making dimensions that require strengthening
- Feedback on the utility and feasibility of evaluation
- Identification of areas of the evaluation process that require improvement

4.5 Associated Documents

- See annex for focus group guide and key informant interview guide

Reference

https://jhumanitarianaction.springeropen.com/articles/10.1186/s41018-020-00068-2#Fn3
https://www.who.int/hac/techguidance/tools/standard_operating_procedures_african_region_en_2014.pdf

https://healthcluster.who.int/docs/librariesprovider16/meeting-reports/updated-phsa-long-form-template-english-june-2019.docx?sfvrsn=81cd6a71_3

Warsame A, *et al. BMJ Open* 2023; 13:e065122. doi: 10.1136/bmjopen-2022-065122

Annex

Document Checklist

No.	Document Type	Details (e.g.)
1	Organogram	*CO organogram March 2021*
2	Meeting Minutes	*Weekly minutes (may 2021-June 2021)*
3		
4		
5		

Evaluation Participants

Evaluation Team	Name (e.g.)	Role
1	*John Doe*	*Team lead*
2	*Pers Indi*	*M&E officer*
3		
Decision-making committee	Name	Role
1	*Fulan*	*Incident manager*
2	*Jama Yulo*	*Operations officer*
3	*Qofa Kale*	*Technical officer*

Warsame A, *et al. BMJ Open* 2023; 13:e065122. doi: 10.1136/bmjopen-2022-065122
