# Supplementary material for: Auditing the quality of epidemic decision-making in Somalia: a pilot evaluation
Source: BMJ Open. 2023 Jan 3;13(1):e065122. doi: 10.1136/bmjopen-2022-065122 (PMC9815027; doi:10.1136/bmjopen-2022-065122)
Supplement: Supplementary data [file bmjopen-2022-065122supp003.pdf]

Part B- Selection of critical decision points

Guide

Considering the following guiding Characteristics, propose 3 -5 critical decision in the response to date. Justify your selection with specific with reference to the characteristics as well as specific examples. A decision does not need to meet all of the following characteristics to be considered critical. These can include decision on beginning, ending or scaling a response, deciding on response options, deciding on target population, as well as deciding on modality of response.

Characteristics of critical decision

I. Consequential- critical decision is consequential and shapes the response to a significant degree
II. Reversibility- a critical decision can be difficult to overturn or reverse at least in the short term)
III. Strategic- entails shift in terms of action taken, resources committed or precedent set
IV. Uncertainty –Wide range of uncertainty/ complex array of options
V. Reputionally Risky- Entails a high level of organisational reputational risk

Decision 1

Description
Describe the decision

Rationale
Justify your proposed decision by selecting the relevant Characteristics
☐ Characteristic 1

☐ Characteristic 2

☐ Characteristic 3

☐ Characteristic 4

☐ Characteristic 5

Narrative
Describe any other reason for considering this decision critical (maximum 200 words)

Decision 2

Description
Describe the decision

Rationale
Justify your proposed decision by selecting the relevant Characteristics

☐ Characteristic 1

☐ Characteristic 2

☐ Characteristic 3

☐ Characteristic 4

☐ Characteristic 5

Narrative
Describe any other reason for considering this decision critical (maximum 200 words)

Decision 3

Description
Describe the decision

Rationale
Justify your proposed decision by selecting the relevant Characteristics

☐ Characteristic 1

☐ Characteristic 2

☐ Characteristic 3

☐ Characteristic 4

☐ Characteristic 5

Narrative
Describe any other reason for considering this decision critical (maximum 200 words)

Warsame A, *et al. BMJ Open* 2023; 13:e065122. doi: 10.1136/bmjopen-2022-065122

LONDON SCHOOL of HYGIENE &TROPICAL MEDICINE

3
