# Supplementary material for: Auditing the quality of epidemic decision-making in Somalia: a pilot evaluation
Source: BMJ Open. 2023 Jan 3;13(1):e065122. doi: 10.1136/bmjopen-2022-065122 (PMC9815027; doi:10.1136/bmjopen-2022-065122)
Supplement: Supplementary data [file bmjopen-2022-065122supp004.pdf]

LONDON SCHOOL of HYGIENE &TROPICAL MEDICINE

Part C-Decision-making criteria

For each selected decision in Part B, complete the following scoring exercise, keeping in mind to provide the necessary evidence.

Decision: __*e.g. Decision to conduct mobile vaccinations in region Y in May 2020*

Transparency

To what degree were the following criteria met?

Criteria	Fulfilment of criteria *To what extent was the criteria fulfilled in this particular decision*	Evidence of fulfilment *Provide reference to specific sources to support your assessment*
Inclusivity[1]	5- Very Strong evidence	*e.g. Refer to meeting minutes March 24, 2020 as well as response plan April 2020. These show presence of diverse stakeholders in this decision from all departments as well as MoH*
Usage of explicit decision-making criteria[2]	Choose an item.	
Following clear process or method[3]	Choose an item.	
Usage of mechanism to publicise rationale [4]	Choose an item.	

Guide

1. Consider who was involved. Was there heterogeneity in ranks and roles?

2. Were the goals and objectives clearly pre-specified or were decisions rationalized after the fact?

Warsame A, et al. BMJ Open 2023; 13:e065122. doi: 10.1136/bmjopen-2022-065122

3. Were there documented priority setting process and/or use of priority setting framework? Was there a decision-tree

4. Was there clear documentation on decisions reached?

For each selected decision in Part B, complete the following scoring exercise, keeping in mind to provide the necessary evidence.

Decision: ____

Contestability

To what degree was the following criteria met?

Criteria	Fulfilment of criteria<br>*To what extent was the criteria fulfilled in this particular decision*	Evidence of fulfilment<br>*Provide reference to specific sources to support your assessment*
Opportunity for revision[5]	Choose an item.	
Was the decision devolved?[6]	Choose an item.	

Accountability

To what degree was the following criteria met?

Criteria	Fulfilment of criteria<br>*To what extent was the criteria fulfilled in this particular decision*	Evidence of fulfilment<br>*Provide reference to specific sources to support your assessment*
Engagement with effected communities[7]	Choose an item.	

Rigour

Criteria	Fulfilment of criteria *To what extent was the criteria fulfilled in this particular decision*	Evidence of fulfilment *Provide reference to specific sources to support your assessment*
Explicit outcome[8]	Choose an item.	
Feasible outcome[9]	Choose an item.	
Strengthen healthcare system[10]	Choose an item.	
Evidence based [11]	Choose an item.	

Guide

5. Was there any scope to revise and overturn a decision? How open was the discussion? Were alternatives debated? What were they? Was consensus achieved and if so, how was this reached?

6. What role did respondents in closest proximity to the epidemic (e.g. subnational level) or local technical experts play? What role did organizational leadership play? Who was the most junior decision-maker? Who was the most senior?

7. To what degree were the effected communities involved in the response? Who was involved from the effected community? Were they informed of the response activities? Did this notice make any difference for those notified?

8. Were intended outcomes of the decision clearly articulated? Were targets set? What were they?

9. Were alternatives debated? What were they?

10. Was there evidence that this decision was in-line with wider strategy? What pillar of the health system were targeted?

LONDON SCHOOL of HYGIENE &TROPICAL MEDICINE

11. Was “Strong public health rationale and solid scientific information provided to justify health measures?” What evidence was utilized? What evidence was missing?

Part D- Summary Decision-making Score

Scoring formula

For each assessed decision, utilize the formulae below to populate the table

Transparency score = average of criteria 1-4

Contestability= average of criteria 5-6

Accountability= score of criteria 7

Rigour= average of criteria 8-11

**Dimension**	**Average**	**Score**
Transparency		
Contestability		
Rigour		
Accountability		

Warsame A, *et al. BMJ Open* 2023; 13:e065122. doi: 10.1136/bmjopen-2022-065122

LONDON SCHOOL *of* HYGIENE &TROPICAL MEDICINE

Recommendations

Based on the results above, briefly describe what specific steps you recommend in order to improve the decision-making process in this epidemic *(maximum 300 words).*

Warsame A, *et al. BMJ Open* 2023; 13:e065122. doi: 10.1136/bmjopen-2022-065122

LONDON SCHOOL *of* HYGIENE &TROPICAL MEDICINE

LONDON SCHOOL of HYGIENE &TROPICAL MEDICINE
