# Supplementary material for: Auditing the quality of epidemic decision-making in Somalia: a pilot evaluation
Source: BMJ Open. 2023 Jan 3;13(1):e065122. doi: 10.1136/bmjopen-2022-065122 (PMC9815027; doi:10.1136/bmjopen-2022-065122)
Supplement: Supplementary data [file bmjopen-2022-065122supp005.pdf]

LONDON SCHOOL of HYGIENE &TROPICAL MEDICINE

Decision-making Scorecard

WHO Somalia's COVID-19 response

Evaluation study

Nov 22nd- December 8th 2021

Abdihamid Warsame

Faculty of Epidemiology and Population health

London School of Hygiene and Tropical Medicine

Critical Decisions

Participants were advised to select important decisions for critical evaluation based on the following criteria on what constitutes a critical decision.

Characteristics

A critical decision is one which has one or more of the following characteristics:

I. Consequentiality-: A critical decision is consequential and shapes the response to a significant degree
II. Reversibility-: A critical decision can be difficult to overturn or reverse at least in the short term
III. Strategic: A critical decision entails a significant shift in terms of action taken, resources committed or precedent set
IV. Uncertainty: A critical decision entails a wide range of uncertainty or complex array of options
V. Reputational Risk: A critical decision entails a high level of organisational reputational risk

Decision	Criteria for Selection
i. Formation of Rapid Response Teams	☒ Characteristic 1<br>☐ Characteristic 2<br>☒ Characteristic 3<br>☒ Characteristic 4<br>☒ Characteristic 5
ii. Establishment of 3 strategic PCR labs	☒ Characteristic 1<br>☒ Characteristic 2<br>☒ Characteristic 3<br>☒ Characteristic 4<br>☒ Characteristic 5
iii. Launch of Incident Management System	☒ Characteristic 1<br>☐ Characteristic 2<br>☒ Characteristic 3<br>☒ Characteristic 4<br>☒ Characteristic 5
iv. Focus on case management through scale up of Oxygen	☒ Characteristic 1<br>☐ Characteristic 2<br>☒ Characteristic 3<br>☒ Characteristic 4<br>☒ Characteristic 5

Summary Decision Scorecard

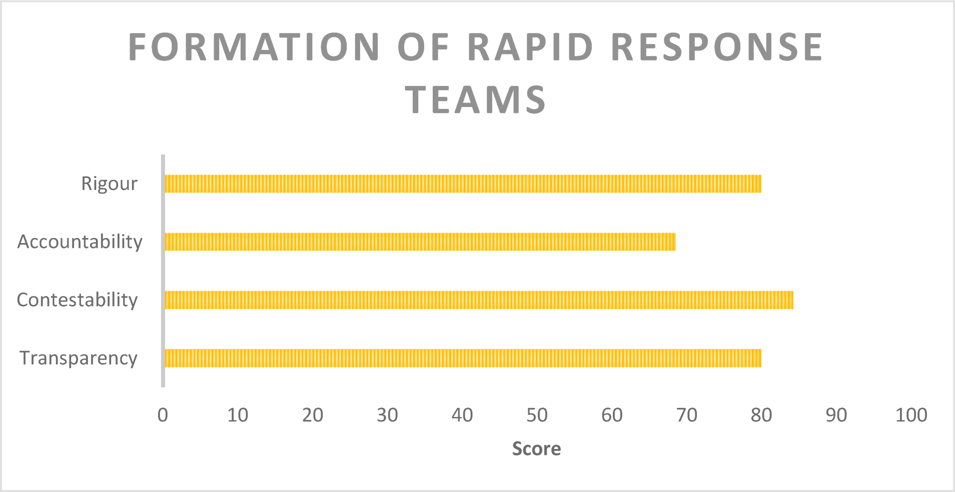

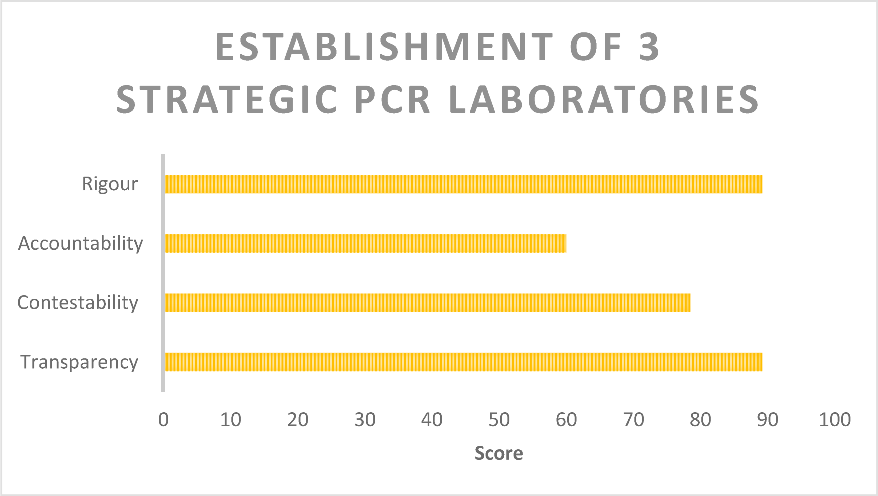

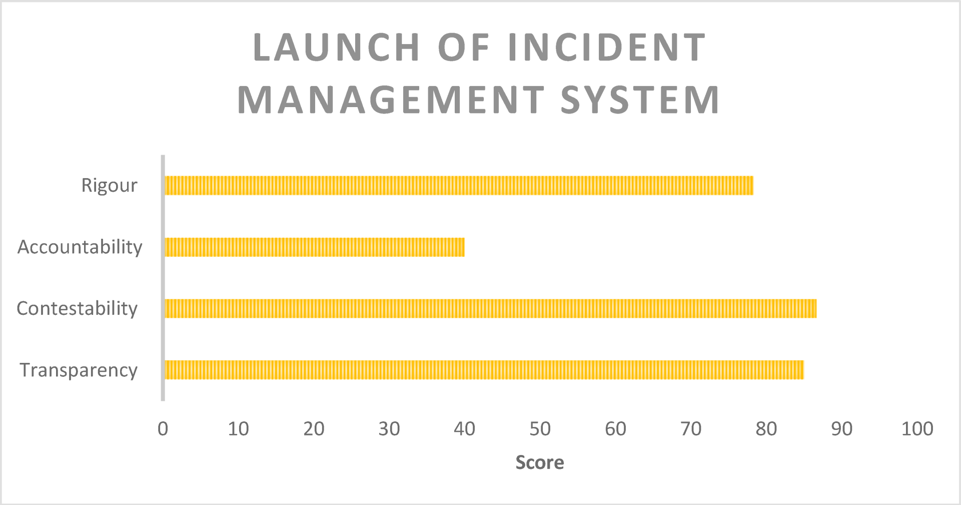

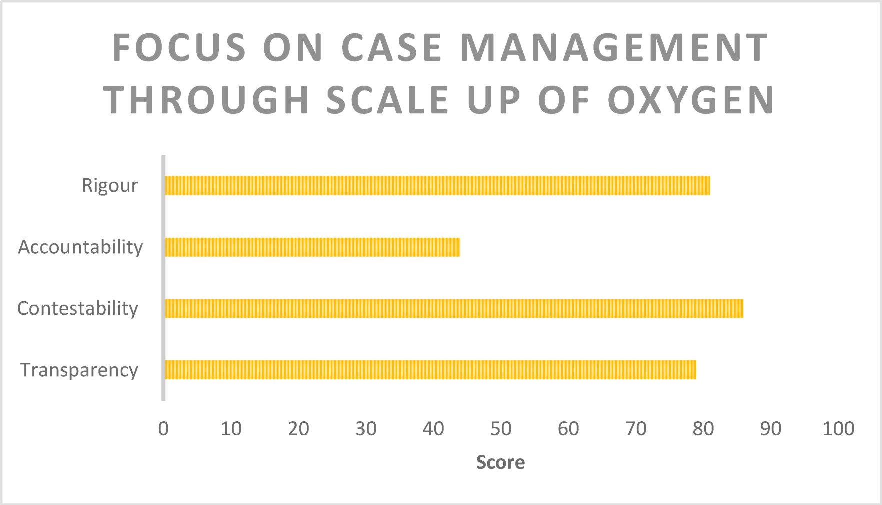

Detailed Scores & Interpretation

i. Formation of Rapid Response Teams

The Rapid Response Teams (RRT) and Community Health Workers (CHW) were recruited early in the COVID-19 response in order to strengthen and expand the surveillance capacity in high priority districts. A decision was undertaken to train and equip members of the RRTs and CHWs for case detection, notification, testing and contact tracing.

Inclusivity 86%	Decision criteria 74%	Process 77%	Public rationale 83%

Participants scored the transparency of this decision as high citing existing documentation such as response plan, IMST meeting minutes and proposal documents. They stated that there was strong evidence of inclusive decision-making as the decision included WHO staff from all programs as well as program managers. They also stated that some of the decision-makers were assigned to liaise/coordinate with the stakeholders including MoH and other partners and discuss the decision with them and publicise the rationale. They also noted following clear method for arriving at this decision including utilizing risk assessments. Participants referenced the usage of COVID-19 transmission classification for Somalia drawn from the interim guidelines by WHO for considerations for implementing and adjusting public health and social measures in the context of COVID-19 as providing clear criteria for this decision.

Devolution 74%	Revision 94%	Community engagement 69%	Explicit outcome 77%

Contestability was the highest scored dimension. Participants noted there were sufficient opportunities to revise the decision and cited the inter-action review of November 2020 as evidence. Additionally, the decision was discussed in various IMST meeting with document shared and commented, feedbacks incorporated into the final decision. The MoH was also said to be involved in the selection of RRTs and CHWs, implementation and modification of the structure and role and responsibilities. They also noted that revision of this decision resulted in expansion of the RRT mechanism to further district. Participants also noted that the IMST structure allowed for devolution in the decision-making in which decision-making was shared amongst senior decision-makers (country level IMST team members under the leadership of the WR) and junior decision makers (state level and district level staff)

In terms of accountability, this was scored lower as there was difference of scoring amongst participants as to the degree of engagement with the effected communities. Some participants stated that there was strong engagement with communities in implementing the RRT strategy but limited engagement in the decision to use this mechanism.

Feasibility 88%	Strengthen Health system 74%	Evidence based 77%

Participants noted this decision was taken with a high degree of rigour. In particular, they noted that strengthening healthcare system was among the objectives. Furthermore, they noted that the outcomes of this decision were explicitly articulated and include bridging the gap between health facilities and communities. They also noted that feasibility criteria were considered to be high due to staffing the RRT via the existing network of CHWs. Lastly they noted that targets were set and tracked through an interactive dashboard.

ii. Establishment of 3 strategic PCR labs

At the outset of the pandemic in Somalia, there did not exist national capacity to test samples for COVID-19. Responders relied in the first weeks, upon sending samples for testing abroad which were found to be time consuming and expensive. The lack of national labs to undertake this critical function was found to be a gap which required urgent attention. As a result, the decision was taken to establish three strategic PCR capable laboratories in Mogadishu, Garowe and Hargeisa.

Inclusivity 91%	Decision criteria 89%	Process 89%	Public rationale 89%

The transparency score is comprised of 4 criteria. In terms of inclusivity, participants scored this decision highly. terms of transparency. The decision was taken in conjunction with the MoH and UN partners. The rationale behind this decision was said to have been shared in internal coordination meetings as well as external coordination meetings with partners. A number of documents were cited as evidence including WHO Somalia Technical Program Update Quarterly, IMST meeting minutes, CERF Project Action Plan and WCO FCDO report.

Devolution 65%	Revision 91%	Community engagement 60%	Explicit outcome 91%

In terms of contestability, this decision was reviewed in the initial proposal development meeting with the MoH in which there was opportunity for revision. Additionally, participants stated that a review of the decision prompted further procurement of PCR machines and scaling up of PCR testing capacity to remaining states.

Participants expressed mixed scoring on the accountability dimension. Some stated that there was high accountability through interaction with state level MoH while others expressed that there was limited/no consultation with communities in these 3 cities regarding the establishment of PCR capacity in these labs. Some participants stated this criterion was inapplicable as the labs were pre-existing and upgrading did not require further engagement.

Feasibility 91%	Strengthen Health system 89%	Evidence based 89%

Participants scored this decision highly in terms of scientific rigour. They stated that the usage of PCR is global standard to detect COVID-19. Additionally, they stated the decision was built on evidence extracted from a previous 2019 influenza testing lab assessment which provided rationale and process for the decision to set up these labs for COVID-19 testing. Participants also cited the National Action Plan for Health Security as providing evidence for this decision. The decision was also found to have satisfied the strengthened the health system criteria as the establishment of these 3 labs allowed for testing beyond COVID-19.

iii. Launch of Incident Management System

As part of efforts to enhance the effectiveness of the Health Emergencies response and in line with the Emergency response framework, WHO Somalia launched the Incident Management Support Team to coordinate and guide the response to the COVID-19 pandemic within Somalia.

Inclusivity 90%	Decision criteria 83%	Process 80%	Public rationale 87%

Participants scored the inclusivity of this decision as high as they stated this decision was taken after a series of consultation with staff led by the WR. These deliberations were captured in meeting minutes and which detailed the process and rationale. The final decision was communicated by WR to all staff through an office memo. Participants also noted the engagement with the MoH in the formation of the IMST system as evidence of inclusivity.

Devolution 80%	Revision 93%	Community engagement 40%	Explicit outcome 77%

In terms of revision, participants noted that there was some opportunity which resulted in the change of incident managers but not in the structure itself. With regards to accountability, participants noted limited to no engagement with effected communities. However other participants stated that parallel state and district-level IMST did engage with the local community. While these lower level IMST did not feed into the formation of the national IMST, they maintain a certain level autonomy.

Feasibility 83%	Strengthen Health system 80%	Evidence based 73%

Participants cited a number of routine information products as evidence of decision outcomes and feasibility considerations. They cited COVID-19 Response plan and action trackers as evidence for the articulation of clear outcomes and evidence based targets. They also cited this response plan which articulated heath system strengthening as an explicit goal and an important pillar in the IMST approach. Participants also cited the WHO Global guidance on strategic response to C19 as providing evidence to support this decision.

iv. Focus on case management through scale up of Oxygen

Due to the presentation of symptoms and the aetiology of the disease as well as gaps in case management capacity, WHO Somalia undertook concerted effort to scale up oxygen therapy across the country in order to improve outcomes.

Inclusivity 76%	Decision criteria 76%	Process 76%	Public rationale 88%

The decision-making was scored as somewhat inclusive by participants. They cited the IMST meeting minutes in June 2020 as evidence as well as the COVID-19 response plan which detailed the discussion and decision criteria. They noted close deliberation with the case management focal persons within the MoH. Participants also noted that the rationale was communicated clearly through specific information products and web stories on WHO and other websites.

Devolution 84%	Revision 88%	Community engagement 44%	Explicit outcome 76%

Participants noted close discussion with federal member states on the modality of scale up as evidence of devolved decision-making. Participants mentioned that there were several opportunities to revise this decision in which the need for oxygen provision was reaffirmed but the modality was revised. They noted that the initial decision was for WHO to support procurement of oxygen locally. This changed to procurement of cylinders and ventilators and eventually to supporting the establishment of oxygen plants. Participants mentioned limited evidence of community engagement in the decision itself but significant engagement through health messaging after the fact. They cited dashboards as containing evidence of clear outcomes and targets that underlay this decision.

Feasibility 80%	Strengthen Health system 84%	Evidence based 84%

Participants noted that this decision supported strengthening of the health system by fortifying performance of isolation facilities and optimizing case management. They also cited later documents as providing evidence that underlay this decision such as the technical update on survival among hospitalized COVID-19 patients.

Recommendations

A number of recommendations were put forward by participants in order to strengthen the decision-making process within the organisation. They include the following:

- Improve documentation on decision-making deliberations as participants were aware of many discussions that were not reflected in official internal or external records.
- To further strengthen decision mechanisms by clearly outline who is involved, their responsibilities and the mechanisms by which they arrived at the decision.
- Strengthen engagement with affected communities in addition to government officials *during* the decision-making process rather than solely communicating finalised decisions. This will strengthen accountability.
- Consider sustainability as a potential criterion for good decision-making
- Repeat this decision-making exercise on a more routine basis by adopting it as a component of internal evaluation

Annex

Score Criteria

Dimension	Criteria	Description
Transparency	Inclusivity	The extent to which the process was inclusive, reflected in heterogeneity in rank and roles amongst decision makers involved.
	Use of explicit decision-making criteria	The extent to which the goals and objectives of the decision were clearly pre-specified. The absence of post-decision rationalization.
	Following clear process or method	The extent to which a priority setting process was in place, reflected in demonstrated use of priority setting frameworks, decision trees or other mechanism.
	Use of mechanism to publicise rationale	The extent to which clear documentation on the decision exists as well as the method used to communicate decisions.
Contestability	Opportunity for revision	The extent to which there existed scope to revise and overturn a decision including the debating of alternatives and description of how consensus was reached.
	Was the decision devolved?	The degree to which participants in closest proximity to the epidemic (e.g. subnational level) or local technical experts participate in the decision, including consideration of rank.
Accountability	Engagement with affected communities	The degree to which affected communities were involved in the response decision-making including at a minimum whether they were informed of the response activities and what effect this notification had on the communities.
Rigour	Explicit outcome	The extent to which intended outcomes of the decision were clearly articulated, including through setting of targets.
	Feasible outcome	The extent to which feasibility was considered in decision-making including debating of alternatives.
	Strengthens healthcare system	The extent to which the decision was in-line with wider strategy including the strengthening of the health system
	Evidence based	The extent to which the decision was based on strong public health rationale and robust scientific information.
