# Supplementary material for: Auditing the quality of epidemic decision-making in Somalia: a pilot evaluation
Source: BMJ Open. 2023 Jan 3;13(1):e065122. doi: 10.1136/bmjopen-2022-065122 (PMC9815027; doi:10.1136/bmjopen-2022-065122)
Supplement: Supplementary data [file bmjopen-2022-065122supp006.pdf]

BMJ Publishing Group Limited (BMJ) disclaims all liability and responsibility arising from any reliance placed on this supplemental material which has been supplied by the author(s)

Decision-making audit scorecard
CARE Somalia's COVID-19 response

Evaluation study

November 8th -Nov 22nd 2021

Abdihamid Warsame
Faculty of Epidemiology and Population health
London School of Hygiene and Tropical Medicine

21 January 2022

Background

On the 30th of January 2020, the World Health Organization (WHO) declared the emergence of SARS-CoV-2 corona virus as a Public Health Emergency of International Concern (PHEIC) because of evident human-to-human transmission and renamed it COVID-19. On the 16th of March 2020, Somalia reported its 1st COVID- 19 confirmed case, a Somali national with travel history to China. As of the 20th of January 2022, Somalia had 24,261 confirmed cases of COVID-19 with 1335 deaths. The Federal Ministry of Health launched the National COVID-19 Preparedness & Response Plan on the 26th March 2020 with a budget of USD 57 million and estimated caseload of 6,000 confirmed cases.

Somalia's capacity to prevent, detect, and respond to any global health security threat scored six out of 100 on the 2016 Health Emergency Preparedness Index. Poor urban communities, internally displaced persons(IDPs), the elderly, households of minority clans, riverine communities, women and child headed households, and children are most at risk due to social exclusion and decreased accessibility to health services, sanitation and water.

The COVID-19 pandemic weakened Somalia's already fragile health system that has been negatively impacted by protracted conflict, cyclic drought, and locust infestation. The Somali health system has not been able to cope adequately with the demands of a COVID-19 response with poor and few testing facilities, low capacity of health care workers and poor funding. Social cultural practices, rumours, misconceptions, poor hygiene practices and communal caring for the sick in homes and communities, have additionally amplified the spread of the virus.

Methods

The evaluation occurred in Garowe, Somalia from 8- 18th of November 2021. A total of 8 participants from various units within CARE Somalia took place in this evaluation.

The evaluation was undertaken following the standard operating procedures (SOP) developed by the LSHTM research team (See annex) and with the appointment of a CARE focal point. The CARE focal point gathered the necessary background documentation as well as supported the formation of the decision-making committee through recruitment of evaluation participants from the organization. The focal point completed the first draft of the contextual analysis as well as consolidated input from the other participants. He was also instrumental in facilitating the group work sessions.

Critical Decisions

Participants were advised to select important decisions for critical evaluation based on the following criteria on what constitutes a critical decision.

Characteristics

A critical decision is one has one or more of the following characteristics:

I. Consequentiality-: A critical decision is consequential and shapes the response to a significant degree
II. Reversibility-: A critical decision can be difficult to overturn or reverse at least in the short term
III. Strategic: A critical decision entails a significant shift in terms of action taken, resources committed or precedent set
IV. Uncertainty: A critical decision entails a wide range of uncertainty or complex array of options
V. Reputational Risk: A critical decision entails a high level of organisational reputational risk

Decision	Criteria for Selection
i. Adaptation of nutrition programming to COVID-19	☐ Characteristic 1<br>☒ Characteristic 2<br>☒ Characteristic 3<br>☐ Characteristic 4<br>☐ Characteristic 5
ii. Closure of CARE offices and restriction of staff movement	☒ Characteristic 1<br>☐ Characteristic 2<br>☒ Characteristic 3<br>☐ Characteristic 4<br>☒ Characteristic 5
iii. Implementation of COVID-19 project in Sool and Sanaag	☒ Characteristic 1<br>☒ Characteristic 2<br>☒ Characteristic 3<br>☒ Characteristic 4<br>☐ Characteristic 5

Summary Decision Scorecard

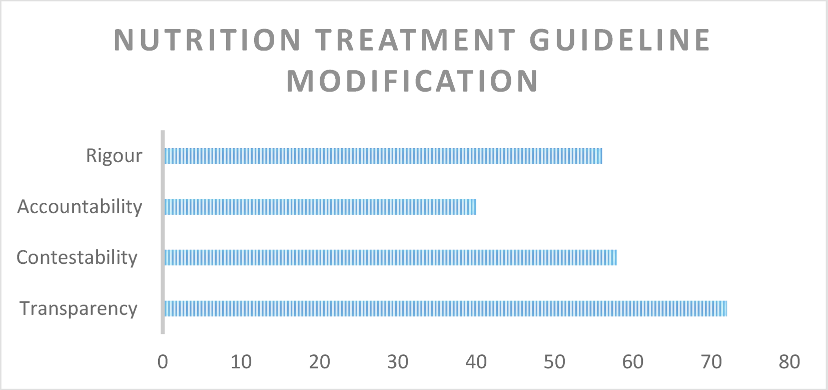

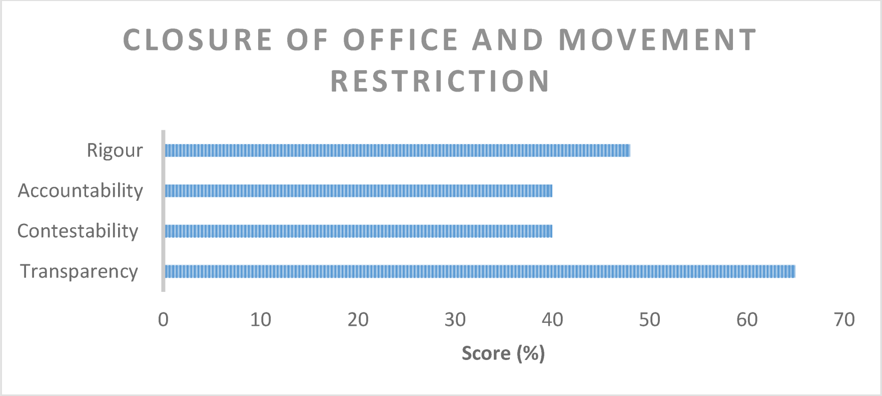

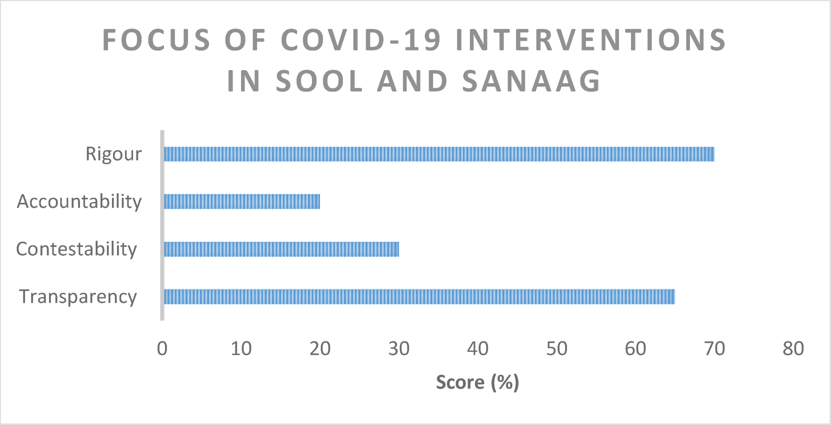

LONDON SCHOOL of HYGIENE &TROPICAL MEDICINE

Detailed Scores & Interpretation

1. Nutrition treatment guideline modification

Inclusivity 72%	Decision criteria 76%	Process 64%	Public rationale 76%

In terms of transparency, the decision to modify nutrition programme guidelines to be COVID-19 compatible was found to be fairly transparent. However, participants described a deficit in accountability as these modifications were reportedly made at the cluster level without the input of all partners. Additionally, participants reported that the affected communities were not consulted and could not feed into this decision-making process.

Devolution 44%	Revision 72%	Community engagement 40%	Explicit outcome 56%

Moreover, participants noted they did not have opportunity to contest these adaptations before they were adopted. They did however agree that there was a clear rationale to minimize transmission risk to the population. Nevertheless, participants did make clear the missed opportunity in engaging with the community to prepare them for these modifications.

Feasibility 55%	Strengthen Health system 60%	Evidence based 53%

Participants expressed concern that these adaptations would reduce patient follow-up, risk missing eligible program beneficiaries and could lower the effectiveness of the program.

2. Closure of Office and movement restriction

Inclusivity 60%	Decision criteria 76%	Process 64%	Public rationale 60%

The closure of CARE offices and restriction of staff movement was done early in the response. The decision was taken by the senior management and communicated via email to all staff. As a result, evaluation participants felt that this approach lessened the transparency as an opportunity was not provided to staff to feed into this decision. There was no clear documentation provided on the deliberations of this decision and the various options considered.

Participants did concede that it was possible that there were prioritization and decision-frameworks utilized but they were not disseminated to all staff.

Feasibility 60%	Strengthen Health system 40%	Evidence based 36%

Participants noted that in terms of supporting evidence, WHO and other international guidelines were possibly followed. No specific document was cited. However, the driving factor was to preserve business continuity and an abundance of caution as there were limited evacuation options for staff who felt ill.

Devolution 36%	Revision 44%	Community engagement 40%	Explicit outcome 60%

Participants mentioned that the decision to close offices was not devolved to sub-offices but was made at the country level. Additionally, there was limited community engagement with participants noting that as this could possibly affect program delivery, there should have been some level of community engagement.

Warsame A, et al. BMJ Open 2023; 13:e065122. doi: 10.1136/bmjopen-2022-065122

LONDON SCHOOL of HYGIENE &TROPICAL MEDICINE

3. Focus of COVID-19 Interventions in Sool and Sanaag

Inclusivity 40%	Decision criteria 80%	Process 80%	Public rationale 60%

CARE has largely been conducting its humanitarian and development programming in rural areas prior to the pandemic. When the pandemic occurred, fundraising and response resources were focused to areas which CARE had a strong field presence. These were in Sool and Sanaag regions in largely rural areas. However, the majority of COVID-19 cases were in urban centres and in higher density areas of the country. Thus there was a mismatch between where resources were directed and where they were most required. Participants explained that as a result of this prioritization of existing programme areas, there was inadequate strategic focus and limited contestability in the decision.

Devolution 40%	Revision 20%	Community engagement 20%	Explicit outcome 60%

Participants reported limited community engagement. However, there was ample opportunities to revise this decision according to some participants. There was also sufficient devolution with the design of the COVID-19 intervention done by both senior management as well as some technical staff based in the sub-offices.

Feasibility 80%	Strengthen Health system 80%	Evidence based 60%

Participants did not feel that this decision strengthened the health system as much as it could have if resources were more precisely directed where needed. They did cite usage of early available WHO guidelines as having a role in how the decision was undertaken. Participants rated the feasibility criteria as high based on the consideration by decision-makers to implement the COVID-19 interventions in existing program areas.

Conclusion

Overall very few primary or secondary documents were specifically cited by participants as evidence for the decision-making criteria. Participants were aware to varying degrees that some documents such as minutes and guidelines did influence decisions but were largely unable to cite them for this evaluation. They instead relied primarily on their recollection of events and discussions with fellow participants to piece together the events that transpired.

Recommendations

Decision-making

In order to improve the quality of decision-making in CARE's COVID-19 response and more broadly in its humanitarian responses, the following recommendations have been put forward:

1) Improve the inclusivity of key critical decision-making through expanding the membership of decision-makers to include junior staff and field staff.
2) Improve trust and buy-in of decisions through strengthening reporting of decision rationale and criteria.
3) Strengthen documentation of response activities to allow for more in-depth real-time or post response evaluation.
4) Consider more critically the relevance of scientific evidence and public health measures to the context rather than blanket adoption.
5) Allow for more contextually specific factors to shape decisions rather than blanket decisions

Evaluation Process

1) Allow for more time pre-evaluation in order for evaluation team to orient themselves with the methodology.
2) Translate evaluation tools and documentations into local language to allow for better understanding and involvement of evaluation participants
3) Allow for remote or asynchronous completion of evaluation tools by participants
4) Broaden the evaluation participants to include non-emergency staff and representatives of the community and local government
5) Consider practise scenario to allow for better uptake by evaluation team prior to commencing evaluation

LONDON SCHOOL of HYGIENE &TROPICAL MEDICINE

Annex

Evaluation Schedule

Step	Action	Responsible	Date
1	Recruitment of evaluation team and signing of consent forms	All participants	November 8th
2	First Part A- Draft of Context Analysis	CARE Evaluation Focal Person	November 11
3	Review of Part A- Context Analysis	All Participants	November 13th
4	Set up Meeting	LSHTM & CARE focal person	November 14th
6	Completion of Part B- Decision Selection	All participants	November 16
7	Decision Ranking Exercise	All Participants as group	November 16
8	Completion of Part C- Scoring of Decisions	All Participants separately	November 17
8	Completion of Part D- Overall Scoring	LSHTM	November 25th
9	Key Informant Interview	All Participants	November 18- 21st

Score Criteria

Dimension	Criteria	Description
Transparency	Inclusivity	The extent to which the process was inclusive, reflected in heterogeneity in rank and roles amongst decision makers involved.
	Use of explicit decision-making criteria	The extent to which the goals and objectives of the decision were clearly pre-specified. The absence of post-decision rationalization.
	Following clear process or method	The extent to which a priority setting process was in place, reflected in demonstrated use of priority setting frameworks, decision trees or other mechanism.
	Use of mechanism to publicise rationale	The extent to which clear documentation on the decision exists as well as the method used to communicate decisions.
Contestability	Opportunity for revision	The extent to which there existed scope to revise and overturn a decision including the debating of alternatives and description of how consensus was reached.
	Was the decision devolved?	The degree to which participants in closest proximity to the epidemic (e.g. subnational level) or local technical experts participate in the decision, including consideration of rank.
Accountability	Engagement with affected communities	The degree to which affected communities were involved in the response decision-making including at a minimum whether they were informed of the response activities and what effect this notification had on the communities.
Rigour	Explicit outcome	The extent to which intended outcomes of the decision were clearly articulated, including through setting of targets.
	Feasible outcome	The extent to which feasibility was considered in decision-making including debating of alternatives.
	Strengthens healthcare system	The extent to which the decision was in-line with wider strategy including the strengthening of the health system
	Evidence based	The extent to which the decision was based on strong public health rationale and robust scientific information.
