# Supplementary material for: Auditing the quality of epidemic decision-making in Somalia: a pilot evaluation
Source: BMJ Open. 2023 Jan 3;13(1):e065122. doi: 10.1136/bmjopen-2022-065122 (PMC9815027; doi:10.1136/bmjopen-2022-065122)
Supplement: Supplementary data [file bmjopen-2022-065122supp002.pdf]

Decision Making Evaluation Instrument

Organization:

Date:

Outbreak:

Part A- Summary of Response

(i) Describe briefly the historical, geographic and health context in which the outbreak is occurring *(maximum 250 words).*

(ii) Briefly describe the course of the epidemic from detection up the present focusing on epidemiology, risk factors and effected populations *(maximum 250 words).*

(iii) Describe what actions your organization has taken in response to the epidemic *(maximum 300 words).*
