# Supplementary material for: Auditing the quality of epidemic decision-making in Somalia: a pilot evaluation
Source: BMJ Open. 2023 Jan 3;13(1):e065122. doi: 10.1136/bmjopen-2022-065122 (PMC9815027; doi:10.1136/bmjopen-2022-065122)
Supplement: Author's manuscript [file bmjopen-2022-065122.draft_revisions.pdf]

BMJ Open is committed to open peer review. As part of this commitment we make the peer review history of every article we publish publicly available.

When an article is published we post the peer reviewers' comments and the authors' responses online. We also post the versions of the paper that were used during peer review. These are the versions that the peer review comments apply to.

The versions of the paper that follow are the versions that were submitted during the peer review process. They are not the versions of record or the final published versions. They should not be cited or distributed as the published version of this manuscript.

BMJ Open is an open access journal and the full, final, typeset and author-corrected version of record of the manuscript is available on our site with no access controls, subscription charges or pay-per-view fees (http://bmjopen.bmj.com).

If you have any questions on BMJ Open's open peer review process please email info.bmjopen@bmj.com

BMJ Open

Auditing the quality of epidemic decision-making in Somalia: a pilot evaluation

Journal:	*BMJ Open*
Manuscript ID	bmjopen-2022-065122
Article Type:	Original research
Date Submitted by the Author:	30-May-2022
Complete List of Authors:	Warsame, Abdihamid; London School of Hygiene and Tropical Medicine Department of Infectious Disease Epidemiology,<br>Ore, Abdulkadir; Care Somalia<br>Azad, Abdullah; World Health Organization Somalia<br>Hassan, Farhan; World Health Organization Somalia<br>Blanchet, Karl; University of Geneva Faculty of Medicine, Geneva Centre of Humanitarian Studies<br>Palmer, Jennifer; London School of Hygiene and Tropical Medicine Department of Global Health and Development,<br>Checchi, Francesco; London School of Hygiene and Tropical Medicine Faculty of Epidemiology and Public Health
Keywords:	Health policy < HEALTH SERVICES ADMINISTRATION & MANAGEMENT, International health services < HEALTH SERVICES ADMINISTRATION & MANAGEMENT, Organisational development < HEALTH SERVICES ADMINISTRATION & MANAGEMENT, Quality in health care < HEALTH SERVICES ADMINISTRATION & MANAGEMENT, Protocols & guidelines < HEALTH SERVICES ADMINISTRATION & MANAGEMENT, INFECTIOUS DISEASES

SCHOLARONE™
Manuscripts

I, the Submitting Author has the right to grant and does grant on behalf of all authors of the Work (as defined in the below author licence), an exclusive licence and/or a non-exclusive licence for contributions from authors who are: i) UK Crown employees; ii) where BMJ has agreed a CC-BY licence shall apply, and/or iii) in accordance with the terms applicable for US Federal Government officers or employees acting as part of their official duties; on a worldwide, perpetual, irrevocable, royalty-free basis to BMJ Publishing Group Ltd ("BMJ") its licensees and where the relevant Journal is co-owned by BMJ to the co-owners of the Journal, to publish the Work in this journal and any other BMJ products and to exploit all rights, as set out in our licence.

The Submitting Author accepts and understands that any supply made under these terms is made by BMJ to the Submitting Author unless you are acting as an employee on behalf of your employer or a postgraduate student of an affiliated institution which is paying any applicable article publishing charge ("APC") for Open Access articles. Where the Submitting Author wishes to make the Work available on an Open Access basis (and intends to pay the relevant APC), the terms of reuse of such Open Access shall be governed by a Creative Commons licence – details of these licences and which Creative Commons licence will apply to this Work are set out in our licence referred to above.

Other than as permitted in any relevant BMJ Author's Self Archiving Policies, I confirm this Work has not been accepted for publication elsewhere, is not being considered for publication elsewhere and does not duplicate material already published. I confirm all authors consent to publication of this Work and authorise the granting of this licence.

For peer review only

Auditing the quality of epidemic decision-making in Somalia: a pilot evaluation

For peer review only

Abdihamid Warsame[1]
Abdulkadir Ore[2]
Abdullah Azad[3]
Farhan Hassan[3]
Karl Blanchet[4]
Jennifer Palmer[5]
Francesco Checchi[1]

[1]Faculty of Epidemiology and Population Health, The London School of Hygiene & Tropical Medicine, London, United Kingdom.

[2]CARE International Somalia. Garowe, Somalia

[3] Health Emergencies Unit. World Health Organization Somalia. Mogadishu, Somalia

[4]Geneva Centre of Humanitarian Studies, Faculty of Medicine, University of Geneva, Geneva, Switzerland

[5]Faculty of Public Health and Policy, The London School of Hygiene & Tropical Medicine, London, United Kingdom.

Corresponding author:

Abdihamid Warsame

Faculty of Epidemiology and Population Health, The London School of Hygiene & Tropical Medicine, London, United Kingdom.

Email address: Abdihamid.warsame@lshtm.ac.uk

Abstract

Objective

To assess decision making quality through piloting an audit tool among decision makers responding to the COVID-19 epidemic in Somalia.

Setting

We utilized a mixed methods programme evaluation design comprising quantitative and qualitative methods. Decision-makers in Somalia piloted the audit tool generating a scorecard for decision-making in epidemic response. They also participated in key informant interviews discussing their experience with the audit process and results.

Participants

A total of 18 decision-makers from two humanitarian agencies responding to COVID-19 in Somalia were recruited to pilot the audit tool.

Outcome measures and analysis

We used thematic analysis to assess the feasibility and perceived utility of the audit tool by intended users (decision-makers). We also calculated Fleiss' Kappa to assess interrator agreement in the audit scorecard.

Results

The audit highlighted areas of improvement in decision-making amongst both organisations including in the dimensions of accountability and transparency. Despite the audit occurring in a highly complex operating environment, decision-makers found the process to be feasible and of high utility. The flexibility of the audit approach allowed for organisations to adapt the audit to their needs. As a result, organisation reported a high level of acceptance of the findings

Conclusion

The pilot evaluation illustrates that it is possible to systematically asses decision-making quality in an ongoing epidemic within a humanitarian setting. Improving decision-making quality can contributes to better response outcomes including more efficient allocation of resources, greater coordination and saved lives

Introduction

Evaluating decision-making has been recognized as essential to improving health outcomes in a number of contexts[4]. In humanitarian and crisis contexts good decisions at program, sector and response level are especially critical to saving lives and improving response[5]. Despite recent calls for concerted evaluation of decision-making in these settings[6,7], the literature is still sparse. In crisis-affected settings experiencing epidemics, evaluations to date have primarily focused on establishing the extent to which epidemic response outcomes (for example reduced transmission, improved case management) have been attained. Less attention has been given to evaluate the processes underlying these outcomes (for example how response activities were

For peer review only

decided and implemented) [8]. Process evaluations have largely been conducted in high-income countries or after high-profile epidemics to retrospectively determine which decisions led to response failures[9–11].

The need to improve decisions in epidemic settings is especially relevant considering the ongoing COVID-19 pandemic, in which decision-makers contend with a plethora of competing emergencies[12]. Decision support tools have been developed for a variety of settings and purposes but are particularly ubiquitous in corporate business management[13] and in the pharmaceutical and health technology sector[14] for example in the development and licensing of medicinal products, equipment and diagnostics. They are less frequent in humanitarian contexts where they focus primarily on supporting decision-makers in optimizing selected response interventions through for example specifying target populations or modalities of delivery[15,16]. To our knowledge there does not exist an evaluation tool that examines the quality of decision processes within epidemic settings. In a previous study in Somalia, we described factors relevant to COVID-19 decision-making processes; results suggested a need for such a tool.[12]

We thus developed a decision-making audit tool to support epidemic responders in assessing and improving the quality of organisational decision processes. Here, we report on a pilot application of this tool amongst two epidemic response organisations in Somalia, a country grappling with the ongoing COVID-19 pandemic and ongoing humanitarian crises [17].

Aim and Objectives

The overall aim was to evaluate the utility and feasibility of a decision support tool for epidemic responders in humanitarian settings.

The specific objectives were to: (i) document the implementation of the tool among select epidemic responders in Somalia; (ii) generate epidemic decision-making scores using the decision support tool; and (iii) explore the feasibility and utility of the tool through key informant interviews of epidemic responders involved in the pilot.

For peer review only

Methods

Study Design

This study used a mixed methods programme/response evaluation design. It comprised quantitative and qualitative data collection to both assess the decision-making in epidemic response as well as the feasibility and perceived utility of the tool by intended users (decision-makers). We then revised the tool based on this data. The final structure of the decision-making evaluation tool was determined from input collected during the pilot evaluation.

Patient or public involvement

Neither patients or the public were involved in the design of this study.

Description of the tool

We developed an evaluation tool, protocol and Standard Operating Procedure (SOP) (Supplementary Files 1-4) founded on previous reviews [18] and extensive fieldwork in an epidemic setting[12]. This foundational work resulted in findings of very low coverage of epidemic evaluations globally, limited focus on decision processes, lack of standardized evaluation methods as well as an absence of a comprehensive evaluation framework suitable for epidemic response. As a result, we developed an adaptive epidemic response (ARE) evaluation framework[19] and drawing from an assessment of the COVID-19 response in Somalia[12], we derived a decision-making framework. The tools and SOPS are based on this decision-making framework. The tool is comprised of three sections and is summarized below (Figure 1).

The first section (Part A) of the tool is the context analysis. which requires information on the historical, geographic and health context in which the epidemic is occurring.

The second section (Part B) entails identification of critical decisions in the response to the specific epidemic. These critical decisions are identified and selected with reference to five Critical Decision Characteristics that differentiate critical decisions from minor or low impact decisions[5] (Table 1).

For peer review only

Table 1. Characteristics of 'critical' decisions

Characteristic	Definition
Consequential	Decision shapes the response to a significant degree
Not reversible	Decision is difficult to overturn or reverse, at least in the short term
Strategic	Decision involves substantial shift in terms of action taken, resources committed or precedent set
Uncertain	'Decision is made in context of substantial uncertainty with complex array of options
Reputationally risky	Decision entails a high level of organisational reputational risk

In the third section of the tool (Part C), users assess each of the selected decisions against the criteria for 'quality' decision-making. Although there is no agreed definition of a good quality decision[20], we have derived a number of defining criteria from previous published research on health prioritization[21], organizational decision-making[20,22,23] as well as decision-making in emergencies[24]. The 11 criteria are grouped into 4 dimensions: transparency, contestability, accountability and rigour (Table 2).

Table 2. Criteria for assessing decision quality

Dimension	Criteria	Description
Transparency	Inclusivity	The extent to which the process was inclusive, reflected in heterogeneity in rank and roles amongst decision makers involved.
	Use of explicit decision-making criteria	The extent to which the goals and objectives of the decision were clearly pre-specified. The absence of post-decision rationalization.
	Following clear process or method	The extent to which a priority setting process was in place, reflected in demonstrated use of priority setting frameworks, decision trees or other mechanism.
	Use of mechanism to publicise rationale	The extent to which clear documentation on the decision exists as well as the method used to communicate decisions.
Contestability	Opportunity for revision	The extent to which there existed scope to revise and overturn a decision including the debating of alternatives and description of how consensus was reached.
	Was the decision devolved?	The degree to which participants in closest proximity to the epidemic (e.g. subnational level) or local technical experts participate in the decision, including consideration of rank.

For peer review only

For peer review only - http://bmjopen.bmj.com/site/about/guidelines.xhtml

**Accountability**	Engagement with affected communities	The degree to which affected communities were involved in the response decision-making including at a minimum whether they were informed of the response activities and what effect this notification had on the communities.
**Rigour**	Explicit outcome	The extent to which intended outcomes of the decision were clearly articulated, including through setting of targets.
	Feasible outcome	The extent to which feasibility was considered in decision-making including debating of alternatives.
	Strengthens healthcare system	The extent to which the decision was in-line with wider strategy including the strengthening of the health system
	Evidence based	The extent to which the decision was based on strong public health rationale and robust scientific information.

Users are required to rate on a Likert scale (a type of linear rating scale commonly used to measure respondents' opinions or attitudes), the strength of evidence supporting the fulfilment of each criterion. They are also expected to provide reference to documentary or observational evidence to support their rating. Users' individual scores are then aggregated and a summary score is generated.

Data Collection

Audit

We invited three organisations actively engaged in the COVID-19 response in Somalia to pilot the audit tool. After separate presentations in which the protocol and study objectives were explained by the first author, we partnered with two organisations (WHO Somalia and CARE Somalia) in September 2021. Both organisations then nominated evaluation focal persons tasked with recruiting relevant colleagues (a 'decision-making committee'), gathering the necessary documentation and completing part A of the tool. Focal persons were instructed to recruit colleagues who had an active role in decision-making within the response as well as to compile key documents which informed or documented decisions. The location and modality of the pilot (combination of face to face and remote sessions) and timeline were jointly determined by the evaluation focal persons and the first author. Study information and consent forms were shared and written consent was obtained from all participants in the pilot.

For peer review only

The decision-making audit and feasibility assessment took place in Garowe, Somalia from November 8-22nd 2021 with CARE Somalia and in Mogadishu from November 22- December 8 with WHO Somalia. The pilot was led by each organisation's evaluation focal persons with the facilitation of the first author. Each evaluation was comprised of 3 group sessions interspersed with individual sessions (Figure 2). The first group session introduced the evaluation approach, tool, timeline and expected outputs. Participants were then asked to review Part A (context analysis) of the tool and incorporate any changes before the next group session. In the second group session, participants were introduced to Part B (selection of critical decisions) and were tasked with individually generating their perceived list of critical decisions. In the third group session, participants were asked to each present and advocate for their selection of critical decisions to the wider group. The group was then tasked with forming a consensus on at least 3 of the decisions to carry forward to the last stage. After consensus was reached participants were asked to complete Part C of the tool for each decision in which they assessed the availability and strength of evidence supporting the fulfilment of various quality decision-making criteria. Evaluation focal persons and the LSHTM researcher then aggregated the scores and created a draft scorecard.

Evaluation

All evaluation participants were then invited to take part in a key informant interview to expound on their selected decisions and explore their views on the evaluation process and tools. After the key informant interviews, the draft scorecard was shared with all participants to provide individual written feedback on the results. The final scorecard was then developed by the evaluation focal persons and first author and shared with the senior management of the respective organisation. Finally, key informant interviews were conducted by the first author with senior managers who were not involved in the other aspects of the audit and evaluation to solicit their views on the results and their understanding of the process. The tool and related documents were then updated based on pilot findings.

For peer review only

A total of 18 key informant semi-structured interviews were conducted in English or Somali by the first author either face-to-face or via Zoom (Table 3). The topic guide covered participants' views on the feasibility of the audit process including time and human resources required, the utility of the tool in understanding and improving decision making as well as recommendations for future iterations.

Table 3 Data collection by method and type of participant or setting

Primary Data collection	Type of Participants	Number of Participants
**Key informant interviews**	WHO Evaluation team	7
	WHO Staff- Other	2
	WHO Senior management	2
	CARE Somalia Evaluation Team	5
	CARE Somalia Senior management	2
**Group Sessions**	WHO Evaluation Team	7
	CARE Evaluation Team	6

Interviews were recorded for transcription and analysis purposes. Each interview took approximately 30-45 minutes and participants were given the option of complete or partial anonymity in which they considered whether they would like their name or only their role to be published. A total of 13 participants took part in the group sessions and notes were taken by the first author. All participants in the CARE evaluation were national staff members while the majority of WHO participants were international (85%).

For peer review only

Data Analysis

Generation of audit tool scoring

The scorecard for the audit was produced by calculating for each of the 11 criteria assessed, the average of the individual participants scores. Average scores were also presented according to the four dimensions listed in table 2.

Evaluation of the audit implementation

In order to evaluate the implementation of the audit tool as it relates to the feasibility and utility, interview transcripts and group session notes were analysed using a thematic approach[25] using Nvivo software. Emerging themes were grouped into two categories: those relating to identified critical COVID-19 decisions and those relating to the implementation of the evaluation. Lastly the validity of the tool was evaluated. The interrater agreement of each organisation's scoring was calculated using Fleiss' Kappa.

In the following results section, we focus on the implementation of the audit while details of specific decisions can be found within the decision-making scorecards in the supplemental material (Supplementary File 5, Supplementary File 6).

Results

Implementation of the decision-making audit

Participants in both organisations opted for a mixture of face-to-face and remote (video conference) data collection citing the busy schedule and geographic dispersion of the participants. Although preparatory material including documentation was shared prior to the arrival of the LSHTM focal person, some participants were not immediately aware of the purpose of the evaluation. The delayed setup of shared folders containing key evaluation documentation proved difficult for participants' engagement with the evaluation. Furthermore, the period in which this evaluation took place coincided with multiple projects including end-of-year evaluations, annual planning and multiple pre-scheduled activities, hindering the timely recruitment of participants. Many participants were not able to be physically present in the location of the evaluation due to competing engagements but were nevertheless present through remote means.

Decision Selection

CARE participants had difficulty identifying critical decisions with individual participants submitting on average only 1 critical decision for consideration. However, consensus was

For peer review only

reached fairly quickly once the proposed decisions were deliberated by the wider group. In contrast, WHO evaluation participants put forward 15 decisions for consideration. Consensus took longer to achieve as participants vigorously advocated for their proposed individual decisions. Eventually the group reached consensus on 4 decisions to proceed to the next stage (Table 4). In both groups, moderate reference was made to the Critical Decision Characteristics (Table 1) with participants arguing for decisions in their particular area of work. Participants in WHO were particularly inclined to view decisions in terms of their alignment with the Incident Management Support Team (IMST) Pillars[26]. Additionally, there was an effort by some participants to reach consensus by collapsing together multiple decisions into a single decision in order to capture all opinions. In both WHO and CARE evaluations, participants focused on positive decisions (i.e. a decision to take an action) and may have overlooked negative decisions (i.e. decisions not to take action).

Table 4. List of selected critical decisions by organisation

CARE Somalia	WHO Somalia
Closure of offices and restriction of staff movement	Scale up of case management through improving access to therapeutic oxygen
Modification of Nutrition programme guidelines to be COVID-19 sensitive including changing patient assessment, facility management and outreach procedures	Establishment of 3 key PCR labs in Mogadishu, Garowe and Hargeisa to strengthen diagnostic and surveillance capacity
Scale up of COVID-19 response activities such as community outreach, contact tracing, provisioning of IPC supplies in Sool and Sanaag Regions	Implementation of the Incident Management Support Team to coordinate COVID-19 response
	Establishment of rapid response teams to scale up surveillance capacity within high priority districts

Decision Scoring

Participants in the CARE evaluation on average scored their decision-making quality lower than those of WHO. Both organisations scored lowest in accountability to target populations The scoring of WHO participants on the decision to focus on case management through oxygen scale up demonstrates the low scoring of accountability relative to the other dimensions of quality

For peer review only

decision-making (Figure 3). Full details can be found in the supplementary materials (Supplementary File 5, Supplementary File 6).

Participants in CARE scored their decisions lower in contestability compared to WHO. Both organisations rated themselves highly in transparency, particularly in the inclusivity criteria. However, the key stakeholder mentioned under these criteria differed, with CARE highlighting the need to improve inclusive decision-making within the organisation (national staff vs country office senior management) while WHO referred to inclusivity with government authorities. Lastly, CARE participants rated their decision-making as less rigorous than WHO's, citing their unfamiliarity with the evidence underlying the decisions. By contrast, WHO participants cited use of the latest scientific findings and alignment with health systems strengthening frameworks.

Interrater agreement

There was very low interrater agreement amongst participants in both WHO (Kappa = -0.000948, P= 0.977) and CARE (Kappa = 0.0684, P= 0.172) evaluation groups with a slightly higher interrater agreement amongst CARE participants, indicating high heterogeneity of scores.

Perceptions of the audit tool

Utility

Participants described clearly seeing the purpose and value of the audit. For example, one participant from WHO said, "*We learned that decisions are not just simply taken as decisions, but you have to have a clear decision-making process, a methodology, or criteria, or something, but not necessarily that we just come and say, "Oh, let's do this and that"*. Participants articulated the ongoing need for such an exercise, arguing: "*If there's no understanding of how a decision happened, there's no way to improve on it" (WHO Participant 5).* Critically, they understood that this exercise was meant as an internal evaluation: *"It was really for us to be able to critically evaluate ourselves as an organisation" (CARE Participant 3).* Some participants also saw the potential utility in a wider range of contexts, beyond epidemics: "*I think [the audit tool] can apply to any decision in humanitarian emergency response" (WHO Participant 2).*

Participants mentioned learning new things about the decision process including recognizing neglected characteristics of good decision-making:

> *"When I was trying to fill in some of the scores, I realized that these are very important [aspects] that were neglected before. For example, devolving certain decisions, it is very important. Not just like you sit up at national level, you decide for anyone and you decide for people at all levels, I think if we devolve certain functions it is good". WHO Participant 1*

They also were able to identify areas for improvement. "*I think for accountability most of the evidence was very minimal. It just got me thinking of ways in which we can involve the communities in decision-making." WHO Participant 4*

Participants also highlighted that the audit was useful in allowing them to be more reflective. *"It helped me look at things objectively. If I was not part of the Incident Management Support Team (IMST) would I have advocated for this decision or not?" WHO Participant 4.* They highlighted the relevance of the decision-making critical characteristics as useful in their work and recognised these as reflective of best-practice: *"I think the tool helped a lot to give me a reflection as to what should be done. I think I learned a lot going through the questions." WHO Participant 1*

Participants mentioned the gradual understanding they accrued throughout the audit process as strengthening their engagement with the audit. *I think some of it was clear, but some of it was also not very clear. I think I understood the methodology with time, of course, when you explained and when we were having the group discussions. That is why in the last forms, I revised and added a few components and sent back to you. I think as usual when people read something, it might not be clear in the first place, but when you explain it, it becomes clear. WHO Participant 4*

Participants also noted the benefit of the group format in deriving new insights and creating a useful forum for reflection to review decisions that otherwise didn't exist in their typical organisational practices:

> *"We all had our own ideas, but when we met as a group, there were a few other things that came out that probably I didn't think about as an individual, which were very valid". WHO Participant 4*

For peer review only

"I also liked that we were able to meet as a group and look at these things because to be very honest, I don't feel like we have that as a group, as an IMST team to review the decisions that we made or look at what we could have done differently, or whether we felt this decision was important or not. This was a very unique exercise and I'm glad that we got an opportunity to do this." WHO Participant 7

Participants expressed their commitment to use the lessons learned in future decision making:

"I will inform my colleagues, 'Please, let us have certain criteria to use, let us make our methodology clear, and let us document everything,' because at some point down the road, we should know our decision-making process. This has helped a lot, and will change a lot in the future." WHO Participant 5

However, participants mentioned that there was a certain element of subjectivity which was difficult to overcome:

"It might be very difficult to understand exactly the reality on the ground, because it depends on the person. Not only the person but also, the involvement of the person in the response activity, and also the understanding of the context. If you are someone who is quite new to Somalia, and maybe who doesn't have detailed involvement, you might give a different ranking than a person who's spent more time in Somalia and directly engaged in response activity". WHO Participant 2

Feasibility

In terms of the audits' feasibility, participants expressed that they were easily able to comprehend the process. *"[The tool] was very understandable and also, it was very user friendly" CARE Participant 1*. However, a few participants noted some areas for improvement: *"I'm looking at this tool being used for an outbreak. If that is to happen, as I said, we need to really condense some aspects, and lump some of these criteria together to cut on time". CARE Participant 3*

In terms of time, participants expressed that the duration of the audit was acceptable but that the time of year chosen for the implementation could have been improved. *"in terms of length of the time, this is fine. This amount of time we need to give. The issue is the timing of the programming, because in some of the months, we are very busy, like last quarter of the year we are very busy". CARE Participant 5*

For peer review only - http://bmjopen.bmj.com/site/about/guidelines.xhtml

As a result, some participants expressed their confidence that they could implement this audit independently at a more opportune time. However, others expressed their preference for this work to be led by an independent evaluator.

> *As the independent outsider, everybody can open up to you, and this is the best advantage I can see. I also saw something similar in my previous organization, that again, it is the power dynamic or the personality thing that not everybody speaks openly, or the culture doesn't allow it but when somebody from outside comes, everybody opens up. At the end, you come up with some really good findings and recommendations" WHO Participant 1*

In their engagement with the audit tool, participants faced a challenge in coming to a consensus on the limited number of decisions to score. *"Considering the response that we are doing in the country, it might be a bit challenging to come up with one or two out of so many critical decisions." WHO Participant 2*

Additionally, there were challenges in accessing documentation relevant to the audit. *Maybe the big challenge is getting the evidence, documentation, preparing the documentation, extracting the email, finding the document, it might be time-consuming, WHO Participant 3*. Where documents did exist, some participants stated that not all staff may have access. Lastly, some participants highlighted language or conceptual understanding barriers as challenges in the audit. *"At some point, I felt like we were not looking at the decisions, but we were looking at pillars. WHO Participant 4*

<u>Panel selection</u>

Participants noted that the composition of the audit participant panel had the potential to affect the audit outcome because of a lack of detailed knowledge on how decisions were made. "*If somebody is not involved in a certain pillar, they would struggle to look for evidence or they might not be able to direct somebody to a particular document". WHO Participant 2*

They mentioned that all who were part of the audit had some role to play in COVID-19 decision making but conversely not all who had decision-making roles participated in the audit.

For peer review only

Some participants highlighted the potential of overlooking critical decisions when the primary responsible staff member was not present in the audit.

> *"later I thought about COVID-19 vaccine, and I wondered if we could have included the focal person and whether they would have felt that the introduction of the COVID-19 vaccine into the country was a critical decision. I thought about it and I thought maybe nobody mentioned it, or nobody talked much about it during the discussion because the focal point was not there. WHO Participant 4*

Participant Recommendations

Participants made several recommendations to improve the utility and feasibility of the audit for future iterations. These included broadening the scope of participants by more clearly outlining who should participate.

Participants also suggested incorporating the emergency response cycle into the evaluation tool. *"there are emergency response cycle phases such as preparedness, implementation, monitoring, and evaluation […] There are critical decisions at each phase which need to be evaluated". WHO Participant 2*

They also made several suggestions to improve the implementation including ensuring more face-to-face rather than virtual interactions amongst participants and undertaking a joint review of documentation to improve participant familiarity with the available documents.

Views of senior management

Senior management largely agreed with the findings of their respective scorecards. They did however, provide further detail and rationale for some aspects of the selected decisions. For example, senior managers in both organisations cited mitigating factors in explaining lower-than-expected scoring in some dimensions. Referring to closure of offices and restriction of staff movements, one CARE senior manager explained:

> *"when the first case of COVID-19 was reported in Somalia in March, I think it was around 16, 17. There was a bit of panic in that it was not business as usual. When it comes to between life and death, certain positions have to be taken. As a result of that, yes, the senior management team made a decision to ensure that because we are accountable to everyone and every staff, and every staff exposed to any risk as a result of negligence of the organization, then the organization takes responsibility." Senior Manager CARE 1.*

For peer review only

A WHO senior manager explained the circumstances behind a decision to increase testing capacity:

> *"Obviously there's scope for improvement, but sometimes when we are in a pressured situations and we see that the PCR based labs are overwhelmed and at the peripheral level there is no access or testing facilities are not there, and the GeneXpert machines are also not functioning, then sometimes we make decisions and implement swiftly, and then [later] we provide other evidence and information of the value and effectiveness of those strategies". WHO Senior Manager 1*

They did however agree on gaps highlighted through the scorecard stating: "*I would say that [decision-making] can become more inclusive. I think that we are just talking internally amongst ourselves and we are not engaging sufficiently with our government counterparts. I think that that's a fundamental mistake that we are doing, and we continuously do so, because of maybe convenience, because of maybe comfort." WHO Senior Manager 2.*

Discussion

This pilot evaluation of a tool to audit decisions taken during epidemic response demonstrates that the tool can be successfully deployed even in the midst of an ongoing response and even in circumstances where responders are dealing with unusually high workload. The audit also elicited very positive user feedback with participants expressing a willingness to implement it in future epidemic responses. Importantly, participants viewed the audit as reflecting good decision-making practise indicating high levels of acceptability.

As populations affected by epidemics continue to grow[27], there is an urgent need to improve epidemic response particularly through improved decision-making. Much of the focus has been on improving decision outcomes and less attention has been paid to decision-making processes[28]. However, strengthening decision processes can improve decision-making quality[3] and, accordingly, outcomes[29] including more efficient allocation of resources, improved accountability and greater coordination.

The literature on decision-making in emergencies has been underpinned by an analytically oriented[30] conceptualization of decision making in which the 'correct' decision is sought[31,32]. This

For peer review only

is in contrast to the process conceptualization in which the decision is pursued correctly. Towards this end, decision support approaches have been developed to optimize the decision-making process to achieve efficient[5] and timely decisions[33] but have largely not considered the aspect of quality. The evaluation of this tool within the COVID-19 response in Somalia contributes towards filling this gap.

The audit sheds light on shortcomings in the quality of decision processes within piloting organisations and provided participants opportunity for reflection and key areas for improvement. The tool was found to be highly adaptable as it allowed organizations to evaluate decisions that were considered significant by their staff. Nevertheless, while the audit instructed organisation to select participants that were largely reflective of staff making or implementing response decisions, some participants expressed reservations about the final composition of the evaluation panel. The scoring of some decision dimensions was however largely reflective of the panel's experiences and characteristics. For example, amongst CARE participants, the lower scoring on the contestability of some decisions might be due to their roles as national staff who may not have had sufficient opportunity to contest decisions. The rollout of the tool allowed for gradual understanding to develop and for participants to question their basic assumptions. For example, participants were able to acknowledge elements that were absent from their decision-making process. They were also surprised by the paucity of documentary evidence within their organisations and were able to contrast this with their initial self-assessed high scoring. The lower than expected agreement amongst raters could possibly due to lack of consensus on what various levels of evidence represented. While limited documentation does not necessarily equate with poor decision processes, it does make subsequent evaluations more challenging.

Furthermore, the audit tool was found to be highly flexible as users highlighted its potential utility in assessing the quality of decision-making in broader humanitarian response by generating a quantitative measure of decision quality that can allow for tracking over time. In addition to the retrospective assessment of decisions, the tool can also be used in real time to improve decision-processes.

For peer review only

Limitations

Although this pilot was undertaken within two organisations, it assessed decision-making related to a single epidemic within one country. While this pilot study was led by the first author who was external to both organisations, future iterations led entirely by internal staff members may be more vulnerable to censorship if results reveal low decision quality. However this is a challenge that is present in global health arena in general[34] and a number of resources have been published to strengthen independence of evaluations[35–37]. Additionally, much of the scoring relied on the subjective assessment of the individual rater and may thus have been reflected in the lower than expected interrater correlation. Furthermore, this pilot focused on face validity (the extent to which a tool appears to measure a concept) rather than on content validity which requires further exploration and methods. Additionally, we did not use quantitative methods such as factor analysis to reduce the number of items within the critical decision criteria as there were too few items. Instead we relied exclusively on direct feedback from the participant interviews. Definitions for the decision- making criteria were not exhaustive and could have been further expanded. For example with regards to community engagement criteria we provided a minimum definition rather than broader definition[38]. Finally, the methods described in this study assessed only organisational decision-making rather than individual and as such cannot be used to assess the decision-making of individual epidemic responders.

Recommendations

The audit can be further piloted in a wider range of crisis settings and amongst different response actors to ascertain its feasibility and utility in diverse settings. Additionally, the audit should be conducted internally by response actors in order to compare how the audit is implemented when it is entirely independent of an external facilitator. Furthermore, the audit should be conducted periodically in order to determine whether there has been a quantitative change in the decision quality scoring. Lastly, the audit tool could also be integrated within the WHO recommended intra-action reviews[2] as well as after action reviews[1] for health emergencies.

For peer review only

Conclusion

Strengthening decision-making processes is key to realizing the objectives of epidemic response. This pilot evaluation contributes towards this goal by the testing what, to our knowledge, may be the first tool designed specifically to assess quality of decision-making processes in epidemic response. The tool has proven feasible and acceptable in assessing decision-making quality in an ongoing response and has potential applicability in assessing decision-making in broader humanitarian response.

For peer review only

Declarations

Contributorship statement

This study was designed by AW and, FC. The data collection was undertaken by AW, AO, FH and AA. The analysis was undertaken by AW with input from KB, FC, and JP. The first draft was written by AW. All authors have contributed significantly to the final draft.

Competing interests

We declare no competing interests.

Funding

This work was supported by UK Research and Innovation as part of the Global Challenges Research Fund, grant number ES/P010873/

Data sharing statement

No additional data available

Ethics approval and consent to participate

Ethical approval for this study was obtained from the National Institute of Health Research of the Federal Republic of Somalia (Ref: NIHS0102208) and the ethics review committee of the London School of Hygiene & Tropical Medicine (Ref: 26369). Informed written consent was taken from all key informants.

Acknowledgements

We would like to acknowledge Md Mamunur Malik WHO country representative and Iman Abdullahi CARE country representative for agreeing to pilot this tool in their respective organisations. We would like to thank participants for sharing their insights.

Reference

1. WHO. The global practice of after action review [Internet]. Geneva; 2019 [cited 2022 Feb 16]. Available from: https://www.who.int/publications/i/item/WHO-WHE-CPI-2019.9

2. Mayigane LN, de Vázquez CC, Vente C, Charles D, Copper FA, Bell A, et al. The necessity for intra-action reviews during the COVID-19 pandemic. Lancet Glob Heal [Internet]. 2020 Dec 1 [cited 2022 Feb 16];8(12):e1451. Available from: /pmc/articles/PMC7544463/

3. George B, Desmidt S. Strategic-Decision Quality in Public Organizations: An Information Processing Perspective: https://doi.org/101177/0095399716647153 [Internet]. 2016 May 4 [cited 2022 Feb 14];50(1):131–56. Available from: https://journals.sagepub.com/doi/10.1177/0095399716647153

4. Glarum J, Adrianopoli C. Decision Making in emergency management. 1st ed. Oxford: Elsevier; 2020.

5. Cosgrave J. Decision making in emergencies. Disaster Prev Manag An Int J. 1996 Oct 1;5(4):28–35.

6. Campbell L, Knox P, Alnappaper C. Making operational decisions in humanitarian response: A literature review [Internet]. 2018 [cited 2020 Jun 3]. Available from: https://www.alnap.org/our-topics/leadership.

For peer review only - http://bmjopen.bmj.com/site/about/guidelines.xhtml

7. Knox Clarke P, Campbell L. Decision-making at the sharp end: a survey of literature related to decision-making in humanitarian contexts. J Int Humanit Action [Internet]. 2020 Dec 15 [cited 2021 Jun 25];5(1):1–14. Available from: https://doi.org/10.1186/s41018-020-00068-2

8. Warsame A, Murray J, Gimma A, Checchi F. The practice of evaluating epidemic response in humanitarian and low-income settings: a systematic review. BMC Med. 2020;18(1).

9. Horton R. Offline: The mistakes we made over Ebola [Internet]. Vol. 394, The Lancet. Lancet Publishing Group; 2019 [cited 2021 Apr 19]. p. 1494. Available from: www.thelancet.com

10. S. M, D. S, M.A. P, A.K. J, C. C, S. D, et al. Will Ebola change the game? Ten essential reforms before the next pandemic. the report of the Harvard-LSHTM Independent Panel on the Global Response to Ebola. Lancet [Internet]. 2015;386(10009):2204–21. Available from: http://www.journals.elsevier.com/the-lancet/

11. García-Basteiro A, Alvarez-Dardet C, Arenas A, Bengoa R, Borrell C, Del Val M, et al. The need for an independent evaluation of the COVID-19 response in Spain [Internet]. Vol. 396, The Lancet. Lancet Publishing Group; 2020 [cited 2021 Mar 18]. p. 529–30. Available from: http://ees.elsevier.com/thelancet/www.thelancet.com

12. Warsame A, Fuje M, Checchi F, Blanchet K, Palmer J. Evaluating COVID-19 decision-making in a humanitarian setting: the case study of Somalia. Plos Glob Public Heal. 2022;

13. Stenfors S, Tanner L, Syrjänen M, Seppälä T, Haapalinna I. Executive views concerning decision support tools. Eur J Oper Res. 2007 Sep 1;181(2):929–38.

14. Bujar M, McAuslane N, Walker SR, Salek S. Evaluating quality of decision-making processes in medicines' development, regulatory review, and health technology assessment: A systematic review of the literature. Front Pharmacol. 2017 Apr 10;8(APR):189.

15. Immunization, Vaccines and Biologicals Vaccination in acute humanitarian emergencies: a framework for decision making. 2013 [cited 2022 Feb 13]; Available from: http://www.who.int/immunization/sage/sage_wg_hum_emergencies_jun11/en/

16. Vargas Florez J, Lauras M, Okongwu U, Dupont L. A decision support system for robust humanitarian facility location. [cited 2022 Feb 11]; Available from: https://hal.archives-ouvertes.fr/hal-01611088

17. UNOCHA. Humanitarian Response Plan Somalia Humanitarian Programme Cycle 2021 Issued February 2021 [Internet]. 2021 [cited 2021 Apr 22]. Available from: www.unocha.org/somalia

18. Warsame A, Blanchet K, Checchi F. Towards systematic evaluation of epidemic responses during humanitarian crises: A scoping review of existing public health evaluation frameworks. BMJ Glob Heal. 2020;5(1).

19. Warsame A, Blanchet K, Checchi F. Towards systematic evaluation of epidemic responses during humanitarian crises: A scoping review of existing public health evaluation frameworks. BMJ Glob Heal. 2020 Jan 30;5(1):e002109.

20. Wood NL, Highhouse S, Brooks M, Chen Y. INDIVIDUAL DIFFERENCES IN DECISION-MAKING STYLES AS PREDICTORS OF GOOD DECISION MAKING. 2012;

21. Kapiriri L, LaRose LB. Priority setting for disease outbreaks in Uganda: A case study evaluating the process. Glob Public Health. 2019;14(2):241–53.

22. Peniwati K. Criteria for evaluating group decision-making methods. Math Comput Model. 2007 Oct 1;46(7–8):935–47.

For peer review only

23. Bang D, Frith CD. Making better decisions in groups [Internet]. Vol. 4, Royal Society Open Science. Royal Society Publishing; 2017 [cited 2021 Jun 30]. Available from: /pmc/articles/PMC5579088/

24. Knox Clarke P. How can we improve decision-making in humanitarian coordination? [Internet]. 2016 [cited 2020 Aug 27]. Available from: www.alnap.org/coord-meeting.

25. Bryman A. Social Research Methods. Oxford; 2012.

26. World Health Organization (WHO). Emergency Response Framework Second Edition. Vol. 69, Taiwan Review. 2017. p. 76.

27. OCHA. Global Humanitarian Overview 2021 [EN/AR/FR/ES] - World | ReliefWeb [Internet]. [cited 2021 Mar 18]. Available from: https://reliefweb.int/report/world/global-humanitarian-overview-2021-enarfres

28. Warsame Id A, Fuje Id M, Id FC, Blanchet Id K, Palmer J. Evaluating COVID-19 decision-making in a humanitarian setting: The case study of Somalia. Mahendradhata Y, editor. PLOS Glob Public Heal [Internet]. 2022 Mar 16 [cited 2022 Mar 25];2(3):e0000192. Available from: https://journals.plos.org/globalpublichealth/article?id=10.1371/journal.pgph.0000192

29. Schippers MC, Rus DC. Optimizing Decision-Making Processes in Times of COVID-19: Using Reflexivity to Counteract Information-Processing Failures. Front Psychol. 2021 Jun 22;12:2261.

30. Sanderson C. Analytical models for decision-making. Open Univ Press. 2006;

31. Berger L, Berger N, Bosetti V, Gilboa I, Hansen LP, Jarvis C, et al. Rational policymaking during a pandemic. Proc Natl Acad Sci U S A [Internet]. 2021 Jan 26 [cited 2021 Nov 29];118(4). Available from: https://www.pnas.org/content/118/4/e2012704118

32. Morgan O. How decision makers can use quantitative approaches to guide outbreak responses [Internet]. Vol. 374, Philosophical Transactions of the Royal Society B: Biological Sciences. Royal Society Publishing; 2019 [cited 2020 Aug 11]. Available from: /pmc/articles/PMC6558558/?report=abstract

33. Gouglas D, Marsh K. Prioritizing investments in new vaccines against epidemic infectious diseases: A multi-criteria decision analysis. J Multi-Criteria Decis Anal [Internet]. 2019 May 1 [cited 2022 Feb 11];26(3–4):153–63. Available from: https://onlinelibrary.wiley.com/doi/full/10.1002/mcda.1683

34. Storeng KT, Abimbola S, Balabanova D, McCoy D, Ridde V, Filippi V, et al. Action to protect the independence and integrity of global health research. BMJ Glob Heal [Internet]. 2019 Jun 1 [cited 2022 May 14];4(3):e001746. Available from: https://gh.bmj.com/content/4/3/e001746

35. Sager F, Plegger L. Evaluation and Independence Existing evaluation policies and new approaches. 2016.

36. OECD. PRINCIPLES FOR EVALUATION OF DEVELOPMENT ASSISTANCE. Paris; 1991.

37. Evaluation W office for. Decentralized evaluation for evidence-based decision making WFP Office of Evaluation Decentralized Evaluation Quality Assurance System (DEQAS) Technical Note Independence and Impartiality.

38. Palmer J. How is community engagement normally done? | COVID-19 Hygiene Hub Resources [Internet]. 2022 [cited 2022 May 14]. Available from: https://resources.hygienehub.info/en/articles/4028678-how-is-community-engagement-normally-done

For peer review only

Figure Legend

Figure 1 Structure of the audit tool

Figure 2 Data collection timeline of evaluation

Figure 3 Extract of decision scorecards where accountability was rated low

For peer review only

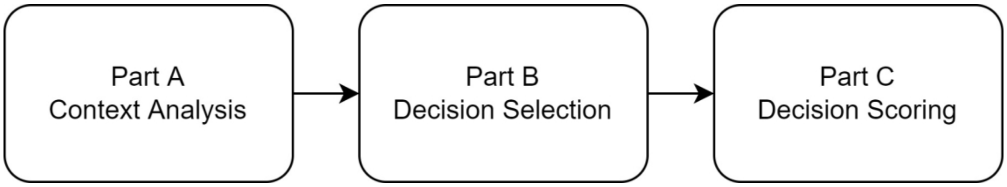

Figure 1 Structure of the audit tool

636x114mm (72 x 72 DPI)

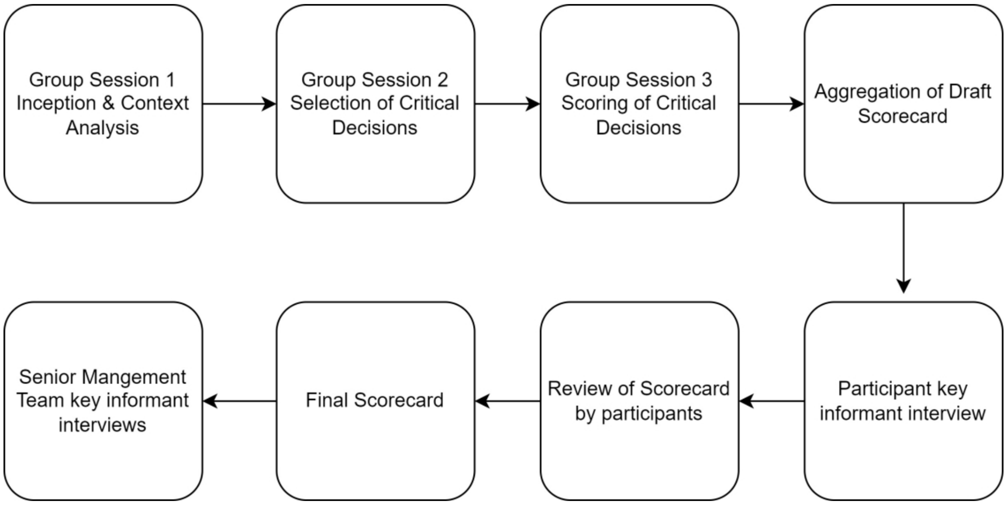

Figure 2 Data collection timeline of evaluation

855x424mm (72 x 72 DPI)

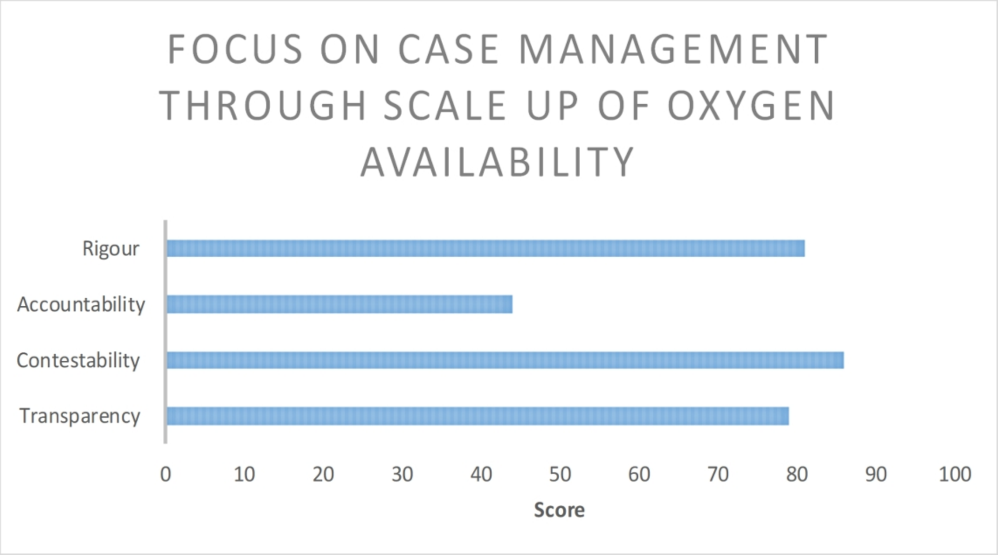

Figure 3 Extract of decision scorecards where accountability was rated low

871x484mm (59 x 59 DPI)

Evaluating the quality of epidemic decision-making in epidemics & humanitarian crises

Standard Operating Procedures (SOP)

For peer review only

Version 1.0
June 30th, 2021

Abdihamid Warsame
Infectious Disease Epidemiology
London School of Hygiene & Tropical Medicine
Abdihamid.warsame@lshtm.ac.uk

Contents

Evaluating epidemic decision-making in humanitarian crises 1
Standard Operating Procedures (SOP) 1
Abbreviations 4
Introduction 5
Rationale 6
Definitions 6
Target audience 6
Evaluation team composition 6
Timeline 7
Section 1- Context analysis 8
1.1 Purpose 8
1.2 Roles and responsibilities 8
1.3 Procedures 8
1.4 Outputs 8
1.5 Associated Documents 8
Section 2- Decision Selection 8
2.1 Purpose 9
2.2 Roles and Responsibilities 9
2.3 Procedures 9
2.4 Outputs 9
2.5 Associated Documents 9
Section 3- Decision Scoring 10
3.1 Purpose 10
3.2 Roles and responsibilities 10
3.3 Procedures 10
3.4 Outputs 10
3.5 Associated Documents 10
Section 4- Dissemination/ Review 11
4.1 Purpose 11
4.2 Roles and responsibilities 11
4.3 Procedures 11
4.4 Outputs 11
4.5 Associated Documents 11
Reference 12

For peer review only - http://bmjopen.bmj.com/site/about/guidelines.xhtml

Annex 13

Document Checklist 13

Decision-making evaluation tool .... **Error! Bookmark not defined.**

Background .... **Error! Bookmark not defined.**

Part A- Context analysis .... **Error! Bookmark not defined.**

Part B- Selection of critical decision points .... **Error! Bookmark not defined.**

Key informant interview guide .... **Error! Bookmark not defined.**

Flow chart .... **Error! Bookmark not defined.**

For peer review only

For peer review only - http://bmjopen.bmj.com/site/about/guidelines.xhtml

Abbreviations

CO- Country Office

COVID-19- Coronavirus disease 2019

RO- Regional Office

HQ- headquarters

Sitrep- Situation report

PHSA- Public Health Situation Analysis

RRA- Rapid risk assessment

SMT- Senior Management Team

KII- Key Informant Interview

FGD- Focus Group Discussion

For peer review only

For peer review only - http://bmjopen.bmj.com/site/about/guidelines.xhtml

Introduction

While epidemics of infectious disease continue to pose a considerable threat to populations in low-income and humanitarian settings, the evaluation of these epidemic responses are limited and heterogeneous [1]. Populations in need of humanitarian assistance or living within low income settings continue to grow[2] and the ongoing COVID-19 pandemic has further exacerbated the impact of these crises[3]. Decision-making in the context of an ongoing epidemic is particularly challenging[13,14]. Previous research has identified a limited number of decision-making factors contributing to poor response [15–17]. However, these findings need further study in order to confirm their relevance to a wider array of contexts experiencing ongoing epidemics

Such settings not only must deal with the epidemic directly and its indirect impacts but must also contend with a host of urgent competing emergencies. As such decision-makers are under heavier strain than those in better resourced and stable environments and require adequate support. COVID-19 is unlikely to be the last epidemic faced by decision-makers in such settings and therefore evaluations such as this are especially critical to strengthening future preparedness and improving response.

In decision-making research, there are two common approaches: process and evaluation. Process oriented approach describes how decisions are actually made within a specific context whereas analytical oriented is more prescriptive and frames decision-making as a problem-solving approach intended to lead to the best outcome. In short the former looks at whether a decision is made correctly while the latter considers whether the correct decision is made. This evaluation is predicated on the former approach.

For peer review only - http://bmjopen.bmj.com/site/about/guidelines.xhtml

Rationale

- This SOP is intended to evaluate the process of epidemic decision-making rather than the impact of any specific decision. Improved decision-making practises can improve outcomes, timeliness and accountability in epidemic response in humanitarian settings.

- In order to contribute to better humanitarian performance, the UKRI awarded a consortium led by the London School of Hygiene & Tropical Medicine to undertake research and produce evidence to support humanitarian responders. This SOP includes stepwise actions required to be undertaken by evaluators as well as the necessary timeframes, roles and responsibilities and resources required.

Definitions

Decision making: choosing between number of options/course of action or a process of problem solving in a specific context (Campbell 2018)

Evaluation- assessment, as systematic and impartial as possible of an activity, project, programme, strategy, policy, theme, sector, operational area or institutional performance.

Target audience

- This standard operating procedure is intended for the evaluation lead within the humanitarian organization. However, it can also be utilized by third party monitors as part of an overarching review of a humanitarian organizations epidemic response.

Evaluation team composition

Although evaluation should ideally be done by independent third party, staff members in the evaluated organization have a significant role to play in the planning, design, implementation and use of this evaluation.

For peer review only

For peer review only - http://bmjopen.bmj.com/site/about/guidelines.xhtml

Timeline

- The evaluation should ideally take place after the first X weeks since the onset of the epidemic. It may also take place immediately after the conclusion of the epidemic

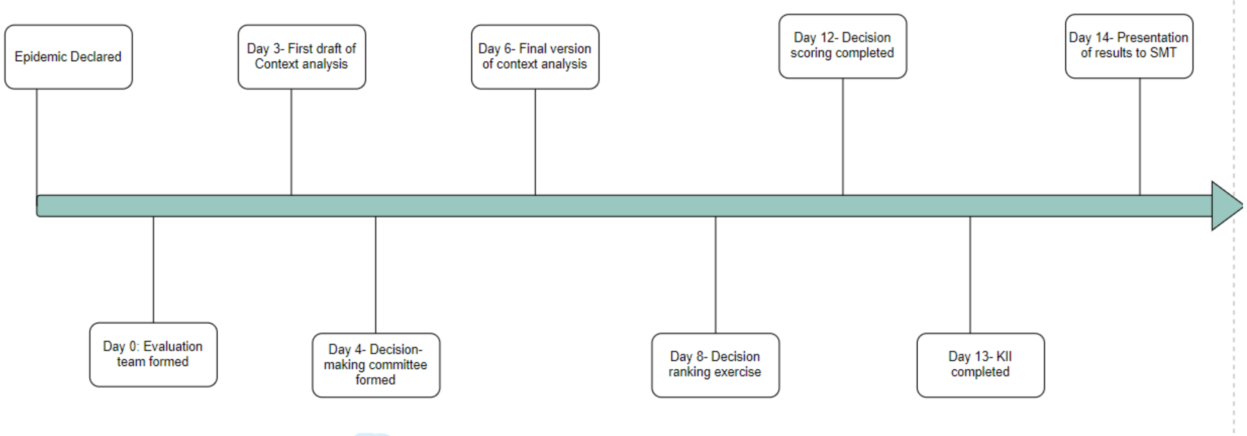

r peer review only

Section 1- Context analysis

1.1 Purpose

- The purpose of the context analysis is to provide a common base from which to understand the epidemic setting and the actions that have been taken.

1.2 Roles and responsibilities

- The evaluation team lead will lead the exercise and convene the evaluation team to undertake the context analysis.
- The evaluation team will identify principle decision-makers to form decision-making committee

1.3 Procedures

- The evaluation team will undertake a secondary data review in order to produce the first draft of the context analysis
- The review should gather all relevant documentation including:
  - Previous Public Health Situation Analyses (PHSA)
  - Rapid risk assessments (RRA)
  - Response planning documentation
  - Organizational organograms
  - Sitreps
  - Health bulletins
  - Communication products
  - Meeting minutes
- The evaluation team will also identify key internal decision-makers involved in the particular epidemic response who have had significant input in the planning, implementation or evaluation of the epidemic response. These maybe situated within the country office or regional or HQ levels. These may include technical experts, organizational leaders and others
- These decision-makers will be invited to form a committee to contribute to the remaining sections of this evaluation
- The evaluation team lead will circulate the context analysis draft and solicit feedback in a timely manner from the decision-makers
- The evaluation team will consolidate context analysis feedback into a final version

1.4 Outputs

- Consolidated and agreed upon context analysis which summarizes epidemiological description of the epidemic, health resources and services available to the population, health system performance, organizational resources expended in the epidemic response to date, as well as challenges faced in the response

1.5 Associated Documents

See Annex for document checklist, participant list and section 1 of the tool

For peer review only - http://bmjopen.bmj.com/site/about/guidelines.xhtml

Section 2- Decision Selection

2.1 Purpose

The purpose of this section is to produce a unified list of key decisions to evaluate as prelude to evaluate decision-making in the epidemic response.

2.2 Roles and Responsibilities

- The evaluation team lead is responsible for sharing key documentation and producing an initial listing of key decisions
- The decision-making committee is responsible for reviewing, contributing and evaluating the criticality of decisions

2.3 Procedures

- The evaluation team lead will circulate the context analysis and introduce the characteristics for selecting key decisions to the evaluation team.
- In conjunction with the evaluation team, he/she will produce up to 5 key decisions for initial consideration by key decision-making committee identified through the context analysis
- The key decision-makers will review the proposed decisions
- Decision-makers may contribute further decisions for consideration
- Decision-makers will score each of the proposed decisions based on the scoring criteria
- Evaluation team will summarize (take the mean) the score for each decision and select the top 5 decisions using the following formula

2.4 Outputs

- Unified list of no more than 5 key decisions to be further assessed in section 3.

2.5 Associated Documents

See annex for section 2 of the tool

For peer review only

For peer review only - http://bmjopen.bmj.com/site/about/guidelines.xhtml

Section 3- Decision Scoring

3.1 Purpose

The purpose of this section is to ensure that selected critical decisions are evaluated consistently across the assessment criteria and to produce a summary score for the overall epidemic decision-making.

3.2 Roles and responsibilities

- The evaluation team lead is responsible for explaining the evaluation dimensions and criteria
- The evaluation team is responsible for assessing each decision on the criteria

3.3 Procedures

- For each of the selected decisions (maximum 5) the evaluation team will assess whether the necessary decision-making criteria has been met
- They will compile evidence through review of documentation and score the decision on a scale of 1 (no evidence/not met) to 5 (criteria fully met)
- Where there is little or no evidence of a criteria being met, the evaluation team may revert to decision-making committee to provide evidence
- The decision-making committee members are responsible for providing evidence for any specific criteria as requested by the evaluation team
- The evaluation team lead will review the final score for each decision as well compute an overall score for the exercise

3.4 Outputs

- An agreed upon set of scores for each of the critical decisions as well as an overall score

3.5 Associated Documents

Refer to annex for section3 of the tool

For peer review only - http://bmjopen.bmj.com/site/about/guidelines.xhtml

Section 4- Dissemination/ Review

4.1 Purpose

- The purpose of this section is to review and disseminate the findings of the evaluation in order to improve response processes

4.2 Roles and responsibilities

- The evaluation team lead is responsible for compiling the views of organizational members on the overall evaluation process
- The evaluation team lead is responsible for presenting the findings of the evaluation back to the organization
- The organizational leadership is responsible for working with relevant staff members in implementing improvements

4.3 Procedures

- The evaluation team lead should convene staff who have participated in the evaluation and implement focus group discussion (FGD) or key informant interviews(KII) to elicit feedback on the process
- The evaluation team should present the results of both the exercise and participant views to organizational leadership/ Senior Management Team (SMT)in a brief
- The SMT should implement recommendations derived from the exercise

4.4 Outputs

- Identification of decision rated as strong
- Recommendations of decision making dimensions that require strengthening
- Feedback on the utility and feasibility of evaluation
- Identification of areas of the evaluation process that require improvement

4.5 Associated Documents

- See annex for focus group guide and key informant interview guide

For peer review only - http://bmjopen.bmj.com/site/about/guidelines.xhtml

Reference

https://jhumanitarianaction.springeropen.com/articles/10.1186/s41018-020-00068-2#Fn3
https://www.who.int/hac/techguidance/tools/standard_operating_procedures_african_region_en_2014.pdf

https://healthcluster.who.int/docs/librariesprovider16/meeting-reports/updated-phsa-long-form-template-english-june-2019.docx?sfvrsn=81cd6a71_3

For peer review only

For peer review only - http://bmjopen.bmj.com/site/about/guidelines.xhtml

Annex

Document Checklist

No.	Document Type	Details (e.g.)
1	Organogram	*CO organogram March 2021*
2	Meeting Minutes	*Weekly minutes (may 2021-June 2021)*
3		
4		
5		

Evaluation Participants

Evaluation Team	Name (e.g.)	Role
1	*John Doe*	*Team lead*
2	*Pers Indi*	*M&E officer*
3		
Decision-making committee	Name	Role
1	*Fulan*	*Incident manager*
2	*Jama Yulo*	*Operations officer*
3	*Qofa Kale*	*Technical officer*

For peer review only

For peer review only - http://bmjopen.bmj.com/site/about/guidelines.xhtml

Decision Making Evaluation Instrument

Organization:

Date:

Outbreak:

Part A- Summary of Response

(i) Describe briefly the historical, geographic and health context in which the outbreak is occurring *(maximum 250 words).*

(ii) Briefly describe the course of the epidemic from detection up the present focusing on epidemiology, risk factors and effected populations *(maximum 250 words).*

(iii) Describe what actions your organization has taken in response to the epidemic *(maximum 300 words).*

For peer review only

LONDON SCHOOL of HYGIENE &TROPICAL MEDICINE

Part B- Selection of critical decision points

Guide

Considering the following guiding Characteristics, propose 3 -5 critical decision in the response to date. Justify your selection with specific with reference to the characteristics as well as specific examples. A decision does not need to meet all of the following characteristics to be considered critical. These can include decision on beginning, ending or scaling a response, deciding on response options, deciding on target population, as well as deciding on modality of response.

Characteristics of critical decision

I. Consequential- critical decision is consequential and shapes the response to a significant degree
II. Reversibility- a critical decision can be difficult to overturn or reverse at least in the short term)
III. Strategic- entails shift in terms of action taken, resources committed or precedent set
IV. Uncertainty –Wide range of uncertainty/ complex array of options
V. Reputionally Risky- Entails a high level of organisational reputational risk

Decision 1

Description
Describe the decision

Rationale
Justify your proposed decision by selecting the relevant Characteristics

☐ Characteristic 1

☐ Characteristic 2

☐ Characteristic 3

☐ Characteristic 4

☐ Characteristic 5

Narrative
Describe any other reason for considering this decision critical (maximum 200 words)

Decision 2

For peer review only - http://bmjopen.bmj.com/site/about/guidelines.xhtml

Description

Describe the decision

Rationale

Justify your proposed decision by selecting the relevant Characteristics

☐ Characteristic 1

☐ Characteristic 2

☐ Characteristic 3

☐ Characteristic 4

☐ Characteristic 5

Narrative

Describe any other reason for considering this decision critical (maximum 200 words)

Decision 3

Description

Describe the decision

Rationale

Justify your proposed decision by selecting the relevant Characteristics

☐ Characteristic 1

☐ Characteristic 2

☐ Characteristic 3

☐ Characteristic 4

☐ Characteristic 5

Narrative

Describe any other reason for considering this decision critical (maximum 200 words)

For peer review only - http://bmjopen.bmj.com/site/about/guidelines.xhtml

For peer review only

For peer review only - http://bmjopen.bmj.com/site/about/guidelines.xhtml

LONDON SCHOOL of HYGIENE &TROPICAL MEDICINE

Part C-Decision-making criteria

For each selected decision in Part B, complete the following scoring exercise, keeping in mind to provide the necessary evidence.

Decision: __*e.g. Decision to conduct mobile vaccinations in region Y in May 2020*

Transparency

To what degree were the following criteria met?

Criteria	Fulfilment of criteria<br>*To what extent was the criteria fulfilled in this particular decision*	Evidence of fulfilment<br>*Provide reference to specific sources to support your assessment*
Inclusivity[1]	5- Very Strong evidence	*e.g. Refer to meeting minutes March 24, 2020 as well as response plan April 2020. These show presence of diverse stakeholders in this decision from all departments as well as MoH*
Usage of explicit decision-making criteria[2]	Choose an item.	
Following clear process or method[3]	Choose an item.	
Usage of mechanism to publicise rationale [4]	Choose an item.	

Guide

1. Consider who was involved. Was there heterogeneity in ranks and roles?

2. Were the goals and objectives clearly pre-specified or were decisions rationalized after the fact?

For peer review only - http://bmjopen.bmj.com/site/about/guidelines.xhtml

3. Were there documented priority setting process and/or use of priority setting framework? Was there a decision-tree

4. Was there clear documentation on decisions reached?

For each selected decision in Part B, complete the following scoring exercise, keeping in mind to provide the necessary evidence.

Decision: ____

Contestability

To what degree was the following criteria met?

Criteria	Fulfilment of criteria<br>*To what extent was the criteria fulfilled in this particular decision*	Evidence of fulfilment<br>*Provide reference to specific sources to support your assessment*
Opportunity for revision[5]	Choose an item.	
Was the decision devolved?[6]	Choose an item.	

Accountability

To what degree was the following criteria met?

Criteria	Fulfilment of criteria<br>*To what extent was the criteria fulfilled in this particular decision*	Evidence of fulfilment<br>*Provide reference to specific sources to support your assessment*
Engagement with effected communities[7]	Choose an item.	

For peer review only - http://bmjopen.bmj.com/site/about/guidelines.xhtml

Rigour

Criteria	Fulfilment of criteria<br>*To what extent was the criteria fulfilled in this particular decision*	Evidence of fulfilment<br>*Provide reference to specific sources to support your assessment*
Explicit outcome[8]	Choose an item.	
Feasible outcome[9]	Choose an item.	
Strengthen healthcare system[10]	Choose an item.	
Evidence based [11]	Choose an item.	

Guide

5. Was there any scope to revise and overturn a decision? How open was the discussion? Were alternatives debated? What were they? Was consensus achieved and if so, how was this reached?

6. What role did respondents in closest proximity to the epidemic (e.g. subnational level) or local technical experts play? What role did organizational leadership play? Who was the most junior decision-maker? Who was the most senior?

7. To what degree were the effected communities involved in the response? Who was involved from the effected community? Were they informed of the response activities? Did this notice make any difference for those notified?

8. Were intended outcomes of the decision clearly articulated? Were targets set? What were they?

9. Were alternatives debated? What were they?

10. Was there evidence that this decision was in-line with wider strategy? What pillar of the health system were targeted?

For peer review only - http://bmjopen.bmj.com/site/about/guidelines.xhtml

11. Was "Strong public health rationale and solid scientific information provided to justify health measures?" What evidence was utilized? What evidence was missing?

Part D- Summary Decision-making Score

Scoring formula

For each assessed decision, utilize the formulae below to populate the table

Transparency score = average of criteria 1-4

Contestability= average of criteria 5-6

Accountability= score of criteria 7

Rigour= average of criteria 8-11

Dimension	Average	Score
Transparency		
Contestability		
Rigour		
Accountability		

For peer review only

For peer review only - http://bmjopen.bmj.com/site/about/guidelines.xhtml

Recommendations

Based on the results above, briefly describe what specific steps you recommend in order to improve the decision-making process in this epidemic *(maximum 300 words).*

For peer review only - http://bmjopen.bmj.com/site/about/guidelines.xhtml

For peer review only

For peer review only - http://bmjopen.bmj.com/site/about/guidelines.xhtml

For peer review only

For peer review only - http://bmjopen.bmj.com/site/about/guidelines.xhtml

Decision-making Scorecard

WHO Somalia's COVID-19 response

Evaluation study

Nov 22nd- December 8th 2021

Abdihamid Warsame

Faculty of Epidemiology and Population health

London School of Hygiene and Tropical Medicine

For peer review only

Critical Decisions

Participants were advised to select important decisions for critical evaluation based on the following criteria on what constitutes a critical decision.

Characteristics

A critical decision is one which has one or more of the following characteristics:

I. Consequentiality-: A critical decision is consequential and shapes the response to a significant degree
II. Reversibility-: A critical decision can be difficult to overturn or reverse at least in the short term
III. Strategic: A critical decision entails a significant shift in terms of action taken, resources committed or precedent set
IV. Uncertainty: A critical decision entails a wide range of uncertainty or complex array of options
V. Reputational Risk: A critical decision entails a high level of organisational reputational risk

Decision	Criteria for Selection
i. Formation of Rapid Response Teams	☒ Characteristic 1<br>☐ Characteristic 2<br>☒ Characteristic 3<br>☒ Characteristic 4<br>☒ Characteristic 5
ii. Establishment of 3 strategic PCR labs	☒ Characteristic 1<br>☒ Characteristic 2<br>☒ Characteristic 3<br>☒ Characteristic 4<br>☒ Characteristic 5
iii. Launch of Incident Management System	☒ Characteristic 1<br>☐ Characteristic 2<br>☒ Characteristic 3<br>☒ Characteristic 4<br>☒ Characteristic 5
iv. Focus on case management through scale up of Oxygen	☒ Characteristic 1<br>☐ Characteristic 2<br>☒ Characteristic 3<br>☒ Characteristic 4<br>☒ Characteristic 5

For peer review only

Summary Decision Scorecard

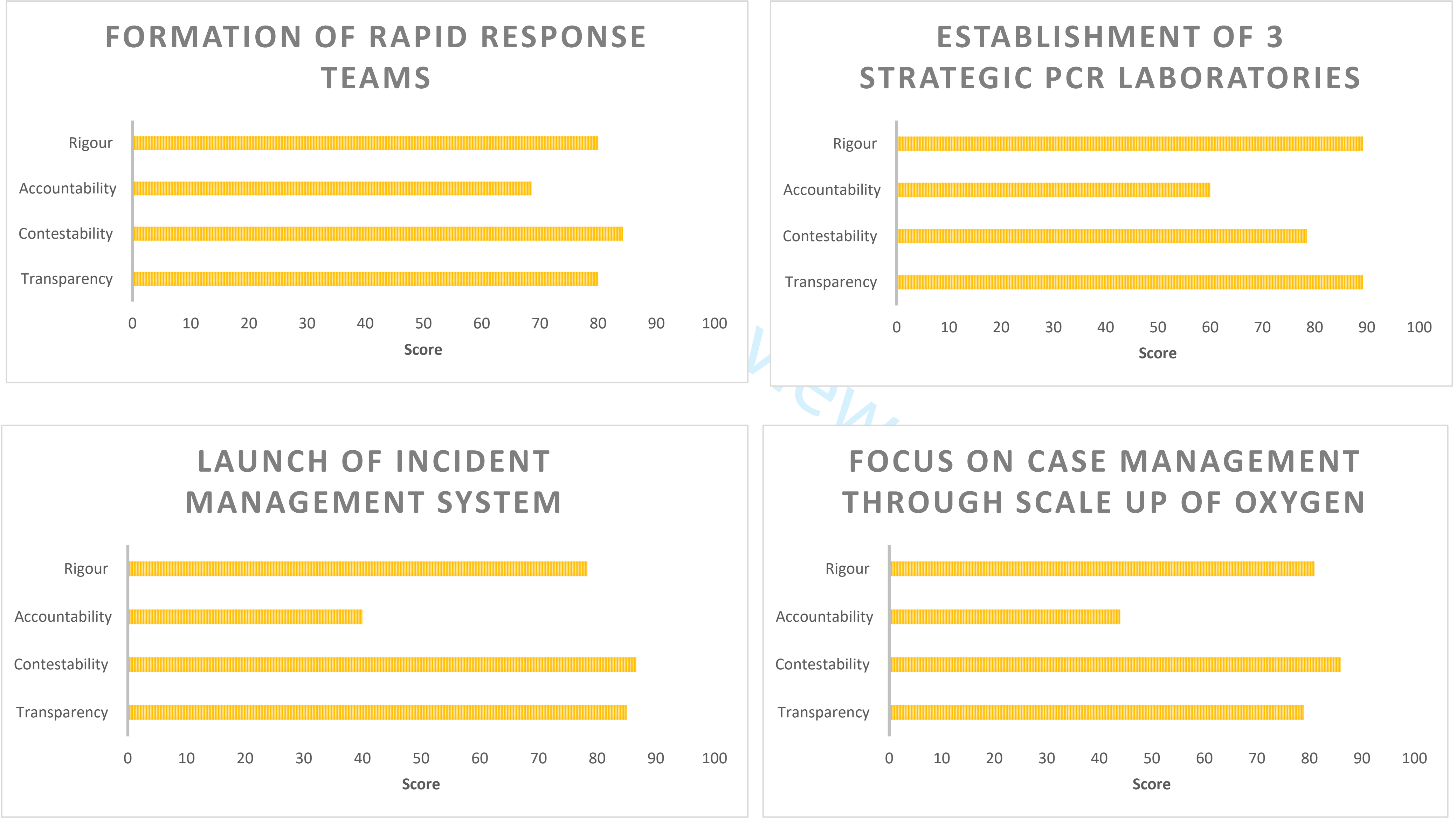

Detailed Scores & Interpretation

i. Formation of Rapid Response Teams

The Rapid Response Teams (RRT) and Community Health Workers (CHW) were recruited early in the COVID-19 response in order to strengthen and expand the surveillance capacity in high priority districts. A decision was undertaken to train and equip members of the RRTs and CHWs for case detection, notification, testing and contact tracing.

Inclusivity 86%	Decision criteria 74%	Process 77%	Public rationale 83%

Participants scored the transparency of this decision as high citing existing documentation such as response plan, IMST meeting minutes and proposal documents. They stated that there was strong evidence of inclusive decision-making as the decision included WHO staff from all programs as well as program managers. They also stated that some of the decision-makers were assigned to liaise/coordinate with the stakeholders including MoH and other partners and discuss the decision with them and publicise the rationale. They also noted following clear method for arriving at this decision including utilizing risk assessments. Participants referenced the usage of COVID-19 transmission classification for Somalia drawn from the interim guidelines by WHO for considerations for implementing and adjusting public health and social measures in the context of COVID-19 as providing clear criteria for this decision.

Devolution 74%	Revision 94%	Community engagement 69%	Explicit outcome 77%

Contestability was the highest scored dimension. Participants noted there were sufficient opportunities to revise the decision and cited the inter-action review of November 2020 as evidence. Additionally, the decision was discussed in various IMST meeting with document shared and commented, feedbacks incorporated into the final decision. The MoH was also said to be involved in the selection of RRTs and CHWs, implementation and modification of the structure and role and responsibilities. They also noted that revision of this decision resulted in expansion of the RRT mechanism to further district. Participants also noted that the IMST structure allowed for devolution in the decision-making in which decision-making was shared amongst senior decision-makers (country level IMST team members under the leadership of the WR) and junior decision makers (state level and district level staff)

In terms of accountability, this was scored lower as there was difference of scoring amongst participants as to the degree of engagement with the effected communities. Some participants stated that there was strong engagement with communities in implementing the RRT strategy but limited engagement in the decision to use this mechanism.

Feasibility 88%	Strengthen Health system 74%	Evidence based 77%

Participants noted this decision was taken with a high degree of rigour. In particular, they noted that strengthening healthcare system was among the objectives. Furthermore, they noted that the outcomes of this decision were explicitly articulated and include bridging the gap between health facilities and communities. They also noted that feasibility criteria were considered to be high due to staffing the RRT via the existing network of CHWs. Lastly they noted that targets were set and tracked through an interactive dashboard.

ii. Establishment of 3 strategic PCR labs

At the outset of the pandemic in Somalia, there did not exist national capacity to test samples for COVID-19. Responders relied in the first weeks, upon sending samples for testing abroad which were found to be time consuming and expensive. The lack of national labs to undertake this critical function was found to be a gap which required urgent attention. As a result, the decision was taken to establish three strategic PCR capable laboratories in Mogadishu, Garowe and Hargeisa.

Inclusivity 91%	Decision criteria 89%	Process 89%	Public rationale 89%

The transparency score is comprised of 4 criteria. In terms of inclusivity, participants scored this decision highly. terms of transparency. The decision was taken in conjunction with the MoH and UN partners. The rationale behind this decision was said to have been shared in internal coordination meetings as well as external coordination meetings with partners. A number of documents were cited as evidence including WHO Somalia Technical Program Update Quarterly, IMST meeting minutes, CERF Project Action Plan and WCO FCDO report.

Devolution 65%	Revision 91%	Community engagement 60%	Explicit outcome 91%

In terms of contestability, this decision was reviewed in the initial proposal development meeting with the MoH in which there was opportunity for revision. Additionally, participants stated that a review of the decision prompted further procurement of PCR machines and scaling up of PCR testing capacity to remaining states.

Participants expressed mixed scoring on the accountability dimension. Some stated that there was high accountability through interaction with state level MoH while others expressed that there was limited/no consultation with communities in these 3 cities regarding the establishment of PCR capacity in these labs. Some participants stated this criterion was inapplicable as the labs were pre-existing and upgrading did not require further engagement.

Feasibility 91%	Strengthen Health system 89%	Evidence based 89%

Participants scored this decision highly in terms of scientific rigour. They stated that the usage of PCR is global standard to detect COVID-19. Additionally, they stated the decision was built on evidence extracted from a previous 2019 influenza testing lab assessment which provided rationale and process for the decision to set up these labs for COVID-19 testing. Participants also cited the National Action Plan for Health Security as providing evidence for this decision. The decision was also found to have satisfied the strengthened the health system criteria as the establishment of these 3 labs allowed for testing beyond COVID-19.

For peer review only

iii. Launch of Incident Management System

As part of efforts to enhance the effectiveness of the Health Emergencies response and in line with the Emergency response framework, WHO Somalia launched the Incident Management Support Team to coordinate and guide the response to the COVID-19 pandemic within Somalia.

Inclusivity 90%	Decision criteria 83%	Process 80%	Public rationale 87%

Participants scored the inclusivity of this decision as high as they stated this decision was taken after a series of consultation with staff led by the WR. These deliberations were captured in meeting minutes and which detailed the process and rationale. The final decision was communicated by WR to all staff through an office memo. Participants also noted the engagement with the MoH in the formation of the IMST system as evidence of inclusivity.

Devolution 80%	Revision 93%	Community engagement 40%	Explicit outcome 77%

In terms of revision, participants noted that there was some opportunity which resulted in the change of incident managers but not in the structure itself. With regards to accountability, participants noted limited to no engagement with effected communities. However other participants stated that parallel state and district-level IMST did engage with the local community. While these lower level IMST did not feed into the formation of the national IMST, they maintain a certain level autonomy.

Feasibility 83%	Strengthen Health system 80%	Evidence based 73%

Participants cited a number of routine information products as evidence of decision outcomes and feasibility considerations. They cited COVID-19 Response plan and action trackers as evidence for the articulation of clear outcomes and evidence based targets. They also cited this response plan which articulated heath system strengthening as an explicit goal and an important pillar in the IMST approach. Participants also cited the WHO Global guidance on strategic response to C19 as providing evidence to support this decision.

iv. Focus on case management through scale up of Oxygen

Due to the presentation of symptoms and the aetiology of the disease as well as gaps in case management capacity, WHO Somalia undertook concerted effort to scale up oxygen therapy across the country in order to improve outcomes.

Inclusivity 76%	Decision criteria 76%	Process 76%	Public rationale 88%

The decision-making was scored as somewhat inclusive by participants. They cited the IMST meeting minutes in June 2020 as evidence as well as the COVID-19 response plan which detailed the discussion and decision criteria. They noted close deliberation with the case management focal persons within the MoH. Participants also noted that the rationale was communicated clearly through specific information products and web stories on WHO and other websites.

For peer review only

Devolution 84%	Revision 88%	Community engagement 44%	Explicit outcome 76%

Participants noted close discussion with federal member states on the modality of scale up as evidence of devolved decision-making. Participants mentioned that there were several opportunities to revise this decision in which the need for oxygen provision was reaffirmed but the modality was revised. They noted that the initial decision was for WHO to support procurement of oxygen locally. This changed to procurement of cylinders and ventilators and eventually to supporting the establishment of oxygen plants. Participants mentioned limited evidence of community engagement in the decision itself but significant engagement through health messaging after the fact. They cited dashboards as containing evidence of clear outcomes and targets that underlay this decision.

Feasibility 80%	Strengthen Health system 84%	Evidence based 84%

Participants noted that this decision supported strengthening of the health system by fortifying performance of isolation facilities and optimizing case management. They also cited later documents as providing evidence that underlay this decision such as the technical update on survival among hospitalized COVID-19 patients.

Recommendations

A number of recommendations were put forward by participants in order to strengthen the decision-making process within the organisation. They include the following:

- Improve documentation on decision-making deliberations as participants were aware of many discussions that were not reflected in official internal or external records.
- To further strengthen decision mechanisms by clearly outline who is involved, their responsibilities and the mechanisms by which they arrived at the decision.
- Strengthen engagement with affected communities in addition to government officials *during* the decision-making process rather than solely communicating finalised decisions. This will strengthen accountability.
- Consider sustainability as a potential criterion for good decision-making
- Repeat this decision-making exercise on a more routine basis by adopting it as a component of internal evaluation

For peer review only

Annex

Score Criteria

Dimension	Criteria	Description
**Transparency**	Inclusivity	The extent to which the process was inclusive, reflected in heterogeneity in rank and roles amongst decision makers involved.
	Use of explicit decision-making criteria	The extent to which the goals and objectives of the decision were clearly pre-specified. The absence of post-decision rationalization.
	Following clear process or method	The extent to which a priority setting process was in place, reflected in demonstrated use of priority setting frameworks, decision trees or other mechanism.
	Use of mechanism to publicise rationale	The extent to which clear documentation on the decision exists as well as the method used to communicate decisions.
**Contestability**	Opportunity for revision	The extent to which there existed scope to revise and overturn a decision including the debating of alternatives and description of how consensus was reached.
	Was the decision devolved?	The degree to which participants in closest proximity to the epidemic (e.g. subnational level) or local technical experts participate in the decision, including consideration of rank.
**Accountability**	Engagement with affected communities	The degree to which affected communities were involved in the response decision-making including at a minimum whether they were informed of the response activities and what effect this notification had on the communities.
**Rigour**	Explicit outcome	The extent to which intended outcomes of the decision were clearly articulated, including through setting of targets.
	Feasible outcome	The extent to which feasibility was considered in decision-making including debating of alternatives.
	Strengthens healthcare system	The extent to which the decision was in-line with wider strategy including the strengthening of the health system
	Evidence based	The extent to which the decision was based on strong public health rationale and robust scientific information.

For peer review only

Decision-making audit scorecard

CARE Somalia’s COVID-19 response

Evaluation study

November 8th -Nov 22nd 2021

Abdihamid Warsame
Faculty of Epidemiology and Population health
London School of Hygiene and Tropical Medicine

21 January 2022

For peer review only

Background

On the 30th of January 2020, the World Health Organization (WHO) declared the emergence of SARS-CoV-2 corona virus as a Public Health Emergency of International Concern (PHEIC) because of evident human-to-human transmission and renamed it COVID-19. On the 16th of March 2020, Somalia reported its 1st COVID- 19 confirmed case, a Somali national with travel history to China. As of the 20th of January 2022, Somalia had 24,261 confirmed cases of COVID-19 with 1335 deaths. The Federal Ministry of Health launched the National COVID-19 Preparedness & Response Plan on the 26th March 2020 with a budget of USD 57 million and estimated caseload of 6,000 confirmed cases.

Somalia's capacity to prevent, detect, and respond to any global health security threat scored six out of 100 on the 2016 Health Emergency Preparedness Index. Poor urban communities, internally displaced persons(IDPs), the elderly, households of minority clans, riverine communities, women and child headed households, and children are most at risk due to social exclusion and decreased accessibility to health services, sanitation and water.

The COVID-19 pandemic weakened Somalia's already fragile health system that has been negatively impacted by protracted conflict, cyclic drought, and locust infestation. The Somali health system has not been able to cope adequately with the demands of a COVID-19 response with poor and few testing facilities, low capacity of health care workers and poor funding. Social cultural practices, rumours, misconceptions, poor hygiene practices and communal caring for the sick in homes and communities, have additionally amplified the spread of the virus.

Methods

The evaluation occurred in Garowe, Somalia from 8- 18th of November 2021. A total of 8 participants from various units within CARE Somalia took place in this evaluation.

The evaluation was undertaken following the standard operating procedures (SOP) developed by the LSHTM research team (See annex) and with the appointment of a CARE focal point. The CARE focal point gathered the necessary background documentation as well as supported the formation of the decision-making committee through recruitment of evaluation participants from the organization. The focal point completed the first draft of the contextual analysis as well as consolidated input from the other participants. He was also instrumental in facilitating the group work sessions.

For peer review only - http://bmjopen.bmj.com/site/about/guidelines.xhtml

Critical Decisions

Participants were advised to select important decisions for critical evaluation based on the following criteria on what constitutes a critical decision.

Characteristics

A critical decision is one has one or more of the following characteristics:

I. Consequentiality-: A critical decision is consequential and shapes the response to a significant degree
II. Reversibility-: A critical decision can be difficult to overturn or reverse at least in the short term
III. Strategic: A critical decision entails a significant shift in terms of action taken, resources committed or precedent set
IV. Uncertainty: A critical decision entails a wide range of uncertainty or complex array of options
V. Reputational Risk: A critical decision entails a high level of organisational reputational risk

Decision	Criteria for Selection
i. Adaptation of nutrition programming to COVID-19	☐ Characteristic 1<br>☒ Characteristic 2<br>☒ Characteristic 3<br>☐ Characteristic 4<br>☐ Characteristic 5
ii. Closure of CARE offices and restriction of staff movement	☒ Characteristic 1<br>☐ Characteristic 2<br>☒ Characteristic 3<br>☐ Characteristic 4<br>☒ Characteristic 5
iii. Implementation of COVID-19 project in Sool and Sanaag	☒ Characteristic 1<br>☒ Characteristic 2<br>☒ Characteristic 3<br>☒ Characteristic 4<br>☐ Characteristic 5

For peer review only - http://bmjopen.bmj.com/site/about/guidelines.xhtml

Summary Decision Scorecard

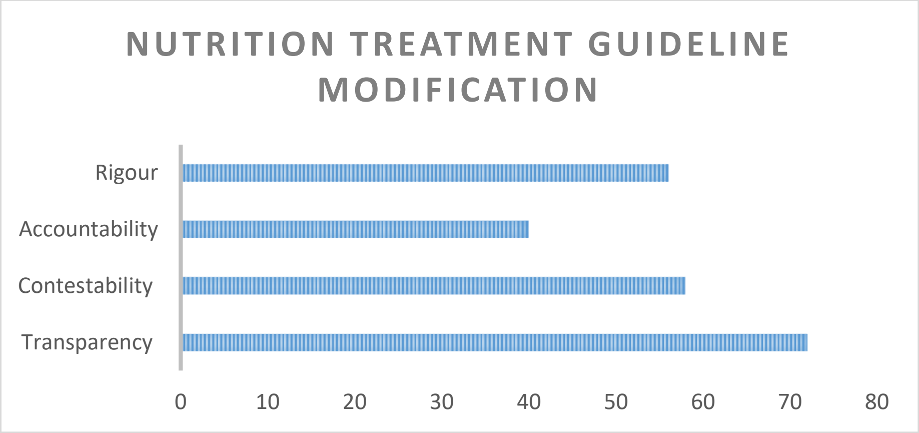

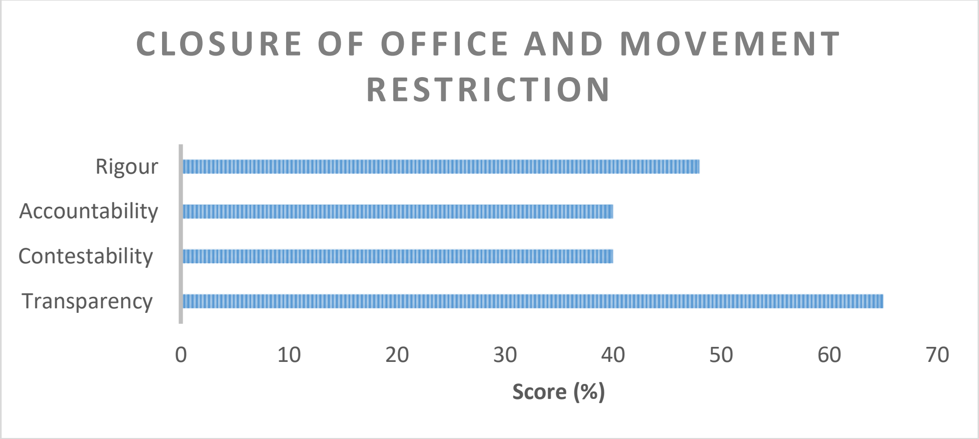

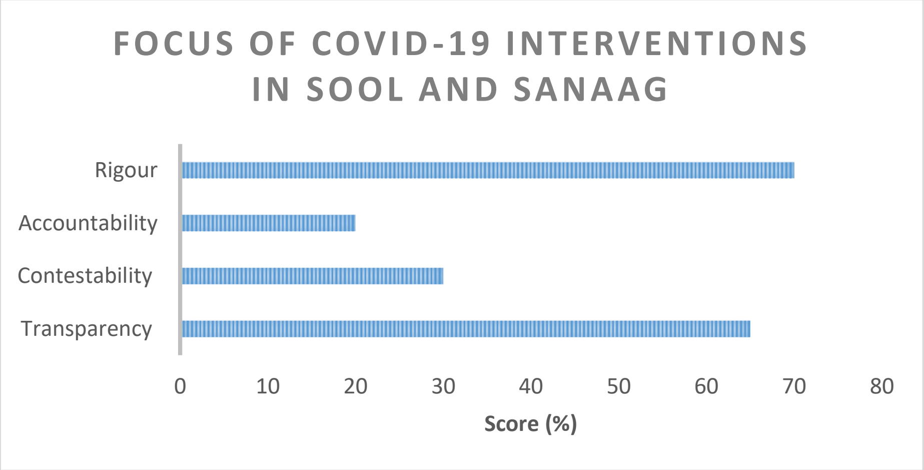

For peer review only - http://bmjopen.bmj.com/site/about/guidelines.xhtml

LONDON SCHOOL of HYGIENE &TROPICAL MEDICINE

Detailed Scores & Interpretation

1. Nutrition treatment guideline modification

Inclusivity 72%	Decision criteria 76%	Process 64%	Public rationale 76%

In terms of transparency, the decision to modify nutrition programme guidelines to be COVID-19 compatible was found to be fairly transparent. However, participants described a deficit in accountability as these modifications were reportedly made at the cluster level without the input of all partners. Additionally, participants reported that the affected communities were not consulted and could not feed into this decision-making process.

Devolution 44%	Revision 72%	Community engagement 40%	Explicit outcome 56%

Moreover, participants noted they did not have opportunity to contest these adaptations before they were adopted. They did however agree that there was a clear rationale to minimize transmission risk to the population. Nevertheless, participants did make clear the missed opportunity in engaging with the community to prepare them for these modifications.

Feasibility 55%	Strengthen Health system 60%	Evidence based 53%

Participants expressed concern that these adaptations would reduce patient follow-up, risk missing eligible program beneficiaries and could lower the effectiveness of the program.

2. Closure of Office and movement restriction

Inclusivity 60%	Decision criteria 76%	Process 64%	Public rationale 60%

The closure of CARE offices and restriction of staff movement was done early in the response. The decision was taken by the senior management and communicated via email to all staff. As a result, evaluation participants felt that this approach lessened the transparency as an opportunity was not provided to staff to feed into this decision. There was no clear documentation provided on the deliberations of this decision and the various options considered.

Participants did concede that it was possible that there were prioritization and decision-frameworks utilized but they were not disseminated to all staff.

Feasibility 60%	Strengthen Health system 40%	Evidence based 36%

Participants noted that in terms of supporting evidence, WHO and other international guidelines were possibly followed. No specific document was cited. However, the driving factor was to preserve business continuity and an abundance of caution as there were limited evacuation options for staff who felt ill.

Devolution 36%	Revision 44%	Community engagement 40%	Explicit outcome 60%

Participants mentioned that the decision to close offices was not devolved to sub-offices but was made at the country level. Additionally, there was limited community engagement with participants noting that as this could possibly affect program delivery, there should have been some level of community engagement.

For peer review only - http://bmjopen.bmj.com/site/about/guidelines.xhtml

LONDON SCHOOL of HYGIENE &TROPICAL MEDICINE

3. Focus of COVID-19 Interventions in Sool and Sanaag

Inclusivity 40%	Decision criteria 80%	Process 80%	Public rationale 60%

CARE has largely been conducting its humanitarian and development programming in rural areas prior to the pandemic. When the pandemic occurred, fundraising and response resources were focused to areas which CARE had a strong field presence. These were in Sool and Sanaag regions in largely rural areas. However, the majority of COVID-19 cases were in urban centres and in higher density areas of the country. Thus there was a mismatch between where resources were directed and where they were most required. Participants explained that as a result of this prioritization of existing programme areas, there was inadequate strategic focus and limited contestability in the decision.

Devolution 40%	Revision 20%	Community engagement 20%	Explicit outcome 60%

Participants reported limited community engagement. However, there was ample opportunities to revise this decision according to some participants. There was also sufficient devolution with the design of the COVID-19 intervention done by both senior management as well as some technical staff based in the sub-offices.

Feasibility 80%	Strengthen Health system 80%	Evidence based 60%

Participants did not feel that this decision strengthened the health system as much as it could have if resources were more precisely directed where needed. They did cite usage of early available WHO guidelines as having a role in how the decision was undertaken. Participants rated the feasibility criteria as high based on the consideration by decision-makers to implement the COVID-19 interventions in existing program areas.

Conclusion

Overall very few primary or secondary documents were specifically cited by participants as evidence for the decision-making criteria. Participants were aware to varying degrees that some documents such as minutes and guidelines did influence decisions but were largely unable to cite them for this evaluation. They instead relied primarily on their recollection of events and discussions with fellow participants to piece together the events that transpired.

For peer review only - http://bmjopen.bmj.com/site/about/guidelines.xhtml

LONDON SCHOOL of HYGIENE &TROPICAL MEDICINE

Recommendations

Decision-making

In order to improve the quality of decision-making in CARE's COVID-19 response and more broadly in its humanitarian responses, the following recommendations have been put forward:

1) Improve the inclusivity of key critical decision-making through expanding the membership of decision-makers to include junior staff and field staff.
2) Improve trust and buy-in of decisions through strengthening reporting of decision rationale and criteria.
3) Strengthen documentation of response activities to allow for more in-depth real-time or post response evaluation.
4) Consider more critically the relevance of scientific evidence and public health measures to the context rather than blanket adoption.
5) Allow for more contextually specific factors to shape decisions rather than blanket decisions

Evaluation Process

1) Allow for more time pre-evaluation in order for evaluation team to orient themselves with the methodology.
2) Translate evaluation tools and documentations into local language to allow for better understanding and involvement of evaluation participants
3) Allow for remote or asynchronous completion of evaluation tools by participants
4) Broaden the evaluation participants to include non-emergency staff and representatives of the community and local government
5) Consider practise scenario to allow for better uptake by evaluation team prior to commencing evaluation

For peer review only

For peer review only - http://bmjopen.bmj.com/site/about/guidelines.xhtml

LONDON SCHOOL of HYGIENE &TROPICAL MEDICINE

Annex

Evaluation Schedule

Step	Action	Responsible	Date
1	Recruitment of evaluation team and signing of consent forms	All participants	November 8th
2	First Part A- Draft of Context Analysis	CARE Evaluation Focal Person	November 11
3	Review of Part A- Context Analysis	All Participants	November 13th
4	Set up Meeting	LSHTM & CARE focal person	November 14th
6	Completion of Part B- Decision Selection	All participants	November 16
7	Decision Ranking Exercise	All Participants as group	November 16
8	Completion of Part C- Scoring of Decisions	All Participants separately	November 17
8	Completion of Part D- Overall Scoring	LSHTM	November 25th
9	Key Informant Interview	All Participants	November 18- 21st

For peer review only - http://bmjopen.bmj.com/site/about/guidelines.xhtml

LONDON SCHOOL of HYGIENE &TROPICAL MEDICINE

Score Criteria

Dimension	Criteria	Description
**Transparency**	Inclusivity	The extent to which the process was inclusive, reflected in heterogeneity in rank and roles amongst decision makers involved.
	Use of explicit decision-making criteria	The extent to which the goals and objectives of the decision were clearly pre-specified. The absence of post-decision rationalization.
	Following clear process or method	The extent to which a priority setting process was in place, reflected in demonstrated use of priority setting frameworks, decision trees or other mechanism.
	Use of mechanism to publicise rationale	The extent to which clear documentation on the decision exists as well as the method used to communicate decisions.
**Contestability**	Opportunity for revision	The extent to which there existed scope to revise and overturn a decision including the debating of alternatives and description of how consensus was reached.
	Was the decision devolved?	The degree to which participants in closest proximity to the epidemic (e.g. subnational level) or local technical experts participate in the decision, including consideration of rank.
**Accountability**	Engagement with affected communities	The degree to which affected communities were involved in the response decision-making including at a minimum whether they were informed of the response activities and what effect this notification had on the communities.
**Rigour**	Explicit outcome	The extent to which intended outcomes of the decision were clearly articulated, including through setting of targets.
	Feasible outcome	The extent to which feasibility was considered in decision-making including debating of alternatives.
	Strengthens healthcare system	The extent to which the decision was in-line with wider strategy including the strengthening of the health system
	Evidence based	The extent to which the decision was based on strong public health rationale and robust scientific information.

For peer review only

For peer review only - http://bmjopen.bmj.com/site/about/guidelines.xhtml

BMJ Open

Auditing the quality of epidemic decision-making in Somalia: a pilot evaluation

Journal:	*BMJ Open*
Manuscript ID	bmjopen-2022-065122.R1
Article Type:	Original research
Date Submitted by the Author:	28-Nov-2022
Complete List of Authors:	Warsame, Abdihamid; London School of Hygiene and Tropical Medicine Department of Infectious Disease Epidemiology,<br>Ore, Abdulkadir; Care Somalia<br>Azad, Abdullah; World Health Organization Somalia<br>Hassan, Farhan; World Health Organization Somalia<br>Blanchet, Karl; University of Geneva Faculty of Medicine, Geneva Centre of Humanitarian Studies<br>Palmer, Jennifer; London School of Hygiene and Tropical Medicine Department of Global Health and Development,<br>Checchi, Francesco; London School of Hygiene and Tropical Medicine Faculty of Epidemiology and Public Health
**Primary Subject Heading**:	Global health
Secondary Subject Heading:	Public health, Qualitative research, Infectious diseases
Keywords:	Health policy < HEALTH SERVICES ADMINISTRATION & MANAGEMENT, International health services < HEALTH SERVICES ADMINISTRATION & MANAGEMENT, Organisational development < HEALTH SERVICES ADMINISTRATION & MANAGEMENT, Quality in health care < HEALTH SERVICES ADMINISTRATION & MANAGEMENT, Protocols & guidelines < HEALTH SERVICES ADMINISTRATION & MANAGEMENT, INFECTIOUS DISEASES

SCHOLARONE™
Manuscripts

I, the Submitting Author has the right to grant and does grant on behalf of all authors of the Work (as defined in the below author licence), an exclusive licence and/or a non-exclusive licence for contributions from authors who are: i) UK Crown employees; ii) where BMJ has agreed a CC-BY licence shall apply, and/or iii) in accordance with the terms applicable for US Federal Government officers or employees acting as part of their official duties; on a worldwide, perpetual, irrevocable, royalty-free basis to BMJ Publishing Group Ltd ("BMJ") its licensees and where the relevant Journal is co-owned by BMJ to the co-owners of the Journal, to publish the Work in this journal and any other BMJ products and to exploit all rights, as set out in our licence.

The Submitting Author accepts and understands that any supply made under these terms is made by BMJ to the Submitting Author unless you are acting as an employee on behalf of your employer or a postgraduate student of an affiliated institution which is paying any applicable article publishing charge ("APC") for Open Access articles. Where the Submitting Author wishes to make the Work available on an Open Access basis (and intends to pay the relevant APC), the terms of reuse of such Open Access shall be governed by a Creative Commons licence – details of these licences and which Creative Commons licence will apply to this Work are set out in our licence referred to above.

Other than as permitted in any relevant BMJ Author's Self Archiving Policies, I confirm this Work has not been accepted for publication elsewhere, is not being considered for publication elsewhere and does not duplicate material already published. I confirm all authors consent to publication of this Work and authorise the granting of this licence.

For peer review only

Auditing the quality of epidemic decision-making in Somalia: a pilot evaluation

Abdihamid Warsame[1]

Abdikadir Ore[2]

Abdullah Azad[3]

Farhan Hassan[3]

Karl Blanchet[4]

Jennifer Palmer[5]

Francesco Checchi[1]

[1]Faculty of Epidemiology and Population Health, The London School of Hygiene & Tropical Medicine, London, United Kingdom.

[2]CARE International Somalia. Garowe, Somalia

[3] Health Emergencies Unit. World Health Organization Somalia. Mogadishu, Somalia

[4]Geneva Centre of Humanitarian Studies, Faculty of Medicine, University of Geneva, Geneva, Switzerland

[5]Faculty of Public Health and Policy, The London School of Hygiene & Tropical Medicine, London, United Kingdom.

Corresponding author:

Abdihamid Warsame

Faculty of Epidemiology and Population Health, The London School of Hygiene & Tropical Medicine, London, United Kingdom.

Email address: Abdihamid.warsame@lshtm.ac.uk

For peer review only

Abstract

Objective

To assess decision making quality through piloting an audit tool among decision makers responding to the COVID-19 epidemic in Somalia.

Design and Setting

We utilized a mixed methods programme evaluation design comprising quantitative and qualitative methods. Decision-makers in Somalia piloted the audit tool generating a scorecard for decision-making in epidemic response. They also participated in key informant interviews discussing their experience with the audit process and results.

Participants

A total of 18 decision-makers from two humanitarian agencies responding to COVID-19 in Somalia were recruited to pilot the audit tool.

Outcome measures and analysis

We used thematic analysis to assess the feasibility and perceived utility of the audit tool by intended users (decision-makers). We also calculated Fleiss' Kappa to assess interrater agreement in the audit scorecard.

Results

The audit highlighted areas of improvement in decision-making amongst both organisations including in the dimensions of accountability and transparency. Despite the audit occurring in a highly complex operating environment, decision-makers found the process to be feasible and of high utility. The flexibility of the audit approach allowed for organisations to adapt the audit to their needs. As a result, organisation reported a high level of acceptance of the findings

Conclusion

The pilot evaluation illustrates that it is possible to systematically assess decision-making quality in an ongoing epidemic within a humanitarian setting. Improving decision-making quality can contributes to better response outcomes including more efficient allocation of resources, greater coordination, and saved lives

Strengths and limitations of this study

- The criteria for decision-making quality are listed and defined
- This study utilizes mixed methods to both audit quality of decisions as well as shed light on the utility and feasibility of the tool
- This study is limited to auditing organisational decision-making and does not attempt to audit individual decision-makers
- Only decisions related to a single epidemic within one setting were audited
- The audit process was organisationally led in order to determine the feasibility of the audit.

Introduction

Evaluating decision-making has been recognized as essential to improving health outcomes in a number of contexts[1]. In humanitarian and crisis contexts good decisions at program, sector and response level are especially critical to saving lives and improving response[2]. Despite recent calls for concerted evaluation of decision-making in these settings[3,4], the literature is still

For peer review only

sparse. In crisis-affected settings experiencing epidemics, evaluations to date have primarily focused on establishing the extent to which epidemic response outcomes (for example reduced transmission, improved case management) have been attained. Less attention has been given to evaluate the processes underlying these outcomes (for example how response activities were decided and implemented) [5]. Process evaluations have largely been conducted in high-income countries or after high-profile epidemics to retrospectively determine which decisions led to response failures[6–8].

The need to improve decisions in epidemic settings is especially relevant considering the ongoing COVID-19 pandemic, in which decision-makers contend with a plethora of competing emergencies[9]. Decision support tools have been developed for a variety of settings and purposes but are particularly ubiquitous in corporate business management[10] and in the pharmaceutical and health technology sector[11] for example in the development and licensing of medicinal products, equipment and diagnostics. They are less frequent in humanitarian contexts where they focus primarily on supporting decision-makers in optimizing selected response interventions through for example specifying target populations or modalities of delivery[12,13]. To our knowledge there does not exist an evaluation tool that examines the quality of decision processes within epidemic settings. In a previous study in Somalia, we described factors relevant to COVID-19 decision-making processes; results suggested a need for such a tool.[9]

We thus developed a decision-making audit tool to support epidemic responders in assessing and improving the quality of organisational decision processes. Here, we report on a pilot application of this tool amongst two epidemic response organisations in Somalia, a country grappling with the ongoing COVID-19 pandemic and ongoing humanitarian crises [14].

Aim and Objectives

The overall aim was to evaluate the utility and feasibility of a decision support tool for epidemic responders in humanitarian settings.

The specific objectives were to: (i) document the implementation of the tool among select epidemic responders in Somalia; (ii) generate epidemic decision-making scores using the

For peer review only

decision support tool; and (iii) explore the feasibility and utility of the tool through key informant interviews of epidemic responders involved in the audit. We refer to objectives 1 and 2 as comprising the audit while objective 3 is referred to as the evaluation.

Methods

Study Design

This study used a mixed methods programme/response evaluation design. It comprised quantitative and qualitative data collection to both assess the decision-making in epidemic response as well as the feasibility and perceived utility of the tool by intended users (decision-makers). We then revised the tool based on this data. The final structure of the decision-making evaluation tool was determined from input collected during the pilot evaluation.

Patient or public involvement

Neither patients or the public were involved in the design of this study.

Description of the tool

We developed an evaluation tool, protocol and Standard Operating Procedure (SOP) (Supplementary Files1-4) founded on previous reviews [15] and extensive fieldwork in an epidemic setting[9]. This foundational work resulted in findings of very low coverage of epidemic evaluations globally, limited focus on decision processes, lack of standardized evaluation methods as well as an absence of a comprehensive evaluation framework suitable for epidemic response. As a result, we developed an Adaptive Epidemic Response (AER) evaluation framework[16] and drawing from an assessment of the COVID-19 response in Somalia[9], we derived a decision-making framework. The tools and SOPS are based on this decision-making framework. The tool is comprised of three sections and is summarized below (Figure 1).

For peer review only

The first section (Part A) of the tool is the context analysis. which requires information on the historical, geographic and health context in which the epidemic is occurring.

The second section (Part B) entails identification of critical decisions in the response to the specific epidemic. These critical decisions are identified and selected with reference to five Critical Decision Characteristics that differentiate critical decisions from minor or low impact decisions[2] (Table 1).

Table 1. Characteristics of 'critical' decisions

Characteristic	Definition
Consequential	Decision shapes the response to a significant degree
Not reversible	Decision is difficult to overturn or reverse, at least in the short term
Strategic	Decision involves substantial shift in terms of action taken, resources committed or precedent set
Uncertain	'Decision is made in context of substantial uncertainty with complex array of options
Reputationally risky	Decision entails a high level of organisational reputational risk

In the third section of the tool (Part C), users assess each of the selected decisions against the criteria for 'quality' decision-making. Although there is no agreed definition of a good quality decision[17], we have derived a number of defining criteria from previous published research on health prioritization[18], organizational decision-making[17,19,20] as well as decision-making in emergencies[21]. The 11 criteria are grouped into 4 dimensions: transparency, contestability, accountability, and rigour (Table 2).

Table 2. Criteria for assessing decision quality

Dimension	Criteria	Description
**Transparency**	Inclusivity	The extent to which the process was inclusive, reflected in heterogeneity in rank and roles amongst decision makers involved.
	Use of explicit decision-making criteria	The extent to which the goals and objectives of the decision were clearly pre-specified. The absence of post-decision rationalization.
	Following clear process or method	The extent to which a priority setting process was in place, reflected in demonstrated use of priority setting frameworks, decision trees or other mechanism.

For peer review only

	Use of mechanism to publicise rationale	The extent to which clear documentation on the decision exists as well as the method used to communicate decisions.
**Contestability**	Opportunity for revision	The extent to which there existed scope to revise and overturn a decision including the debating of alternatives and description of how consensus was reached.
	Was the decision devolved?	The degree to which participants in closest proximity to the epidemic (e.g. subnational level) or local technical experts participate in the decision, including consideration of rank.
**Accountability**	Engagement with affected communities	The degree to which affected communities were involved in the response decision-making including at a minimum whether they were informed of the response activities and what effect this notification had on the communities.
**Rigour**	Explicit outcome	The extent to which intended outcomes of the decision were clearly articulated, including through setting of targets.
	Feasible outcome	The extent to which feasibility was considered in decision-making including debating of alternatives.
	Strengthens healthcare system	The extent to which the decision was in-line with wider strategy including the strengthening of the health system
	Evidence based	The extent to which the decision was based on strong public health rationale and robust scientific information.

Users are required to rate on a Likert scale (a type of linear rating scale commonly used to measure respondents' opinions or attitudes), the strength of evidence supporting the fulfilment of each criterion. They are also expected to provide reference to documentary or observational evidence to support their rating. Users' individual scores are then aggregated, and a summary score is generated.

Data Collection

Audit

We invited three organisations actively engaged in the COVID-19 response in Somalia to pilot the audit tool. After separate presentations in which the protocol and study objectives were explained by the first author, we partnered with two organisations (WHO Somalia and CARE Somalia) in September 2021. Both organisations then nominated audit focal persons tasked with recruiting relevant colleagues (a 'decision-making committee'), gathering the necessary

For peer review only

documentation, and completing part A of the tool. Focal persons were instructed to recruit colleagues who had an active role in decision-making within the response as well as to compile key documents which informed or documented decisions. The location and modality of the audit (combination of face to face and remote sessions) and timeline were jointly determined by the audit focal persons and the first author. Study information and consent forms were shared, and written consent was obtained from all participants in the pilot.

The decision-making audit and feasibility evaluation took place in Garowe, Somalia from November 8-22nd 2021 with CARE Somalia and in Mogadishu from November 22- December 8 with WHO Somalia. The audit was led by each organisation's focal persons with the facilitation of the first author. Each audit was comprised of 3 group sessions with the decision-making committee interspersed with individual sessions (Figure 2). The first group session introduced the audit approach, tool, timeline and expected outputs. Participants were then asked to review Part A (context analysis) of the tool and incorporate any changes before the next group session. In the second group session, participants were introduced to Part B (selection of critical decisions) and were tasked with individually generating their perceived list of critical decisions. In the third group session, participants were asked to each present and advocate for their selection of critical decisions to the wider group. The group was then tasked with forming a consensus on at least 3 of the decisions to carry forward to the last stage. After consensus was reached, participants were asked to complete Part C of the tool for each decision in which they assessed the availability and strength of evidence supporting the fulfilment of various quality decision-making criteria. Audit focal persons and the LSHTM researcher then aggregated the scores and created a draft scorecard.

Evaluation

All audit participants were then invited to take part in a key informant interview to expound on their selected decisions and explore their views on the evaluation process and tools. After the key informant interviews, the draft scorecard was shared with all participants to provide individual

For peer review only

written feedback on the results. The final scorecard was then developed by the audit focal persons and first author and shared with the senior management of the respective organisation. Finally, key informant interviews were conducted by the first author with senior managers who were not involved in the other aspects of the audit and evaluation to solicit their views on the results and their understanding of the process. The tool and related documents were then updated based on pilot findings.

A total of 18 key informant semi-structured interviews were conducted in English or Somali by the first author either face-to-face or via Zoom (Table 3). The first author is a fluent Somali speaker who has substantial experience in health evaluations and epidemic response in the Somali context. The topic guide covered participants' views on the feasibility of the audit process including time and human resources required, the utility of the audit tool in understanding and improving decision making as well as recommendations for future iterations.

Table 3 Data collection by method and type of participant or setting

Primary Data collection	Type of Participants	Number of Participants
**Key informant interviews**	WHO audit team	7
	WHO Staff- Other	2
	WHO Senior management	2
	CARE Somalia audit team	5
	CARE Somalia senior management	2
**Group Sessions**	WHO audit Team	7
	CARE audit Team	6

Interviews were recorded for transcription and analysis purposes. Each interview took approximately 30-45 minutes and participants were given the option of complete or partial anonymity in which they considered whether they would like their name or only their role to be published. A total of 13 participants took part in the group sessions and notes were taken by the first author. All participants in the CARE audit were national staff members while the majority of WHO participants were international (85%).

For peer review only

Data Analysis

Generation of audit tool scoring

The scorecard for the audit was produced by calculating for each of the 11 criteria assessed, the average of the individual participants scores. Average scores were also presented according to the four dimensions listed in table 2.

Evaluation of the audit implementation

In order to evaluate the implementation of the audit tool as it relates to the feasibility and utility, interview transcripts and group session notes were analysed using a thematic approach[22] using Nvivo software. Emerging themes were grouped into two categories: those relating to identified critical COVID-19 decisions and those relating to the implementation of the audit. Lastly the validity of the tool was evaluated. The interrater agreement of each organisation's scoring was calculated using Fleiss' Kappa.

In the following results section, we focus on the implementation of the audit while details of specific decisions can be found within the decision-making scorecards in the supplemental material (Supplementary File 5, Supplementary File 6).

Results

Implementation of the decision-making audit

Participants in both organisations opted for a mixture of face-to-face and remote (video conference) data collection citing the busy schedule and geographic dispersion of the participants. Although preparatory material including documentation was shared prior to the arrival of the LSHTM focal person, some participants were not immediately aware of the purpose of the audit. The delayed setup of shared folders containing key audit documentation proved difficult for participants' engagement with the audit and subsequent evaluation. Furthermore, the period in which this audit took place coincided with multiple projects including end-of-year evaluations, annual planning and multiple pre-scheduled activities, hindering the timely recruitment of participants. Many participants were not able to be physically present in the

For peer review only

location of the audit due to competing engagements but were nevertheless present through remote means.

Decision Selection

CARE participants had difficulty identifying critical decisions with individual participants submitting on average only 1 critical decision for consideration. However, consensus was reached fairly quickly once the proposed decisions were deliberated by the wider group. In contrast, WHO audit participants put forward 15 decisions for consideration. Consensus took longer to achieve as participants vigorously advocated for their proposed individual decisions. Eventually the group reached consensus on 4 decisions to proceed to the next stage (Table 4). In both groups, moderate reference was made to the Critical Decision Characteristics (Table 1) with participants arguing for decisions in their particular area of work. Participants in WHO were particularly inclined to view decisions in terms of their alignment with the Incident Management Support Team (IMST) Pillars[23]. Additionally, there was an effort by some participants to reach consensus by collapsing together multiple decisions into a single decision in order to capture all opinions. In both WHO and CARE evaluations, participants focused on positive decisions (i.e. a decision to take an action) and may have overlooked negative decisions (i.e. decisions not to take action).

Table 4. List of selected critical decisions by organisation

CARE Somalia	WHO Somalia
Closure of offices and restriction of staff movement	Scale up of case management through improving access to therapeutic oxygen
Modification of Nutrition programme guidelines to be COVID-19 sensitive including changing patient assessment, facility management and outreach procedures	Establishment of 3 key PCR labs in Mogadishu, Garowe and Hargeisa to strengthen diagnostic and surveillance capacity
Scale up of COVID-19 response activities such as community outreach, contact tracing, provisioning of IPC supplies in Sool and Sanaag Regions	Implementation of the Incident Management Support Team to coordinate COVID-19 response
	Establishment of rapid response teams to scale up surveillance capacity within high priority districts

For peer review only

Decision Scoring

Participants in the CARE audit on average scored their decision-making quality lower than those of WHO. Both organisations scored lowest in accountability to target populations. The scoring of WHO participants on the decision to focus on case management through oxygen scale up demonstrates the low scoring of accountability relative to the other dimensions of quality decision-making (Figure 3). Full details can be found in the supplementary materials (Supplementary File 5, Supplementary File 6).

Participants in CARE scored their decisions lower in contestability compared to WHO. Both organisations rated themselves highly in transparency, particularly in the inclusivity criteria. However, the key stakeholder mentioned under these criteria differed, with CARE highlighting the need to improve inclusive decision-making within the organisation (national staff vs country office senior management) while WHO referred to inclusivity with government authorities. Lastly, CARE participants rated their decision-making as less rigorous than WHO's, citing their unfamiliarity with the evidence underlying the decisions. By contrast, WHO participants cited use of the latest scientific findings and alignment with health systems strengthening frameworks.

Interrater agreement

There was very low interrater agreement amongst participants in both WHO (Kappa = -0.000948, P= 0.977) and CARE (Kappa = 0.0684, P= 0.172) audit groups with a slightly higher interrater agreement amongst CARE participants, indicating high heterogeneity of scores.

Perceptions of the audit tool

Utility

Participants described clearly seeing the purpose and value of the audit. For example, one participant from WHO said, "*We learned that decisions are not just simply taken as decisions, but you have to have a clear decision-making process, a methodology, or criteria, or something, but not necessarily that we just come and say, "Oh, let's do this and that"*. Participants articulated the ongoing need for such an exercise, arguing: "*If there's no understanding of how a decision*

For peer review only

happened, there's no way to improve on it" (WHO Participant 5). Critically, they understood that this exercise was meant as an internal audit: *"It was really for us to be able to critically evaluate ourselves as an organisation" (CARE Participant 3).* Some participants also saw the potential utility in a wider range of contexts, beyond epidemics: "*I think [the audit tool] can apply to any decision in humanitarian emergency response" (WHO Participant 2).*

Participants mentioned learning new things about the decision process including recognizing neglected characteristics of good decision-making:

> *"When I was trying to fill in some of the scores, I realized that these are very important [aspects] that were neglected before. For example, devolving certain decisions, it is very important. Not just like you sit up at national level, you decide for anyone and you decide for people at all levels, I think if we devolve certain functions it is good". WHO Participant 1*

They also were able to identify areas for improvement. "*I think for accountability most of the evidence was very minimal. It just got me thinking of ways in which we can involve the communities in decision-making." (WHO Participant 4).*

Participants also highlighted that the audit was useful in allowing them to be more reflective. *"It helped me look at things objectively. If I was not part of the Incident Management Support Team (IMST) would I have advocated for this decision or not?" (WHO Participant 4).* They highlighted the relevance of the decision-making critical characteristics as useful in their work and recognised these as reflective of best-practice: *"I think the tool helped a lot to give me a reflection as to what should be done. I think I learned a lot going through the questions." (WHO Participant 1).*

Participants mentioned the gradual understanding they accrued throughout the audit process as strengthening their engagement with the audit. *"I think some of it was clear, but some of it was also not very clear. I think I understood the methodology with time, of course, when you explained and when we were having the group discussions. That is why in the last forms, I revised and added a few components and sent back to you. I think as usual when people read*

For peer review only

something, it might not be clear in the first place, but when you explain it, it becomes clear". (WHO Participant 4).

Participants also noted the benefit of the group format in deriving new insights and creating a useful forum for reflection to review decisions that otherwise didn't exist in their typical organisational practices:

> *"We all had our own ideas, but when we met as a group, there were a few other things that came out that probably I didn't think about as an individual, which were very valid". WHO Participant 4*

> *"I also liked that we were able to meet as a group and look at these things because to be very honest, I don't feel like we have that as a group, as an IMST team to review the decisions that we made or look at what we could have done differently, or whether we felt this decision was important or not. This was a very unique exercise and I'm glad that we got an opportunity to do this." WHO Participant 7*

Participants expressed their commitment to use the lessons learned in future decision making:

> *"I will inform my colleagues, 'Please, let us have certain criteria to use, let us make our methodology clear, and let us document everything,' because at some point down the road, we should know our decision-making process. This has helped a lot and will change a lot in the future." WHO Participant 5*

However, participants mentioned that there was a certain element of subjectivity which was difficult to overcome:

> *"It might be very difficult to understand exactly the reality on the ground, because it depends on the person. Not only the person but also, the involvement of the person in the response activity, and also the understanding of the context. If you are someone who is quite new to Somalia, and maybe who doesn't have detailed involvement, you might give a different ranking than a person who's spent more time in Somalia and directly engaged in response activity". WHO Participant 2*

Feasibility

In terms of the audits' feasibility, participants expressed that they were easily able to comprehend the process. *"[The tool] was very understandable and also, it was very user friendly." (CARE Participant 1)*. However, a few participants noted some areas for improvement: *"I'm looking at this tool being used for an outbreak. If that is to happen, as I said, we need to*

For peer review only

really condense some aspects, and lump some of these criteria together to cut on time." (CARE Participant 3).

In terms of time, participants expressed that the duration of the audit was acceptable but that the time of year chosen for the implementation could have been improved. *"In terms of length of the time, this is fine. This amount of time we need to give. The issue is the timing of the programming, because in some of the months, we are very busy, like last quarter of the year we are very busy." (CARE Participant 5).*

As a result, some participants expressed their confidence that they could implement this audit independently at a more opportune time. However, others expressed their preference for this work to be led by an independent evaluator.

> *As the independent outsider, everybody can open up to you, and this is the best advantage I can see. I also saw something similar in my previous organization, that again, it is the power dynamic or the personality thing that not everybody speaks openly, or the culture doesn't allow it but when somebody from outside comes, everybody opens up. At the end, you come up with some really good findings and recommendations" WHO Participant 1*

In their engagement with the audit tool, participants faced a challenge in coming to a consensus on the limited number of decisions to score. *"Considering the response that we are doing in the country, it might be a bit challenging to come up with one or two out of so many critical decisions." (WHO Participant 2).*

Additionally, there were challenges in accessing documentation relevant to the audit. "*Maybe the big challenge is getting the evidence, documentation, preparing the documentation, extracting the email, finding the document, it might be time-consuming." WHO Participant 3*. Where documents did exist, some participants stated that not all staff may have access. Lastly, some participants highlighted language or conceptual understanding barriers as challenges in the audit. *"At some point, I felt like we were not looking at the decisions, but we were looking at pillars." (WHO Participant 4).*

For peer review only

Selection of decision-making committee

Participants noted that the composition of the decision-making committee had the potential to affect the audit outcome because of a lack of detailed knowledge on how decisions were made. "*If somebody is not involved in a certain pillar, they would struggle to look for evidence or they might not be able to direct somebody to a particular document." (WHO Participant 2)*

They mentioned that all who were part of the audit had some role to play in COVID-19 decision making but conversely not all who had decision-making roles participated in the audit.

Some participants highlighted the potential of overlooking critical decisions when the primary responsible staff member was not present in the audit.

> *"Later I thought about COVID-19 vaccine, and I wondered if we could have included the focal person and whether they would have felt that the introduction of the COVID-19 vaccine into the country was a critical decision. I thought about it, and I thought maybe nobody mentioned it, or nobody talked much about it during the discussion because the focal point was not there. WHO Participant 4*

Participant Recommendations

Participants made several recommendations to improve the utility and feasibility of the audit for future iterations. These included broadening the scope of participants by more clearly outlining who should participate.

Participants also suggested incorporating the emergency response cycle into the evaluation tool. *"There are emergency response cycle phases such as preparedness, implementation, monitoring, and evaluation […] There are critical decisions at each phase which need to be evaluated." (WHO Participant 2)*

They also made several suggestions to improve the implementation including ensuring more face-to-face rather than virtual interactions amongst participants and undertaking a joint review of documentation to improve participant familiarity with the available documents.

Views of senior management

Senior management largely agreed with the findings of their respective scorecards. They did, however, provide further detail and rationale for some aspects of the selected decisions. For

For peer review only

401 example, senior managers in both organisations cited mitigating factors in explaining lower-than-expected scoring in some dimensions. Referring to closure of offices and restriction of staff movements, one CARE senior manager explained:

"When the first case of COVID-19 was reported in Somalia in March, I think it was around 16, 17. There was a bit of panic in that it was not business as usual. When it comes to between life and death, certain positions have to be taken. As a result of that, yes, the senior management team made a decision to ensure that because we are accountable to everyone and every staff, and every staff exposed to any risk as a result of negligence of the organization, then the organization takes responsibility." CARE Senior Manager 1

A WHO senior manager explained the circumstances behind a decision to increase testing capacity:

"Obviously there's scope for improvement, but sometimes when we are in a pressured situations and we see that the PCR based labs are overwhelmed and at the peripheral level there is no access or testing facilities are not there, and the GeneXpert machines are also not functioning, then sometimes we make decisions and implement swiftly, and then [later] we provide other evidence and information of the value and effectiveness of those strategies". WHO Senior Manager 1

They did however agree on gaps highlighted through the scorecard stating: "*I would say that [decision-making] can become more inclusive. I think that we are just talking internally amongst ourselves and we are not engaging sufficiently with our government counterparts. I think that that's a fundamental mistake that we are doing, and we continuously do so, because of maybe convenience, because of maybe comfort.*" (WHO Senior Manager 2).

Discussion

This pilot evaluation of a tool to audit decisions taken during epidemic response demonstrates that the tool can be successfully deployed even in the midst of an ongoing response and even in circumstances where responders are dealing with unusually high workload. The audit also elicited very positive user feedback with participants expressing a willingness to implement it in future epidemic responses. Importantly, participants viewed the audit as reflecting good decision-making practice indicating high levels of acceptability.

For peer review only

As populations affected by epidemics continue to grow[24], there is an urgent need to improve epidemic response particularly through improved decision-making. Much of the focus has been on improving decision outcomes and less attention has been paid to decision-making processes[25]. However, strengthening decision processes can improve decision-making quality[26] and, accordingly, outcomes[27] including more efficient allocation of resources, improved accountability and greater coordination.

The literature on decision-making in emergencies has been underpinned by an analytically oriented[28] conceptualization of decision making in which the 'correct' decision is sought[29,30]. This is in contrast to the process conceptualization in which the decision is pursued correctly. Towards this end, decision support approaches have been developed to optimize the decision-making process to achieve efficient[2] and timely decisions[31] but have largely not considered the aspect of quality. The evaluation of this tool within the COVID-19 response in Somalia contributes towards filling this gap.

The audit sheds light on shortcomings in the quality of decision processes within piloting organisations and provided participants opportunity for reflection and key areas for improvement. The tool was found to be highly adaptable as it allowed organizations to evaluate decisions that were considered significant by their staff. Nevertheless, while the audit instructed organisation to select participants that were largely reflective of staff making or implementing response decisions, some participants expressed reservations about the final composition of the evaluation panel. The scoring of some decision dimensions was however largely reflective of the panel's experiences and characteristics. For example, amongst CARE participants, the lower scoring on the contestability of some decisions might be due to their roles as national staff who may not have had sufficient opportunity to contest decisions. The rollout of the tool allowed for gradual understanding to develop and for participants to question their basic assumptions. For example, participants were able to acknowledge elements that were absent from their decision-making process. They were also surprised by the paucity of documentary evidence within their organisations and were able to contrast this with their initial self-assessed high scoring. The lower-than-expected agreement amongst raters could possibly due to lack of consensus on what

For peer review only

various levels of evidence represented. While limited documentation does not necessarily equate with poor decision processes, it does make subsequent evaluations more challenging.

Furthermore, the audit tool was found to be highly flexible as users highlighted its potential utility in assessing the quality of decision-making in broader humanitarian response by generating a quantitative measure of decision quality that can allow for tracking over time. In addition to the retrospective assessment of decisions, the tool can also be used in real time to improve decision-processes.

Limitations

Although this pilot was undertaken within two organisations, it assessed decision-making related to a single epidemic within one country. While this pilot study was led by the first author who was external to both organisations, future iterations led entirely by internal staff members may be more vulnerable to censorship if results reveal low decision quality. However this is a challenge that is present in global health arena in general[32] and a number of resources have been published to strengthen independence of evaluations[33–35]. Additionally, much of the scoring relied on the subjective assessment of the individual rater and may thus have been reflected in the lower-than-expected interrater correlation. Furthermore, this pilot focused on face validity (the extent to which a tool appears to measure a concept) rather than on content validity which requires further exploration and methods. Additionally, we did not use quantitative methods such as factor analysis to reduce the number of items within the critical decision criteria as there were too few items. Instead, we relied exclusively on direct feedback from the participant interviews. Definitions for the decision- making criteria were not exhaustive and could have been further expanded. For example with regards to community engagement criteria we provided a minimum definition rather than broader definition[36]. Finally, the methods described in this study assessed only organisational decision-making rather than individual and as such cannot be used to assess the decision-making of individual epidemic responders.

Recommendations

The audit can be further piloted in a wider range of crisis settings and amongst different response actors to ascertain its feasibility and utility in diverse settings. Additionally, the audit should be

For peer review only

conducted internally by response actors in order to compare how the audit is implemented when it is entirely independent of an external facilitator. Furthermore, the audit should be conducted periodically in order to determine whether there has been a quantitative change in the decision quality scoring. Lastly, the audit tool could also be integrated within the WHO recommended intra-action reviews[37] as well as after action reviews[38] for health emergencies.

Conclusion

Strengthening decision-making processes is key to realizing the objectives of epidemic response. This pilot evaluation contributes towards this goal by the testing what, to our knowledge, may be the first tool designed specifically to assess quality of decision-making processes in epidemic response. The tool has proven feasible and acceptable in assessing decision-making quality in an ongoing response and has potential applicability in assessing decision-making in broader humanitarian response.

For peer review only

Declarations

Contributorship statement

This study was designed by AW with input from FC. The data collection was undertaken by AW, AO, FH and AA. The analysis was undertaken by AW with input from KB, FC, and JP. The first draft was written by AW. All authors have contributed significantly to the final draft.

Competing interests

We declare no competing interests.

Funding

This work was supported by UK Research and Innovation as part of the Global Challenges Research Fund, grant number ES/P010873/

Data sharing statement

All data relevant to the study are included in the article or uploaded as supplementary information

Ethics approval and consent to participate

Ethical approval for this study was obtained from the National Institute of Health Research of the Federal Republic of Somalia (Ref: NIHS0102208) and the ethics review committee of the London School of Hygiene & Tropical Medicine (Ref: 26369). Informed written consent was taken from all key informants.

Acknowledgements

We would like to acknowledge Md Mamunur Malik WHO country representative and Iman Abdullahi CARE country representative for agreeing to pilot this tool in their respective organisations. We would like to thank participants for sharing their insights.

Reference

1 Glarum J, Adrianopoli C. *Decision Making in emergency management*. 1st ed. Oxford: : Elsevier 2020.

2 Cosgrave J. Decision making in emergencies. *Disaster Prev Manag An Int J* 1996;**5**:28–35. doi:10.1108/09653569610127424

3 Campbell L, Knox P, Alnappaper C. *Making operational decisions in humanitarian response: A literature review*. 2018. https://www.alnap.org/our-topics/leadership. (accessed 3 Jun 2020).

4 Knox Clarke P, Campbell L. Decision-making at the sharp end: a survey of literature related to decision-making in humanitarian contexts. *J Int Humanit Action* 2020;**5**:1–14. doi:10.1186/s41018-020-00068-2

5 Warsame A, Murray J, Gimma A, *et al.* The practice of evaluating epidemic response in humanitarian and low-income settings: a systematic review. *BMC Med* 2020;**18**. doi:10.1186/s12916-020-01767-8

6 Horton R. Offline: The mistakes we made over Ebola. Lancet. 2019;**394**:1494. doi:10.1016/S0140-6736(19)32634-0

7 S. M, D. S, M.A. P, *et al.* Will Ebola change the game? Ten essential reforms before the next

pandemic. the report of the Harvard-LSHTM Independent Panel on the Global Response to Ebola. *Lancet* 2015;**386**:2204–21. doi:http://dx.doi.org/10.1016/S0140-6736%2815%2900946-0

8 García-Basteiro A, Alvarez-Dardet C, Arenas A, *et al.* The need for an independent evaluation of the COVID-19 response in Spain. Lancet. 2020;**396**:529–30. doi:10.1016/S0140-6736(20)31713-X

9 Warsame A, Fuje M, Checchi F, *et al.* Evaluating COVID-19 decision-making in a humanitarian setting: the case study of Somalia. *Plos Glob Public Heal* 2022.

10 Stenfors S, Tanner L, Syrjänen M, *et al.* Executive views concerning decision support tools. *Eur J Oper Res* 2007;**181**:929–38. doi:10.1016/J.EJOR.2006.06.024

11 Bujar M, McAuslane N, Walker SR, *et al.* Evaluating quality of decision-making processes in medicines' development, regulatory review, and health technology assessment: A systematic review of the literature. *Front Pharmacol* 2017;**8**:189. doi:10.3389/FPHAR.2017.00189/BIBTEX

12 Immunization, Vaccines and Biologicals Vaccination in acute humanitarian emergencies: a framework for decision making. Published Online First: 2013.http://www.who.int/immunization/sage/sage_wg_hum_emergencies_jun11/en/ (accessed 13 Feb 2022).

13 Vargas Florez J, Lauras M, Okongwu U, *et al.* A decision support system for robust humanitarian facility location. doi:10.1016/j.engappai.2015.06.020ï

14 UNOCHA. Humanitarian Response Plan Somalia Humanitarian Programme Cycle 2021 Issued February 2021. 2021. www.unocha.org/somalia (accessed 22 Apr 2021).

15 Warsame A, Blanchet K, Checchi F. Towards systematic evaluation of epidemic responses during humanitarian crises: A scoping review of existing public health evaluation frameworks. *BMJ Glob Heal* 2020;**5**. doi:10.1136/bmjgh-2019-002109

16 Warsame A, Blanchet K, Checchi F. Towards systematic evaluation of epidemic responses during humanitarian crises: A scoping review of existing public health evaluation frameworks. *BMJ Glob Heal* 2020;**5**:e002109. doi:10.1136/bmjgh-2019-002109

17 Wood NL, Highhouse S, Brooks M, *et al.* INDIVIDUAL DIFFERENCES IN DECISION-MAKING STYLES AS PREDICTORS OF GOOD DECISION MAKING. 2012.

18 Kapiriri L, LaRose LB. Priority setting for disease outbreaks in Uganda: A case study evaluating the process. *Glob Public Health* 2019;**14**:241–53. doi:10.1080/17441692.2018.1498532

19 Peniwati K. Criteria for evaluating group decision-making methods. *Math Comput Model* 2007;**46**:935–47. doi:10.1016/J.MCM.2007.03.005

20 Bang D, Frith CD. Making better decisions in groups. R. Soc. Open Sci. 2017;**4**. doi:10.1098/rsos.170193

21 Knox Clarke P. How can we improve decision-making in humanitarian coordination? 2016. www.alnap.org/coord-meeting. (accessed 27 Aug 2020).

22 Bryman A. *Social Research Methods*. Oxford: 2012.

23 World Health Organization (WHO). Emergency Response Framework Second Edition. Taiwan Rev. 2017;**69**:76.

For peer review only

24 OCHA. Global Humanitarian Overview 2021 [EN/AR/FR/ES] - World | ReliefWeb. https://reliefweb.int/report/world/global-humanitarian-overview-2021-enarfres (accessed 18 Mar 2021).

25 Warsame Id A, Fuje Id M, Id FC, *et al.* Evaluating COVID-19 decision-making in a humanitarian setting: The case study of Somalia. *PLOS Glob Public Heal* 2022;**2**:e0000192. doi:10.1371/JOURNAL.PGPH.0000192

26 George B, Desmidt S. Strategic-Decision Quality in Public Organizations: An Information Processing Perspective: *https://doi.org/101177/0095399716647153* 2016;**50**:131–56. doi:10.1177/0095399716647153

27 Schippers MC, Rus DC. Optimizing Decision-Making Processes in Times of COVID-19: Using Reflexivity to Counteract Information-Processing Failures. *Front Psychol* 2021;**12**:2261. doi:10.3389/FPSYG.2021.650525/BIBTEX

28 Sanderson C. Analytical models for decision-making. *Open Univ Press* 2006.

29 Berger L, Berger N, Bosetti V, *et al.* Rational policymaking during a pandemic. *Proc Natl Acad Sci U S A* 2021;**118**. doi:10.1073/PNAS.2012704118/-/DCSUPPLEMENTAL

30 Morgan O. How decision makers can use quantitative approaches to guide outbreak responses. Philos. Trans. R. Soc. B Biol. Sci. 2019;**374**. doi:10.1098/rstb.2018.0365

31 Gouglas D, Marsh K. Prioritizing investments in new vaccines against epidemic infectious diseases: A multi-criteria decision analysis. *J Multi-Criteria Decis Anal* 2019;**26**:153–63. doi:10.1002/MCDA.1683

32 Storeng KT, Abimbola S, Balabanova D, *et al.* Action to protect the independence and integrity of global health research. *BMJ Glob Heal* 2019;**4**:e001746. doi:10.1136/BMJGH-2019-001746

33 Sager F, Plegger L. Evaluation and Independence Existing evaluation policies and new approaches. 2016.

34 OECD. PRINCIPLES FOR EVALUATION OF DEVELOPMENT ASSISTANCE. Paris: 1991.

35 Evaluation W office for. Decentralized evaluation for evidence-based decision making WFP Office of Evaluation Decentralized Evaluation Quality Assurance System (DEQAS) Technical Note Independence and Impartiality.

36 Palmer J. How is community engagement normally done? | COVID-19 Hygiene Hub Resources. 2022.https://resources.hygienehub.info/en/articles/4028678-how-is-community-engagement-normally-done (accessed 14 May 2022).

37 Mayigane LN, de Vázquez CC, Vente C, *et al.* The necessity for intra-action reviews during the COVID-19 pandemic. *Lancet Glob Heal* 2020;**8**:e1451. doi:10.1016/S2214-109X(20)30414-9

38 WHO. The global practice of after action review. Geneva: 2019. https://www.who.int/publications/i/item/WHO-WHE-CPI-2019.9 (accessed 16 Feb 2022).

Figure Legend

Figure 1 Structure of the audit tool

Figure 2 Data collection timeline of evaluation

For peer review only

Figure 3 Extract of decision scorecards where accountability was rated low

For peer review only

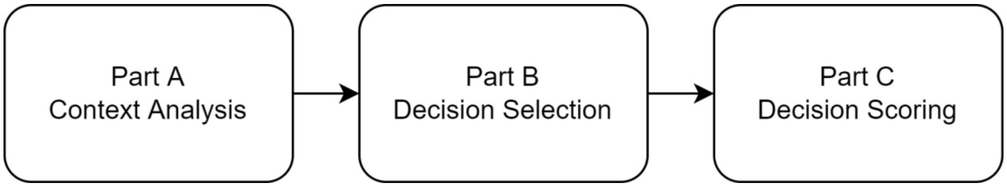

Figure 1 Structure of the audit tool

636x114mm (72 x 72 DPI)

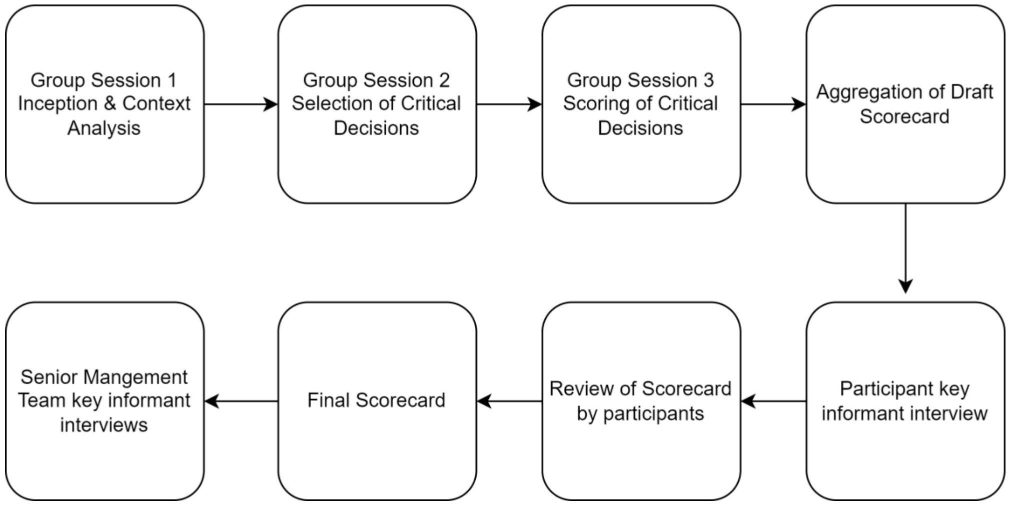

Figure 2 Data collection timeline of evaluation

855x424mm (72 x 72 DPI)

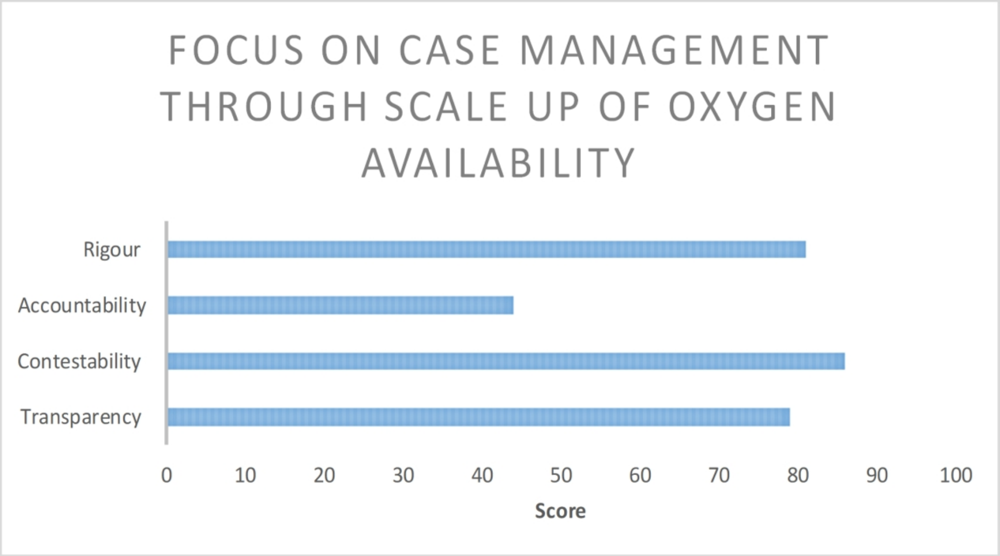

Figure 3 Extract of decision scorecards where accountability was rated low

871x484mm (59 x 59 DPI)

Evaluating the quality of epidemic decision-making in epidemics & humanitarian crises

Standard Operating Procedures (SOP)

For peer review only

Version 1.0
June 30th, 2021

Abdihamid Warsame
Infectious Disease Epidemiology
London School of Hygiene & Tropical Medicine
Abdihamid.warsame@lshtm.ac.uk

Contents

Evaluating epidemic decision-making in humanitarian crises ... 1
Standard Operating Procedures (SOP) ... 1
Abbreviations ... 4
Introduction ... 5
Rationale ... 6
Definitions ... 6
Target audience ... 6
Evaluation team composition ... 6
Timeline ... 7
Section 1- Context analysis ... 8
1.1 Purpose ... 8
1.2 Roles and responsibilities ... 8
1.3 Procedures ... 8
1.4 Outputs ... 8
1.5 Associated Documents ... 8
Section 2- Decision Selection ... 8
2.1 Purpose ... 9
2.2 Roles and Responsibilities ... 9
2.3 Procedures ... 9
2.4 Outputs ... 9
2.5 Associated Documents ... 9
Section 3- Decision Scoring ... 10
3.1 Purpose ... 10
3.2 Roles and responsibilities ... 10
3.3 Procedures ... 10
3.4 Outputs ... 10
3.5 Associated Documents ... 10
Section 4- Dissemination/ Review ... 11
4.1 Purpose ... 11
4.2 Roles and responsibilities ... 11
4.3 Procedures ... 11
4.4 Outputs ... 11
4.5 Associated Documents ... 11
Reference ... 12

For peer review only - http://bmjopen.bmj.com/site/about/guidelines.xhtml

Annex 13
Document Checklist 13
Decision-making evaluation tool .... **Error! Bookmark not defined.**
Background .... **Error! Bookmark not defined.**
Part A- Context analysis .... **Error! Bookmark not defined.**
Part B- Selection of critical decision points .... **Error! Bookmark not defined.**
Key informant interview guide .... **Error! Bookmark not defined.**
Flow chart .... **Error! Bookmark not defined.**

For peer review only

For peer review only - http://bmjopen.bmj.com/site/about/guidelines.xhtml

Abbreviations

CO- Country Office

COVID-19- Coronavirus disease 2019

RO- Regional Office

HQ- headquarters

Sitrep- Situation report

PHSA- Public Health Situation Analysis

RRA- Rapid risk assessment

SMT- Senior Management Team

KII- Key Informant Interview

FGD- Focus Group Discussion

For peer review only

For peer review only - http://bmjopen.bmj.com/site/about/guidelines.xhtml

Introduction

While epidemics of infectious disease continue to pose a considerable threat to populations in low-income and humanitarian settings, the evaluation of these epidemic responses are limited and heterogeneous [1]. Populations in need of humanitarian assistance or living within low income settings continue to grow[2] and the ongoing COVID-19 pandemic has further exacerbated the impact of these crises[3]. Decision-making in the context of an ongoing epidemic is particularly challenging[13,14]. Previous research has identified a limited number of decision-making factors contributing to poor response [15–17]. However, these findings need further study in order to confirm their relevance to a wider array of contexts experiencing ongoing epidemics

Such settings not only must deal with the epidemic directly and its indirect impacts but must also contend with a host of urgent competing emergencies. As such decision-makers are under heavier strain than those in better resourced and stable environments and require adequate support. COVID-19 is unlikely to be the last epidemic faced by decision-makers in such settings and therefore evaluations such as this are especially critical to strengthening future preparedness and improving response.

In decision-making research, there are two common approaches: process and evaluation. Process oriented approach describes how decisions are actually made within a specific context whereas analytical oriented is more prescriptive and frames decision-making as a problem-solving approach intended to lead to the best outcome. In short the former looks at whether a decision is made correctly while the latter considers whether the correct decision is made. This evaluation is predicated on the former approach.

For peer review only - http://bmjopen.bmj.com/site/about/guidelines.xhtml

Rationale

- This SOP is intended to evaluate the process of epidemic decision-making rather than the impact of any specific decision. Improved decision-making practises can improve outcomes, timeliness and accountability in epidemic response in humanitarian settings.

- In order to contribute to better humanitarian performance, the UKRI awarded a consortium led by the London School of Hygiene & Tropical Medicine to undertake research and produce evidence to support humanitarian responders. This SOP includes stepwise actions required to be undertaken by evaluators as well as the necessary timeframes, roles and responsibilities and resources required.

Definitions

Decision making: choosing between number of options/course of action or a process of problem solving in a specific context (Campbell 2018)

Evaluation- assessment, as systematic and impartial as possible of an activity, project, programme, strategy, policy, theme, sector, operational area or institutional performance.

Target audience

- This standard operating procedure is intended for the evaluation lead within the humanitarian organization. However, it can also be utilized by third party monitors as part of an overarching review of a humanitarian organizations epidemic response.

Evaluation team composition

Although evaluation should ideally be done by independent third party, staff members in the evaluated organization have a significant role to play in the planning, design, implementation and use of this evaluation.

For peer review only - http://bmjopen.bmj.com/site/about/guidelines.xhtml

Timeline

- The evaluation should ideally take place after the first X weeks since the onset of the epidemic. It may also take place immediately after the conclusion of the epidemic

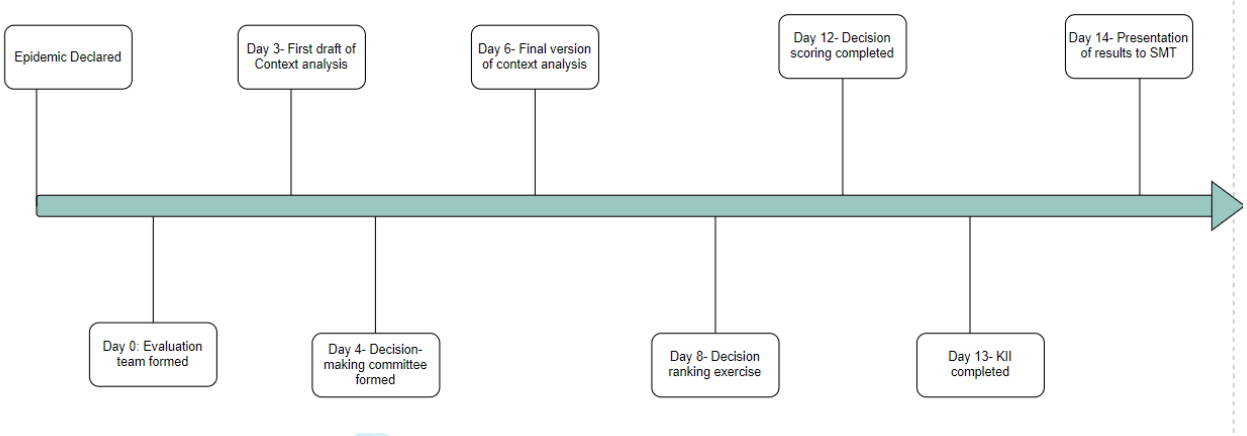

For peer review only

For peer review only - http://bmjopen.bmj.com/site/about/guidelines.xhtml

Section 1- Context analysis

1.1 Purpose

- The purpose of the context analysis is to provide a common base from which to understand the epidemic setting and the actions that have been taken.

1.2 Roles and responsibilities

- The evaluation team lead will lead the exercise and convene the evaluation team to undertake the context analysis.
- The evaluation team will identify principle decision-makers to form decision-making committee

1.3 Procedures

- The evaluation team will undertake a secondary data review in order to produce the first draft of the context analysis
- The review should gather all relevant documentation including:
    - Previous Public Health Situation Analyses (PHSA)
    - Rapid risk assessments (RRA)
    - Response planning documentation
    - Organizational organograms
    - Sitreps
    - Health bulletins
    - Communication products
    - Meeting minutes
- The evaluation team will also identify key internal decision-makers involved in the particular epidemic response who have had significant input in the planning, implementation or evaluation of the epidemic response. These maybe situated within the country office or regional or HQ levels. These may include technical experts, organizational leaders and others
- These decision-makers will be invited to form a committee to contribute to the remaining sections of this evaluation
- The evaluation team lead will circulate the context analysis draft and solicit feedback in a timely manner from the decision-makers
- The evaluation team will consolidate context analysis feedback into a final version

1.4 Outputs

- Consolidated and agreed upon context analysis which summarizes epidemiological description of the epidemic, health resources and services available to the population, health system performance, organizational resources expended in the epidemic response to date, as well as challenges faced in the response

1.5 Associated Documents

See Annex for document checklist, participant list and section 1 of the tool

For peer review only - http://bmjopen.bmj.com/site/about/guidelines.xhtml

Section 2- Decision Selection

2.1 Purpose

The purpose of this section is to produce a unified list of key decisions to evaluate as prelude to evaluate decision-making in the epidemic response.

2.2 Roles and Responsibilities

- The evaluation team lead is responsible for sharing key documentation and producing an initial listing of key decisions
- The decision-making committee is responsible for reviewing, contributing and evaluating the criticality of decisions

2.3 Procedures

- The evaluation team lead will circulate the context analysis and introduce the characteristics for selecting key decisions to the evaluation team.
- In conjunction with the evaluation team, he/she will produce up to 5 key decisions for initial consideration by key decision-making committee identified through the context analysis
- The key decision-makers will review the proposed decisions
- Decision-makers may contribute further decisions for consideration
- Decision-makers will score each of the proposed decisions based on the scoring criteria
- Evaluation team will summarize (take the mean) the score for each decision and select the top 5 decisions using the following formula

2.4 Outputs

- Unified list of no more than 5 key decisions to be further assessed in section 3.

2.5 Associated Documents

See annex for section 2 of the tool

For peer review only - http://bmjopen.bmj.com/site/about/guidelines.xhtml

Section 3- Decision Scoring

3.1 Purpose

The purpose of this section is to ensure that selected critical decisions are evaluated consistently across the assessment criteria and to produce a summary score for the overall epidemic decision-making.

3.2 Roles and responsibilities

- The evaluation team lead is responsible for explaining the evaluation dimensions and criteria
- The evaluation team is responsible for assessing each decision on the criteria

3.3 Procedures

- For each of the selected decisions (maximum 5) the evaluation team will assess whether the necessary decision-making criteria has been met
- They will compile evidence through review of documentation and score the decision on a scale of 1 (no evidence/not met) to 5 (criteria fully met)
- Where there is little or no evidence of a criteria being met, the evaluation team may revert to decision-making committee to provide evidence
- The decision-making committee members are responsible for providing evidence for any specific criteria as requested by the evaluation team
- The evaluation team lead will review the final score for each decision as well compute an overall score for the exercise

3.4 Outputs

- An agreed upon set of scores for each of the critical decisions as well as an overall score

3.5 Associated Documents

Refer to annex for section3 of the tool

For peer review only - http://bmjopen.bmj.com/site/about/guidelines.xhtml

Section 4- Dissemination/ Review

4.1 Purpose

- The purpose of this section is to review and disseminate the findings of the evaluation in order to improve response processes

4.2 Roles and responsibilities

- The evaluation team lead is responsible for compiling the views of organizational members on the overall evaluation process
- The evaluation team lead is responsible for presenting the findings of the evaluation back to the organization
- The organizational leadership is responsible for working with relevant staff members in implementing improvements

4.3 Procedures

- The evaluation team lead should convene staff who have participated in the evaluation and implement focus group discussion (FGD) or key informant interviews(KII) to elicit feedback on the process
- The evaluation team should present the results of both the exercise and participant views to organizational leadership/ Senior Management Team (SMT)in a brief
- The SMT should implement recommendations derived from the exercise

4.4 Outputs

- Identification of decision rated as strong
- Recommendations of decision making dimensions that require strengthening
- Feedback on the utility and feasibility of evaluation
- Identification of areas of the evaluation process that require improvement

4.5 Associated Documents

- See annex for focus group guide and key informant interview guide

For peer review only

For peer review only - http://bmjopen.bmj.com/site/about/guidelines.xhtml

Reference

https://jhumanitarianaction.springeropen.com/articles/10.1186/s41018-020-00068-2#Fn3
https://www.who.int/hac/techguidance/tools/standard_operating_procedures_african_region_en_2014.pdf

https://healthcluster.who.int/docs/librariesprovider16/meeting-reports/updated-phsa-long-form-template-english-june-2019.docx?sfvrsn=81cd6a71_3

For peer review only

For peer review only - http://bmjopen.bmj.com/site/about/guidelines.xhtml

Annex

Document Checklist

No.	Document Type	Details (e.g.)
1	Organogram	*CO organogram March 2021*
2	Meeting Minutes	*Weekly minutes (may 2021-June 2021)*
3		
4		
5		

Evaluation Participants

Evaluation Team	Name (e.g.)	Role
1	*John Doe*	*Team lead*
2	*Pers Indi*	*M&E officer*
3		
Decision-making committee	Name	Role
1	*Fulan*	*Incident manager*
2	*Jama Yulo*	*Operations officer*
3	*Qofa Kale*	*Technical officer*

For peer review only

For peer review only - http://bmjopen.bmj.com/site/about/guidelines.xhtml

Decision Making Evaluation Instrument

Organization:

Date:

Outbreak:

Part A- Summary of Response

(i) Describe briefly the historical, geographic and health context in which the outbreak is occurring *(maximum 250 words).*

For peer review only

(ii) Briefly describe the course of the epidemic from detection up the present focusing on epidemiology, risk factors and effected populations *(maximum 250 words).*

(iii) Describe what actions your organization has taken in response to the epidemic *(maximum 300 words).*

LONDON SCHOOL of HYGIENE &TROPICAL MEDICINE

Part B- Selection of critical decision points

Guide

Considering the following guiding Characteristics, propose 3 -5 critical decision in the response to date. Justify your selection with specific with reference to the characteristics as well as specific examples. A decision does not need to meet all of the following characteristics to be considered critical. These can include decision on beginning, ending or scaling a response, deciding on response options, deciding on target population, as well as deciding on modality of response.

Characteristics of critical decision

I. Consequential- critical decision is consequential and shapes the response to a significant degree
II. Reversibility- a critical decision can be difficult to overturn or reverse at least in the short term)
III. Strategic- entails shift in terms of action taken, resources committed or precedent set
IV. Uncertainty –Wide range of uncertainty/ complex array of options
V. Reputionally Risky- Entails a high level of organisational reputational risk

Decision 1

Description
Describe the decision

Rationale
Justify your proposed decision by selecting the relevant Characteristics

☐ Characteristic 1

☐ Characteristic 2

☐ Characteristic 3

☐ Characteristic 4

☐ Characteristic 5

Narrative
Describe any other reason for considering this decision critical (maximum 200 words)

Decision 2

For peer review only - http://bmjopen.bmj.com/site/about/guidelines.xhtml

Description
Describe the decision

Rationale
Justify your proposed decision by selecting the relevant Characteristics

☐ Characteristic 1

☐ Characteristic 2

☐ Characteristic 3

☐ Characteristic 4

☐ Characteristic 5

Narrative
Describe any other reason for considering this decision critical (maximum 200 words)

Decision 3

Description
Describe the decision

Rationale
Justify your proposed decision by selecting the relevant Characteristics

☐ Characteristic 1

☐ Characteristic 2

☐ Characteristic 3

☐ Characteristic 4

☐ Characteristic 5

Narrative
Describe any other reason for considering this decision critical (maximum 200 words)

For peer review only - http://bmjopen.bmj.com/site/about/guidelines.xhtml

LONDON SCHOOL of HYGIENE & TROPICAL MEDICINE

For peer review only

For peer review only - http://bmjopen.bmj.com/site/about/guidelines.xhtml

Part C-Decision-making criteria

For each selected decision in Part B, complete the following scoring exercise, keeping in mind to provide the necessary evidence.

Decision: __*e.g. Decision to conduct mobile vaccinations in region Y in May 2020*

Transparency

To what degree were the following criteria met?

Criteria	Fulfilment of criteria<br>*To what extent was the criteria fulfilled in this particular decision*	Evidence of fulfilment<br>*Provide reference to specific sources to support your assessment*
Inclusivity[1]	5- Very Strong evidence	*e.g. Refer to meeting minutes March 24, 2020 as well as response plan April 2020. These show presence of diverse stakeholders in this decision from all departments as well as MoH*
Usage of explicit decision-making criteria[2]	Choose an item.	
Following clear process or method[3]	Choose an item.	
Usage of mechanism to publicise rationale [4]	Choose an item.	

Guide

1. Consider who was involved. Was there heterogeneity in ranks and roles?

2. Were the goals and objectives clearly pre-specified or were decisions rationalized after the fact?

For peer review only - http://bmjopen.bmj.com/site/about/guidelines.xhtml

LONDON SCHOOL of HYGIENE &TROPICAL MEDICINE

3. Were there documented priority setting process and/or use of priority setting framework? Was there a decision-tree

4. Was there clear documentation on decisions reached?

For each selected decision in Part B, complete the following scoring exercise, keeping in mind to provide the necessary evidence.

Decision: ____

Contestability

To what degree was the following criteria met?

Criteria	Fulfilment of criteria<br>*To what extent was the criteria fulfilled in this particular decision*	Evidence of fulfilment<br>*Provide reference to specific sources to support your assessment*
Opportunity for revision[5]	Choose an item.	
Was the decision devolved?[6]	Choose an item.	

Accountability

To what degree was the following criteria met?

Criteria	Fulfilment of criteria<br>*To what extent was the criteria fulfilled in this particular decision*	Evidence of fulfilment<br>*Provide reference to specific sources to support your assessment*
Engagement with effected communities[7]	Choose an item.	

For peer review only - http://bmjopen.bmj.com/site/about/guidelines.xhtml

Rigour

Criteria	Fulfilment of criteria *To what extent was the criteria fulfilled in this particular decision*	Evidence of fulfilment *Provide reference to specific sources to support your assessment*
Explicit outcome[8]	Choose an item.	
Feasible outcome[9]	Choose an item.	
Strengthen healthcare system[10]	Choose an item.	
Evidence based [11]	Choose an item.	

Guide

5. Was there any scope to revise and overturn a decision? How open was the discussion? Were alternatives debated? What were they? Was consensus achieved and if so, how was this reached?

6. What role did respondents in closest proximity to the epidemic (e.g. subnational level) or local technical experts play? What role did organizational leadership play? Who was the most junior decision-maker? Who was the most senior?

7. To what degree were the effected communities involved in the response? Who was involved from the effected community? Were they informed of the response activities? Did this notice make any difference for those notified?

8. Were intended outcomes of the decision clearly articulated? Were targets set? What were they?

9. Were alternatives debated? What were they?

10. Was there evidence that this decision was in-line with wider strategy? What pillar of the health system were targeted?

For peer review only - http://bmjopen.bmj.com/site/about/guidelines.xhtml

11. Was "Strong public health rationale and solid scientific information provided to justify health measures?" What evidence was utilized? What evidence was missing?

Part D- Summary Decision-making Score

Scoring formula

For each assessed decision, utilize the formulae below to populate the table

Transparency score = average of criteria 1-4

Contestability= average of criteria 5-6

Accountability= score of criteria 7

Rigour= average of criteria 8-11

Dimension	Average	Score
Transparency		
Contestability		
Rigour		
Accountability		

For peer review only - http://bmjopen.bmj.com/site/about/guidelines.xhtml

LONDON SCHOOL of HYGIENE & TROPICAL MEDICINE

Recommendations

Based on the results above, briefly describe what specific steps you recommend in order to improve the decision-making process in this epidemic *(maximum 300 words).*

For peer review only - http://bmjopen.bmj.com/site/about/guidelines.xhtml

For peer review only

For peer review only - http://bmjopen.bmj.com/site/about/guidelines.xhtml

LONDON SCHOOL of HYGIENE &TROPICAL MEDICINE

For peer review only

For peer review only - http://bmjopen.bmj.com/site/about/guidelines.xhtml

LONDON SCHOOL of HYGIENE &TROPICAL MEDICINE

Decision-making Scorecard

WHO Somalia's COVID-19 response

Evaluation study

Nov 22nd- December 8th 2021

Abdihamid Warsame

Faculty of Epidemiology and Population health

London School of Hygiene and Tropical Medicine

For peer review only

Critical Decisions

Participants were advised to select important decisions for critical evaluation based on the following criteria on what constitutes a critical decision.

Characteristics

A critical decision is one which has one or more of the following characteristics:

I. Consequentiality-: A critical decision is consequential and shapes the response to a significant degree
II. Reversibility-: A critical decision can be difficult to overturn or reverse at least in the short term
III. Strategic: A critical decision entails a significant shift in terms of action taken, resources committed or precedent set
IV. Uncertainty: A critical decision entails a wide range of uncertainty or complex array of options
V. Reputational Risk: A critical decision entails a high level of organisational reputational risk

Decision	Criteria for Selection
i. Formation of Rapid Response Teams	☒ Characteristic 1<br>☐ Characteristic 2<br>☒ Characteristic 3<br>☒ Characteristic 4<br>☒ Characteristic 5
ii. Establishment of 3 strategic PCR labs	☒ Characteristic 1<br>☒ Characteristic 2<br>☒ Characteristic 3<br>☒ Characteristic 4<br>☒ Characteristic 5
iii. Launch of Incident Management System	☒ Characteristic 1<br>☐ Characteristic 2<br>☒ Characteristic 3<br>☒ Characteristic 4<br>☒ Characteristic 5
iv. Focus on case management through scale up of Oxygen	☒ Characteristic 1<br>☐ Characteristic 2<br>☒ Characteristic 3<br>☒ Characteristic 4<br>☒ Characteristic 5

For peer review only

Summary Decision Scorecard

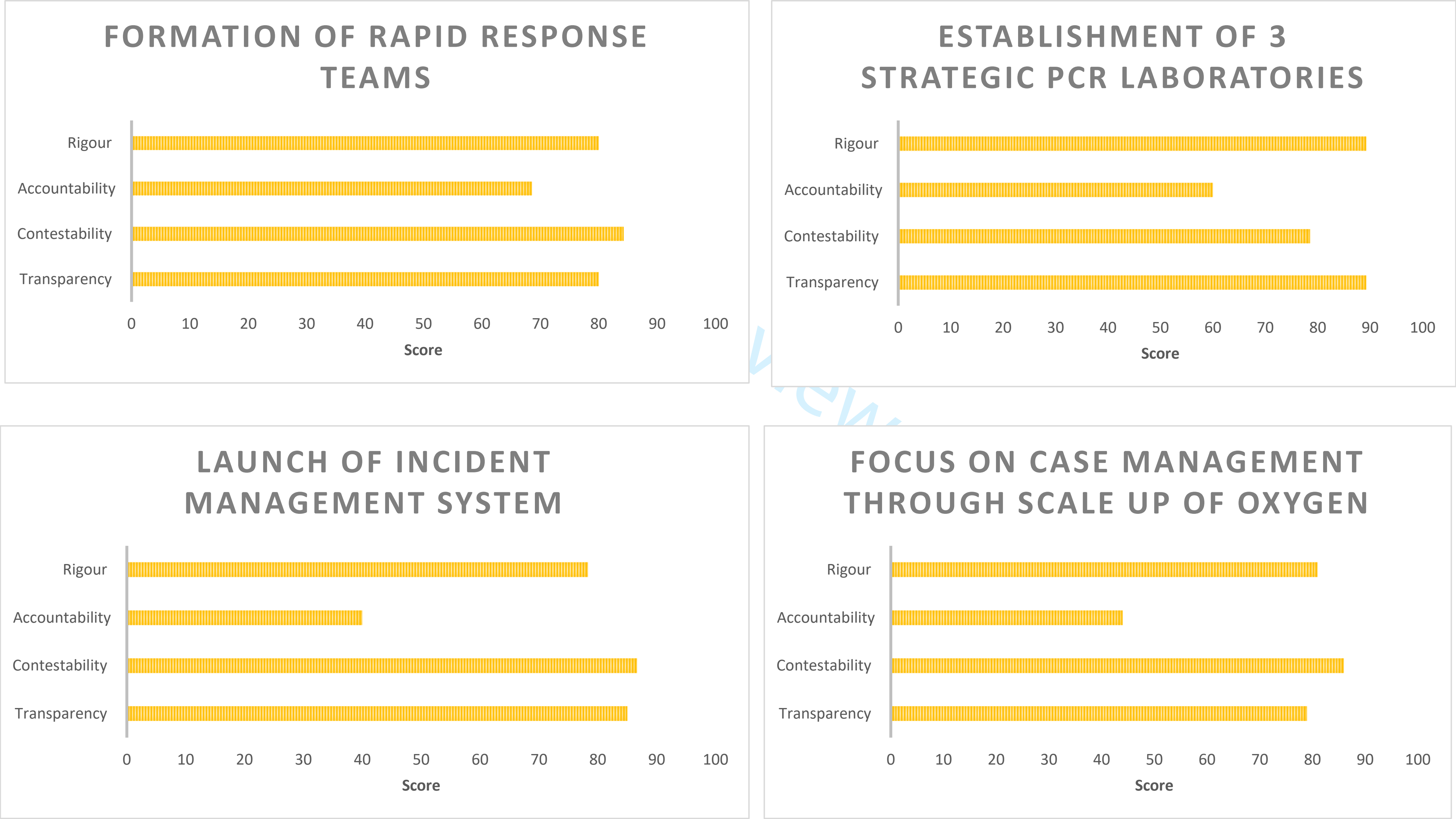

Detailed Scores & Interpretation

i. Formation of Rapid Response Teams

The Rapid Response Teams (RRT) and Community Health Workers (CHW) were recruited early in the COVID-19 response in order to strengthen and expand the surveillance capacity in high priority districts. A decision was undertaken to train and equip members of the RRTs and CHWs for case detection, notification, testing and contact tracing.

Inclusivity 86%	Decision criteria 74%	Process 77%	Public rationale 83%

Participants scored the transparency of this decision as high citing existing documentation such as response plan, IMST meeting minutes and proposal documents. They stated that there was strong evidence of inclusive decision-making as the decision included WHO staff from all programs as well as program managers. They also stated that some of the decision-makers were assigned to liaise/coordinate with the stakeholders including MoH and other partners and discuss the decision with them and publicise the rationale. They also noted following clear method for arriving at this decision including utilizing risk assessments. Participants referenced the usage of COVID-19 transmission classification for Somalia drawn from the interim guidelines by WHO for considerations for implementing and adjusting public health and social measures in the context of COVID-19 as providing clear criteria for this decision.

Devolution 74%	Revision 94%	Community engagement 69%	Explicit outcome 77%

Contestability was the highest scored dimension. Participants noted there were sufficient opportunities to revise the decision and cited the inter-action review of November 2020 as evidence. Additionally, the decision was discussed in various IMST meeting with document shared and commented, feedbacks incorporated into the final decision. The MoH was also said to be involved in the selection of RRTs and CHWs, implementation and modification of the structure and role and responsibilities. They also noted that revision of this decision resulted in expansion of the RRT mechanism to further district. Participants also noted that the IMST structure allowed for devolution in the decision-making in which decision-making was shared amongst senior decision-makers (country level IMST team members under the leadership of the WR) and junior decision makers (state level and district level staff)

In terms of accountability, this was scored lower as there was difference of scoring amongst participants as to the degree of engagement with the effected communities. Some participants stated that there was strong engagement with communities in implementing the RRT strategy but limited engagement in the decision to use this mechanism.

Feasibility 88%	Strengthen Health system 74%	Evidence based 77%

Participants noted this decision was taken with a high degree of rigour. In particular, they noted that strengthening healthcare system was among the objectives. Furthermore, they noted that the outcomes of this decision were explicitly articulated and include bridging the gap between health facilities and communities. They also noted that feasibility criteria were considered to be high due to staffing the RRT via the existing network of CHWs. Lastly they noted that targets were set and tracked through an interactive dashboard.

ii. Establishment of 3 strategic PCR labs

At the outset of the pandemic in Somalia, there did not exist national capacity to test samples for COVID-19. Responders relied in the first weeks, upon sending samples for testing abroad which were found to be time consuming and expensive. The lack of national labs to undertake this critical function was found to be a gap which required urgent attention. As a result, the decision was taken to establish three strategic PCR capable laboratories in Mogadishu, Garowe and Hargeisa.

Inclusivity 91%	Decision criteria 89%	Process 89%	Public rationale 89%

The transparency score is comprised of 4 criteria. In terms of inclusivity, participants scored this decision highly. terms of transparency. The decision was taken in conjunction with the MoH and UN partners. The rationale behind this decision was said to have been shared in internal coordination meetings as well as external coordination meetings with partners. A number of documents were cited as evidence including WHO Somalia Technical Program Update Quarterly, IMST meeting minutes, CERF Project Action Plan and WCO FCDO report.

Devolution 65%	Revision 91%	Community engagement 60%	Explicit outcome 91%

In terms of contestability, this decision was reviewed in the initial proposal development meeting with the MoH in which there was opportunity for revision. Additionally, participants stated that a review of the decision prompted further procurement of PCR machines and scaling up of PCR testing capacity to remaining states.

Participants expressed mixed scoring on the accountability dimension. Some stated that there was high accountability through interaction with state level MoH while others expressed that there was limited/no consultation with communities in these 3 cities regarding the establishment of PCR capacity in these labs. Some participants stated this criterion was inapplicable as the labs were pre-existing and upgrading did not require further engagement.

Feasibility 91%	Strengthen Health system 89%	Evidence based 89%

Participants scored this decision highly in terms of scientific rigour. They stated that the usage of PCR is global standard to detect COVID-19. Additionally, they stated the decision was built on evidence extracted from a previous 2019 influenza testing lab assessment which provided rationale and process for the decision to set up these labs for COVID-19 testing. Participants also cited the National Action Plan for Health Security as providing evidence for this decision. The decision was also found to have satisfied the strengthened the health system criteria as the establishment of these 3 labs allowed for testing beyond COVID-19.

For peer review only

iii. Launch of Incident Management System

As part of efforts to enhance the effectiveness of the Health Emergencies response and in line with the Emergency response framework, WHO Somalia launched the Incident Management Support Team to coordinate and guide the response to the COVID-19 pandemic within Somalia.

Inclusivity 90%	Decision criteria 83%	Process 80%	Public rationale 87%

Participants scored the inclusivity of this decision as high as they stated this decision was taken after a series of consultation with staff led by the WR. These deliberations were captured in meeting minutes and which detailed the process and rationale. The final decision was communicated by WR to all staff through an office memo. Participants also noted the engagement with the MoH in the formation of the IMST system as evidence of inclusivity.

Devolution 80%	Revision 93%	Community engagement 40%	Explicit outcome 77%

In terms of revision, participants noted that there was some opportunity which resulted in the change of incident managers but not in the structure itself. With regards to accountability, participants noted limited to no engagement with effected communities. However other participants stated that parallel state and district-level IMST did engage with the local community. While these lower level IMST did not feed into the formation of the national IMST, they maintain a certain level autonomy.

Feasibility 83%	Strengthen Health system 80%	Evidence based 73%

Participants cited a number of routine information products as evidence of decision outcomes and feasibility considerations. They cited COVID-19 Response plan and action trackers as evidence for the articulation of clear outcomes and evidence based targets. They also cited this response plan which articulated heath system strengthening as an explicit goal and an important pillar in the IMST approach. Participants also cited the WHO Global guidance on strategic response to C19 as providing evidence to support this decision.

iv. Focus on case management through scale up of Oxygen

Due to the presentation of symptoms and the aetiology of the disease as well as gaps in case management capacity, WHO Somalia undertook concerted effort to scale up oxygen therapy across the country in order to improve outcomes.

Inclusivity 76%	Decision criteria 76%	Process 76%	Public rationale 88%

The decision-making was scored as somewhat inclusive by participants. They cited the IMST meeting minutes in June 2020 as evidence as well as the COVID-19 response plan which detailed the discussion and decision criteria. They noted close deliberation with the case management focal persons within the MoH. Participants also noted that the rationale was communicated clearly through specific information products and web stories on WHO and other websites.

For peer review only

Devolution 84%	Revision 88%	Community engagement 44%	Explicit outcome 76%

Participants noted close discussion with federal member states on the modality of scale up as evidence of devolved decision-making. Participants mentioned that there were several opportunities to revise this decision in which the need for oxygen provision was reaffirmed but the modality was revised. They noted that the initial decision was for WHO to support procurement of oxygen locally. This changed to procurement of cylinders and ventilators and eventually to supporting the establishment of oxygen plants. Participants mentioned limited evidence of community engagement in the decision itself but significant engagement through health messaging after the fact. They cited dashboards as containing evidence of clear outcomes and targets that underlay this decision.

Feasibility 80%	Strengthen Health system 84%	Evidence based 84%

Participants noted that this decision supported strengthening of the health system by fortifying performance of isolation facilities and optimizing case management. They also cited later documents as providing evidence that underlay this decision such as the technical update on survival among hospitalized COVID-19 patients.

Recommendations

A number of recommendations were put forward by participants in order to strengthen the decision-making process within the organisation. They include the following:

- Improve documentation on decision-making deliberations as participants were aware of many discussions that were not reflected in official internal or external records.
- To further strengthen decision mechanisms by clearly outline who is involved, their responsibilities and the mechanisms by which they arrived at the decision.
- Strengthen engagement with affected communities in addition to government officials *during* the decision-making process rather than solely communicating finalised decisions. This will strengthen accountability.
- Consider sustainability as a potential criterion for good decision-making
- Repeat this decision-making exercise on a more routine basis by adopting it as a component of internal evaluation

For peer review only

Annex

Score Criteria

Dimension	Criteria	Description
Transparency	Inclusivity	The extent to which the process was inclusive, reflected in heterogeneity in rank and roles amongst decision makers involved.
	Use of explicit decision-making criteria	The extent to which the goals and objectives of the decision were clearly pre-specified. The absence of post-decision rationalization.
	Following clear process or method	The extent to which a priority setting process was in place, reflected in demonstrated use of priority setting frameworks, decision trees or other mechanism.
	Use of mechanism to publicise rationale	The extent to which clear documentation on the decision exists as well as the method used to communicate decisions.
Contestability	Opportunity for revision	The extent to which there existed scope to revise and overturn a decision including the debating of alternatives and description of how consensus was reached.
	Was the decision devolved?	The degree to which participants in closest proximity to the epidemic (e.g. subnational level) or local technical experts participate in the decision, including consideration of rank.
Accountability	Engagement with affected communities	The degree to which affected communities were involved in the response decision-making including at a minimum whether they were informed of the response activities and what effect this notification had on the communities.
Rigour	Explicit outcome	The extent to which intended outcomes of the decision were clearly articulated, including through setting of targets.
	Feasible outcome	The extent to which feasibility was considered in decision-making including debating of alternatives.
	Strengthens healthcare system	The extent to which the decision was in-line with wider strategy including the strengthening of the health system
	Evidence based	The extent to which the decision was based on strong public health rationale and robust scientific information.

For peer review only

Decision-making audit scorecard

CARE Somalia's COVID-19 response

Evaluation study

November 8th -Nov 22nd2021

Abdihamid Warsame

Faculty of Epidemiology and Population health

London School of Hygiene and Tropical Medicine

21 January 2022

For peer review only

Background

On the 30th of January 2020, the World Health Organization (WHO) declared the emergence of SARS-CoV-2 corona virus as a Public Health Emergency of International Concern (PHEIC) because of evident human-to-human transmission and renamed it COVID-19. On the 16th of March 2020, Somalia reported its 1st COVID- 19 confirmed case, a Somali national with travel history to China. As of the 20th of January 2022, Somalia had 24,261 confirmed cases of COVID-19 with 1335 deaths. The Federal Ministry of Health launched the National COVID-19 Preparedness & Response Plan on the 26th March 2020 with a budget of USD 57 million and estimated caseload of 6,000 confirmed cases.

Somalia's capacity to prevent, detect, and respond to any global health security threat scored six out of 100 on the 2016 Health Emergency Preparedness Index. Poor urban communities, internally displaced persons(IDPs), the elderly, households of minority clans, riverine communities, women and child headed households, and children are most at risk due to social exclusion and decreased accessibility to health services, sanitation and water.

The COVID-19 pandemic weakened Somalia's already fragile health system that has been negatively impacted by protracted conflict, cyclic drought, and locust infestation. The Somali health system has not been able to cope adequately with the demands of a COVID-19 response with poor and few testing facilities, low capacity of health care workers and poor funding. Social cultural practices, rumours, misconceptions, poor hygiene practices and communal caring for the sick in homes and communities, have additionally amplified the spread of the virus.

Methods

The evaluation occurred in Garowe, Somalia from 8- 18th of November 2021. A total of 8 participants from various units within CARE Somalia took place in this evaluation.

The evaluation was undertaken following the standard operating procedures (SOP) developed by the LSHTM research team (See annex) and with the appointment of a CARE focal point. The CARE focal point gathered the necessary background documentation as well as supported the formation of the decision-making committee through recruitment of evaluation participants from the organization. The focal point completed the first draft of the contextual analysis as well as consolidated input from the other participants. He was also instrumental in facilitating the group work sessions.

For peer review only - http://bmjopen.bmj.com/site/about/guidelines.xhtml

Critical Decisions

Participants were advised to select important decisions for critical evaluation based on the following criteria on what constitutes a critical decision.

Characteristics

A critical decision is one has one or more of the following characteristics:

I. Consequentiality-: A critical decision is consequential and shapes the response to a significant degree
II. Reversibility-: A critical decision can be difficult to overturn or reverse at least in the short term
III. Strategic: A critical decision entails a significant shift in terms of action taken, resources committed or precedent set
IV. Uncertainty: A critical decision entails a wide range of uncertainty or complex array of options
V. Reputational Risk: A critical decision entails a high level of organisational reputational risk

Decision	Criteria for Selection
i. Adaptation of nutrition programming to COVID-19	☐ Characteristic 1<br>☒ Characteristic 2<br>☒ Characteristic 3<br>☐ Characteristic 4<br>☐ Characteristic 5
ii. Closure of CARE offices and restriction of staff movement	☒ Characteristic 1<br>☐ Characteristic 2<br>☒ Characteristic 3<br>☐ Characteristic 4<br>☒ Characteristic 5
iii. Implementation of COVID-19 project in Sool and Sanaag	☒ Characteristic 1<br>☒ Characteristic 2<br>☒ Characteristic 3<br>☒ Characteristic 4<br>☐ Characteristic 5

For peer review only - http://bmjopen.bmj.com/site/about/guidelines.xhtml

Summary Decision Scorecard

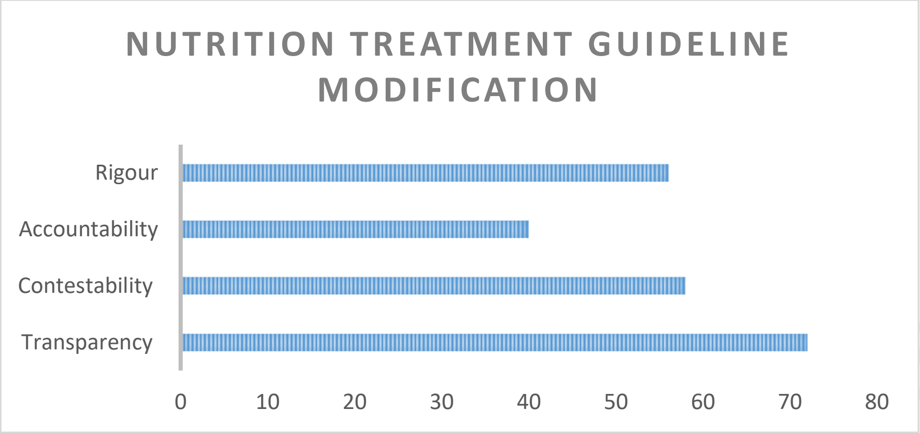

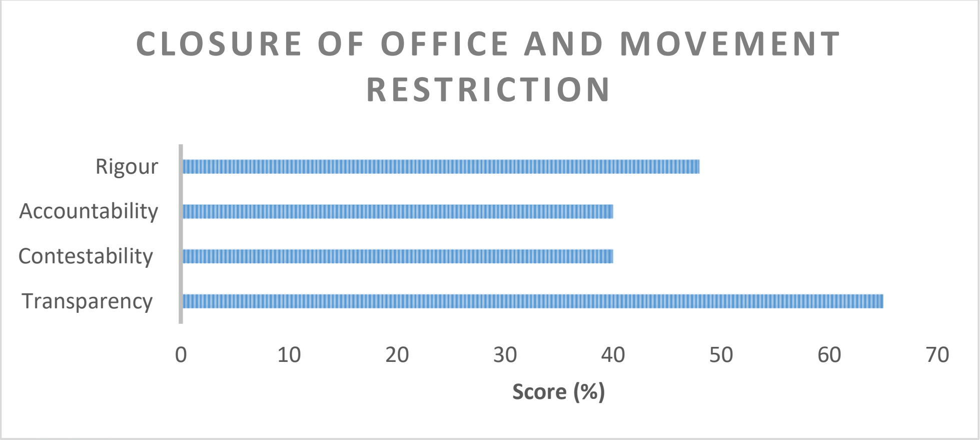

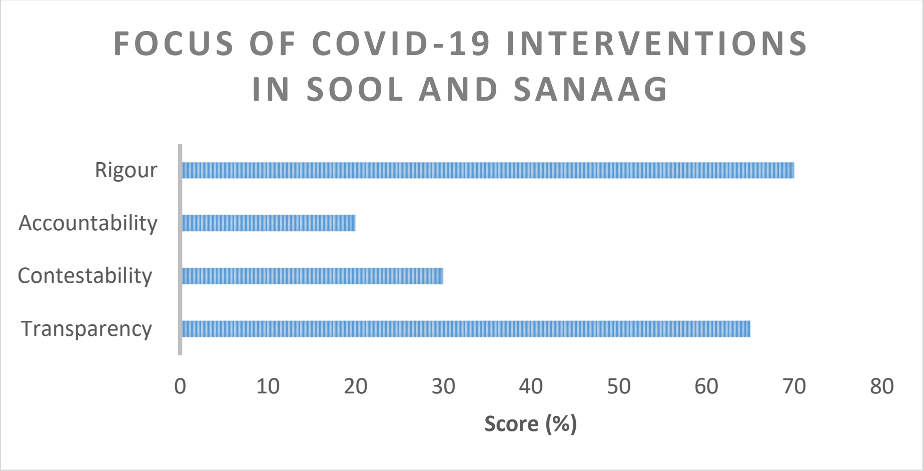

For peer review only - http://bmjopen.bmj.com/site/about/guidelines.xhtml

LONDON SCHOOL of HYGIENE &TROPICAL MEDICINE

Detailed Scores & Interpretation

1. Nutrition treatment guideline modification

Inclusivity 72%	Decision criteria 76%	Process 64%	Public rationale 76%

In terms of transparency, the decision to modify nutrition programme guidelines to be COVID-19 compatible was found to be fairly transparent. However, participants described a deficit in accountability as these modifications were reportedly made at the cluster level without the input of all partners. Additionally, participants reported that the affected communities were not consulted and could not feed into this decision-making process.

Devolution 44%	Revision 72%	Community engagement 40%	Explicit outcome 56%

Moreover, participants noted they did not have opportunity to contest these adaptations before they were adopted. They did however agree that there was a clear rationale to minimize transmission risk to the population. Nevertheless, participants did make clear the missed opportunity in engaging with the community to prepare them for these modifications.

Feasibility 55%	Strengthen Health system 60%	Evidence based 53%

Participants expressed concern that these adaptations would reduce patient follow-up, risk missing eligible program beneficiaries and could lower the effectiveness of the program.

2. Closure of Office and movement restriction

Inclusivity 60%	Decision criteria 76%	Process 64%	Public rationale 60%

The closure of CARE offices and restriction of staff movement was done early in the response. The decision was taken by the senior management and communicated via email to all staff. As a result, evaluation participants felt that this approach lessened the transparency as an opportunity was not provided to staff to feed into this decision. There was no clear documentation provided on the deliberations of this decision and the various options considered.

Participants did concede that it was possible that there were prioritization and decision-frameworks utilized but they were not disseminated to all staff.

Feasibility 60%	Strengthen Health system 40%	Evidence based 36%

Participants noted that in terms of supporting evidence, WHO and other international guidelines were possibly followed. No specific document was cited. However, the driving factor was to preserve business continuity and an abundance of caution as there were limited evacuation options for staff who felt ill.

Devolution 36%	Revision 44%	Community engagement 40%	Explicit outcome 60%

Participants mentioned that the decision to close offices was not devolved to sub-offices but was made at the country level. Additionally, there was limited community engagement with participants noting that as this could possibly affect program delivery, there should have been some level of community engagement.

For peer review only - http://bmjopen.bmj.com/site/about/guidelines.xhtml

LONDON SCHOOL of HYGIENE &TROPICAL MEDICINE

3. Focus of COVID-19 Interventions in Sool and Sanaag

Inclusivity 40%	Decision criteria 80%	Process 80%	Public rationale 60%

CARE has largely been conducting its humanitarian and development programming in rural areas prior to the pandemic. When the pandemic occurred, fundraising and response resources were focused to areas which CARE had a strong field presence. These were in Sool and Sanaag regions in largely rural areas. However, the majority of COVID-19 cases were in urban centres and in higher density areas of the country. Thus there was a mismatch between where resources were directed and where they were most required. Participants explained that as a result of this prioritization of existing programme areas, there was inadequate strategic focus and limited contestability in the decision.

Devolution 40%	Revision 20%	Community engagement 20%	Explicit outcome 60%

Participants reported limited community engagement. However, there was ample opportunities to revise this decision according to some participants. There was also sufficient devolution with the design of the COVID-19 intervention done by both senior management as well as some technical staff based in the sub-offices.

Feasibility 80%	Strengthen Health system 80%	Evidence based 60%

Participants did not feel that this decision strengthened the health system as much as it could have if resources were more precisely directed where needed. They did cite usage of early available WHO guidelines as having a role in how the decision was undertaken. Participants rated the feasibility criteria as high based on the consideration by decision-makers to implement the COVID-19 interventions in existing program areas.

Conclusion

Overall very few primary or secondary documents were specifically cited by participants as evidence for the decision-making criteria. Participants were aware to varying degrees that some documents such as minutes and guidelines did influence decisions but were largely unable to cite them for this evaluation. They instead relied primarily on their recollection of events and discussions with fellow participants to piece together the events that transpired.

For peer review only - http://bmjopen.bmj.com/site/about/guidelines.xhtml

LONDON SCHOOL of HYGIENE &TROPICAL MEDICINE

Recommendations

Decision-making

In order to improve the quality of decision-making in CARE's COVID-19 response and more broadly in its humanitarian responses, the following recommendations have been put forward:

1) Improve the inclusivity of key critical decision-making through expanding the membership of decision-makers to include junior staff and field staff.
2) Improve trust and buy-in of decisions through strengthening reporting of decision rationale and criteria.
3) Strengthen documentation of response activities to allow for more in-depth real-time or post response evaluation.
4) Consider more critically the relevance of scientific evidence and public health measures to the context rather than blanket adoption.
5) Allow for more contextually specific factors to shape decisions rather than blanket decisions

Evaluation Process

1) Allow for more time pre-evaluation in order for evaluation team to orient themselves with the methodology.
2) Translate evaluation tools and documentations into local language to allow for better understanding and involvement of evaluation participants
3) Allow for remote or asynchronous completion of evaluation tools by participants
4) Broaden the evaluation participants to include non-emergency staff and representatives of the community and local government
5) Consider practise scenario to allow for better uptake by evaluation team prior to commencing evaluation

For peer review only

For peer review only - http://bmjopen.bmj.com/site/about/guidelines.xhtml

LONDON SCHOOL of HYGIENE &TROPICAL MEDICINE

Annex

Evaluation Schedule

Step	Action	Responsible	Date
1	Recruitment of evaluation team and signing of consent forms	All participants	November 8th
2	First Part A- Draft of Context Analysis	CARE Evaluation Focal Person	November 11
3	Review of Part A- Context Analysis	All Participants	November 13th
4	Set up Meeting	LSHTM & CARE focal person	November 14th
6	Completion of Part B- Decision Selection	All participants	November 16
7	Decision Ranking Exercise	All Participants as group	November 16
8	Completion of Part C- Scoring of Decisions	All Participants separately	November 17
8	Completion of Part D- Overall Scoring	LSHTM	November 25th
9	Key Informant Interview	All Participants	November 18- 21st

For peer review only - http://bmjopen.bmj.com/site/about/guidelines.xhtml

Score Criteria

Dimension	Criteria	Description
Transparency	Inclusivity	The extent to which the process was inclusive, reflected in heterogeneity in rank and roles amongst decision makers involved.
	Use of explicit decision-making criteria	The extent to which the goals and objectives of the decision were clearly pre-specified. The absence of post-decision rationalization.
	Following clear process or method	The extent to which a priority setting process was in place, reflected in demonstrated use of priority setting frameworks, decision trees or other mechanism.
	Use of mechanism to publicise rationale	The extent to which clear documentation on the decision exists as well as the method used to communicate decisions.
Contestability	Opportunity for revision	The extent to which there existed scope to revise and overturn a decision including the debating of alternatives and description of how consensus was reached.
	Was the decision devolved?	The degree to which participants in closest proximity to the epidemic (e.g. subnational level) or local technical experts participate in the decision, including consideration of rank.
Accountability	Engagement with affected communities	The degree to which affected communities were involved in the response decision-making including at a minimum whether they were informed of the response activities and what effect this notification had on the communities.
Rigour	Explicit outcome	The extent to which intended outcomes of the decision were clearly articulated, including through setting of targets.
	Feasible outcome	The extent to which feasibility was considered in decision-making including debating of alternatives.
	Strengthens healthcare system	The extent to which the decision was in-line with wider strategy including the strengthening of the health system
	Evidence based	The extent to which the decision was based on strong public health rationale and robust scientific information.

For peer review only - http://bmjopen.bmj.com/site/about/guidelines.xhtml

No.
S1
S2
S3
S4
S5
S6
S7
S8
S9
S10
S11
S12
S13
S14
S15
S16
S17
S18
S19
S20
S21

For peer review only

Requirement
Title
Abstract
Problem Formulation
Purpose or research question
Qualitative approach and research paradigm
Researcher characteristics and reflexivity
Context
Sampling strategy
Ethical issues pertaining to human subjects
Data collection methods
Data collection instruments and technologies
Units of study
Data processing
Data analysis
Techniques to enhance trustworthiness
Synthesis and Interpretation
Links to empirical data
Integration with prior work, implication, transferability and contributions to the field
Limitations
Conflicts of Interest
Funding

For peer review only

Location in manuscript
Page 1
Page 2
page 2 lines
Page 3
page 9
page 8
Page 7
page 6
page 20
pages 6-8
page 4 and supplementary material
pages 7-8
page 8
page 9
page 4 and supplementary material (publication of audit tools and reports)
pages 9-16
pages 11-16
pages 4, 16-18
page 18
Page 20
Page 20

For peer review only

BMJ Open

Auditing the quality of epidemic decision-making in Somalia: a pilot evaluation

Journal:	*BMJ Open*
Manuscript ID	bmjopen-2022-065122.R2
Article Type:	Original research
Date Submitted by the Author:	19-Dec-2022

Complete List of Authors:	Warsame, Abdihamid; London School of Hygiene and Tropical Medicine Department of Infectious Disease Epidemiology,<br>Ore, Abdulkadir; Care Somalia<br>Azad, Abdullah; World Health Organization Somalia<br>Hassan, Farhan; World Health Organization Somalia<br>Blanchet, Karl; University of Geneva Faculty of Medicine, Geneva Centre of Humanitarian Studies<br>Palmer, Jennifer; London School of Hygiene and Tropical Medicine Department of Global Health and Development,<br>Checchi, Francesco; London School of Hygiene and Tropical Medicine Faculty of Epidemiology and Public Health

**Primary Subject Heading**:	Global health
Secondary Subject Heading:	Public health, Qualitative research, Infectious diseases
Keywords:	Health policy < HEALTH SERVICES ADMINISTRATION & MANAGEMENT, International health services < HEALTH SERVICES ADMINISTRATION & MANAGEMENT, Organisational development < HEALTH SERVICES ADMINISTRATION & MANAGEMENT, Quality in health care < HEALTH SERVICES ADMINISTRATION & MANAGEMENT, Protocols & guidelines < HEALTH SERVICES ADMINISTRATION & MANAGEMENT, INFECTIOUS DISEASES

SCHOLARONE™
Manuscripts

I, the Submitting Author has the right to grant and does grant on behalf of all authors of the Work (as defined in the below author licence), an exclusive licence and/or a non-exclusive licence for contributions from authors who are: i) UK Crown employees; ii) where BMJ has agreed a CC-BY licence shall apply, and/or iii) in accordance with the terms applicable for US Federal Government officers or employees acting as part of their official duties; on a worldwide, perpetual, irrevocable, royalty-free basis to BMJ Publishing Group Ltd ("BMJ") its licensees and where the relevant Journal is co-owned by BMJ to the co-owners of the Journal, to publish the Work in this journal and any other BMJ products and to exploit all rights, as set out in our licence.

The Submitting Author accepts and understands that any supply made under these terms is made by BMJ to the Submitting Author unless you are acting as an employee on behalf of your employer or a postgraduate student of an affiliated institution which is paying any applicable article publishing charge ("APC") for Open Access articles. Where the Submitting Author wishes to make the Work available on an Open Access basis (and intends to pay the relevant APC), the terms of reuse of such Open Access shall be governed by a Creative Commons licence – details of these licences and which Creative Commons licence will apply to this Work are set out in our licence referred to above.

Other than as permitted in any relevant BMJ Author's Self Archiving Policies, I confirm this Work has not been accepted for publication elsewhere, is not being considered for publication elsewhere and does not duplicate material already published. I confirm all authors consent to publication of this Work and authorise the granting of this licence.

For peer review only

Auditing the quality of epidemic decision-making in Somalia: a pilot evaluation

Abdihamid Warsame[1]
Abdikadir Ore[2]
Abdullah Azad[3]
Farhan Hassan[3]
Karl Blanchet[4]
Jennifer Palmer[5]
Francesco Checchi[1]

[1]Faculty of Epidemiology and Population Health, The London School of Hygiene & Tropical Medicine, London, United Kingdom.

[2]CARE International Somalia. Garowe, Somalia

[3] Health Emergencies Unit. World Health Organization Somalia. Mogadishu, Somalia

[4]Geneva Centre of Humanitarian Studies, Faculty of Medicine, University of Geneva, Geneva, Switzerland

[5]Faculty of Public Health and Policy, The London School of Hygiene & Tropical Medicine, London, United Kingdom.

Corresponding author:

Abdihamid Warsame

Faculty of Epidemiology and Population Health, The London School of Hygiene & Tropical Medicine, London, United Kingdom.

Email address: Abdihamid.warsame@lshtm.ac.uk

For peer review only

Abstract

Objective

To assess decision making quality through piloting an audit tool among decision makers responding to the COVID-19 epidemic in Somalia.

Design and Setting

We utilized a mixed methods programme evaluation design comprising quantitative and qualitative methods. Decision-makers in Somalia piloted the audit tool generating a scorecard for decision-making in epidemic response. They also participated in key informant interviews discussing their experience with the audit process and results.

Participants

A total of 18 decision-makers from two humanitarian agencies responding to COVID-19 in Somalia were recruited to pilot the audit tool.

Outcome measures and analysis

We used thematic analysis to assess the feasibility and perceived utility of the audit tool by intended users (decision-makers). We also calculated Fleiss' Kappa to assess interrater agreement in the audit scorecard.

Results

The audit highlighted areas of improvement in decision-making amongst both organisations including in the dimensions of accountability and transparency. Despite the audit occurring in a highly complex operating environment, decision-makers found the process to be feasible and of high utility. The flexibility of the audit approach allowed for organisations to adapt the audit to their needs. As a result, organisation reported a high level of acceptance of the findings

Conclusion

Strengthening decision-making processes is key to realizing the objectives of epidemic response. This pilot evaluation contributes towards this goal by the testing what, to our knowledge, may be the first tool designed specifically to assess quality of decision-making processes in epidemic response. The tool has proven feasible and acceptable in assessing decision-making quality in an ongoing response and has potential applicability in assessing decision-making in broader humanitarian response.

Strengths and limitations of this study

- The criteria for decision-making quality are listed and defined
- This study utilizes mixed methods to both audit quality of decisions as well as shed light on the utility and feasibility of the tool
- This study is limited to auditing organisational decision-making and does not attempt to audit individual decision-makers
- Only decisions related to a single epidemic within one setting were audited
- The audit process was organisationally led in order to determine the feasibility of the audit.

Introduction

Evaluating decision-making has been recognized as essential to improving health outcomes in a number of contexts[1]. In humanitarian and crisis contexts good decisions at program, sector and response level are especially critical to saving lives and improving response[2]. Despite recent

For peer review only

calls for concerted evaluation of decision-making in these settings[3,4], the literature is still sparse. In crisis-affected settings experiencing epidemics, evaluations to date have primarily focused on establishing the extent to which epidemic response outcomes (for example reduced transmission, improved case management) have been attained. Less attention has been given to evaluate the processes underlying these outcomes (for example how response activities were decided and implemented) [5]. Process evaluations have largely been conducted in high-income countries or after high-profile epidemics to retrospectively determine which decisions led to response failures[6–8].

The need to improve decisions in epidemic settings is especially relevant considering the ongoing COVID-19 pandemic, in which decision-makers contend with a plethora of competing emergencies[9]. Decision support tools have been developed for a variety of settings and purposes but are particularly ubiquitous in corporate business management[10] and in the pharmaceutical and health technology sector[11] for example in the development and licensing of medicinal products, equipment and diagnostics. They are less frequent in humanitarian contexts where they focus primarily on supporting decision-makers in optimizing selected response interventions through for example specifying target populations or modalities of delivery[12,13]. To our knowledge there does not exist an evaluation tool that examines the quality of decision processes within epidemic settings. In a previous study in Somalia, we described factors relevant to COVID-19 decision-making processes; results suggested a need for such a tool.[9]

We thus developed a decision-making audit tool to support epidemic responders in assessing and improving the quality of organisational decision processes. Here, we report on a pilot application of this tool amongst two epidemic response organisations in Somalia, a country grappling with the ongoing COVID-19 pandemic and ongoing humanitarian crises [14].

Aim and Objectives

The overall aim was to evaluate the utility and feasibility of a decision support tool for epidemic responders in humanitarian settings.

For peer review only

The specific objectives were to: (i) document the implementation of the tool among select epidemic responders in Somalia; (ii) generate epidemic decision-making scores using the decision support tool; and (iii) explore the feasibility and utility of the tool through key informant interviews of epidemic responders involved in the audit. We refer to objectives 1 and 2 as comprising the audit while objective 3 is referred to as the evaluation.

Methods

Study Design

This study used a mixed methods programme/response evaluation design. It comprised quantitative and qualitative data collection to both assess the decision-making in epidemic response as well as the feasibility and perceived utility of the tool by intended users (decision-makers). We then revised the tool based on this data. The final structure of the decision-making evaluation tool was determined from input collected during the pilot evaluation.

Patient or public involvement

Neither patients or the public were involved in the design of this study.

Description of the tool

We developed an evaluation tool, protocol and Standard Operating Procedure (SOP) (Supplementary Files 1-4) founded on previous reviews [15] and extensive fieldwork in an epidemic setting[9]. This foundational work resulted in findings of very low coverage of epidemic evaluations globally, limited focus on decision processes, lack of standardized evaluation methods as well as an absence of a comprehensive evaluation framework suitable for epidemic response. As a result, we developed an Adaptive Epidemic Response (AER) evaluation framework[16] and drawing from an assessment of the COVID-19 response in Somalia[9], we derived a decision-making framework. The tools and SOPS are based on this decision-making framework. The tool is comprised of three sections and is summarized below (Figure 1).

For peer review only

The first section (Part A) of the tool is the context analysis. which requires information on the historical, geographic and health context in which the epidemic is occurring.

The second section (Part B) entails identification of critical decisions in the response to the specific epidemic. These critical decisions are identified and selected with reference to five Critical Decision Characteristics that differentiate critical decisions from minor or low impact decisions[2] (Table 1).

Table 1. Characteristics of 'critical' decisions

Characteristic	Definition
Consequential	Decision shapes the response to a significant degree
Not reversible	Decision is difficult to overturn or reverse, at least in the short term
Strategic	Decision involves substantial shift in terms of action taken, resources committed or precedent set
Uncertain	'Decision is made in context of substantial uncertainty with complex array of options
Reputationally risky	Decision entails a high level of organisational reputational risk

In the third section of the tool (Part C), users assess each of the selected decisions against the criteria for 'quality' decision-making. Although there is no agreed definition of a good quality decision[17], we have derived a number of defining criteria from previous published research on health prioritization[18], organizational decision-making[17,19,20] as well as decision-making in emergencies[21]. The 11 criteria are grouped into 4 dimensions: transparency, contestability, accountability, and rigour (Table 2).

Table 2. Criteria for assessing decision quality

Dimension	Criteria	Description
**Transparency**	Inclusivity	The extent to which the process was inclusive, reflected in heterogeneity in rank and roles amongst decision makers involved.
	Use of explicit decision-making criteria	The extent to which the goals and objectives of the decision were clearly pre-specified. The absence of post-decision rationalization.

	Following clear process or method	The extent to which a priority setting process was in place, reflected in demonstrated use of priority setting frameworks, decision trees or other mechanism.
	Use of mechanism to publicise rationale	The extent to which clear documentation on the decision exists as well as the method used to communicate decisions.
**Contestability**	Opportunity for revision	The extent to which there existed scope to revise and overturn a decision including the debating of alternatives and description of how consensus was reached.
	Was the decision devolved?	The degree to which participants in closest proximity to the epidemic (e.g. subnational level) or local technical experts participate in the decision, including consideration of rank.
**Accountability**	Engagement with affected communities	The degree to which affected communities were involved in the response decision-making including at a minimum whether they were informed of the response activities and what effect this notification had on the communities.
**Rigour**	Explicit outcome	The extent to which intended outcomes of the decision were clearly articulated, including through setting of targets.
	Feasible outcome	The extent to which feasibility was considered in decision-making including debating of alternatives.
	Strengthens healthcare system	The extent to which the decision was in-line with wider strategy including the strengthening of the health system
	Evidence based	The extent to which the decision was based on strong public health rationale and robust scientific information.

For peer review only

Users are required to rate on a Likert scale (a type of linear rating scale commonly used to measure respondents' opinions or attitudes), the strength of evidence supporting the fulfilment of each criterion. They are also expected to provide reference to documentary or observational evidence to support their rating. Users' individual scores are then aggregated, and a summary score is generated.

Data Collection

Audit

We invited three organisations actively engaged in the COVID-19 response in Somalia to pilot the audit tool. After separate presentations in which the protocol and study objectives were

explained by the first author, we partnered with two organisations (WHO Somalia and CARE Somalia) in September 2021. Both organisations then nominated audit focal persons tasked with recruiting relevant colleagues (a ‘decision-making committee’), gathering the necessary documentation, and completing part A of the tool. Focal persons were instructed to recruit colleagues who had an active role in decision-making within the response as well as to compile key documents which informed or documented decisions. The location and modality of the audit (combination of face to face and remote sessions) and timeline were jointly determined by the audit focal persons and the first author. Study information and consent forms were shared, and written consent was obtained from all participants in the pilot.

The decision-making audit and feasibility evaluation took place in Garowe, Somalia from November 8-22nd 2021 with CARE Somalia and in Mogadishu from November 22- December 8 with WHO Somalia. The audit was led by each organisation’s focal persons with the facilitation of the first author. Each audit was comprised of 3 group sessions with the decision-making committee interspersed with individual sessions (Figure 2). The first group session introduced the audit approach, tool, timeline and expected outputs. Participants were then asked to review Part A (context analysis) of the tool and incorporate any changes before the next group session. In the second group session, participants were introduced to Part B (selection of critical decisions) and were tasked with individually generating their perceived list of critical decisions. In the third group session, participants were asked to each present and advocate for their selection of critical decisions to the wider group. The group was then tasked with forming a consensus on at least 3 of the decisions to carry forward to the last stage. After consensus was reached, participants were asked to complete Part C of the tool for each decision in which they assessed the availability and strength of evidence supporting the fulfilment of various quality decision-making criteria. Audit focal persons and the LSHTM researcher then aggregated the scores and created a draft scorecard.

For peer review only

Evaluation

All audit participants were then invited to take part in a key informant interview to expound on their selected decisions and explore their views on the evaluation process and tools. After the key informant interviews, the draft scorecard was shared with all participants to provide individual written feedback on the results. The final scorecard was then developed by the audit focal persons and first author and shared with the senior management of the respective organisation. Finally, key informant interviews were conducted by the first author with senior managers who were not involved in the other aspects of the audit and evaluation to solicit their views on the results and their understanding of the process. The tool and related documents were then updated based on pilot findings.

A total of 18 key informant semi-structured interviews were conducted in English or Somali by the first author either face-to-face or via Zoom (Table 3). The first author is a fluent Somali speaker who has substantial experience in health evaluations and epidemic response in the Somali context. The topic guide covered participants' views on the feasibility of the audit process including time and human resources required, the utility of the audit tool in understanding and improving decision making as well as recommendations for future iterations.

Table 3 Data collection by method and type of participant or setting

Primary Data collection	Type of Participants	Number of Participants
**Key informant interviews**	WHO audit team	7
	WHO Staff- Other	2
	WHO Senior management	2
	CARE Somalia audit team	5
	CARE Somalia senior management	2
**Group Sessions**	WHO audit Team	7
	CARE audit Team	6

Interviews were recorded for transcription and analysis purposes. Each interview took approximately 30-45 minutes and participants were given the option of complete or partial anonymity in which they considered whether they would like their name or only their role to be published. A total of 13 participants took part in the group sessions and notes were taken by the

first author. All participants in the CARE audit were national staff members while the majority of WHO participants were international (85%).

Data Analysis

Generation of audit tool scoring

The scorecard for the audit was produced by calculating for each of the 11 criteria assessed, the average of the individual participants scores. Average scores were also presented according to the four dimensions listed in table 2.

Evaluation of the audit implementation

In order to evaluate the implementation of the audit tool as it relates to the feasibility and utility, interview transcripts and group session notes were analysed using a thematic approach[22] using Nvivo software. Emerging themes were grouped into two categories: those relating to identified critical COVID-19 decisions and those relating to the implementation of the audit. Lastly the validity of the tool was evaluated. The interrater agreement of each organisation's scoring was calculated using Fleiss' Kappa.

In the following results section, we focus on the implementation of the audit while details of specific decisions can be found within the decision-making scorecards in the supplemental material (Supplementary File 5, Supplementary File 6).

Results

Implementation of the decision-making audit

Participants in both organisations opted for a mixture of face-to-face and remote (video conference) data collection citing the busy schedule and geographic dispersion of the participants. Although preparatory material including documentation was shared prior to the arrival of the LSHTM focal person, some participants were not immediately aware of the purpose of the audit. The delayed setup of shared folders containing key audit documentation proved difficult for participants' engagement with the audit and subsequent evaluation. Furthermore, the

For peer review only

period in which this audit took place coincided with multiple projects including end-of-year evaluations, annual planning and multiple pre-scheduled activities, hindering the timely recruitment of participants. Many participants were not able to be physically present in the location of the audit due to competing engagements but were nevertheless present through remote means.

Decision Selection

CARE participants had difficulty identifying critical decisions with individual participants submitting on average only 1 critical decision for consideration. However, consensus was reached fairly quickly once the proposed decisions were deliberated by the wider group. In contrast, WHO audit participants put forward 15 decisions for consideration. Consensus took longer to achieve as participants vigorously advocated for their proposed individual decisions. Eventually the group reached consensus on 4 decisions to proceed to the next stage (Table 4). In both groups, moderate reference was made to the Critical Decision Characteristics (Table 1) with participants arguing for decisions in their particular area of work. Participants in WHO were particularly inclined to view decisions in terms of their alignment with the Incident Management Support Team (IMST) Pillars[23]. Additionally, there was an effort by some participants to reach consensus by collapsing together multiple decisions into a single decision in order to capture all opinions. In both WHO and CARE evaluations, participants focused on positive decisions (i.e. a decision to take an action) and may have overlooked negative decisions (i.e. decisions not to take action).

Table 4. List of selected critical decisions by organisation

CARE Somalia	WHO Somalia
Closure of offices and restriction of staff movement	Scale up of case management through improving access to therapeutic oxygen
Modification of Nutrition programme guidelines to be COVID-19 sensitive including changing patient assessment, facility management and outreach procedures	Establishment of 3 key PCR labs in Mogadishu, Garowe and Hargeisa to strengthen diagnostic and surveillance capacity

For peer review only - http://bmjopen.bmj.com/site/about/guidelines.xhtml

Scale up of COVID-19 response activities such as community outreach, contact tracing, provisioning of IPC supplies in Sool and Sanaag Regions	Implementation of the Incident Management Support Team to coordinate COVID-19 response
	Establishment of rapid response teams to scale up surveillance capacity within high priority districts

Decision Scoring

Participants in the CARE audit on average scored their decision-making quality lower than those of WHO. Both organisations scored lowest in accountability to target populations. The scoring of WHO participants on the decision to focus on case management through oxygen scale up demonstrates the low scoring of accountability relative to the other dimensions of quality decision-making (Figure 3). Full details can be found in the supplementary materials (Supplementary File 5, Supplementary File 6).

Participants in CARE scored their decisions lower in contestability compared to WHO. Both organisations rated themselves highly in transparency, particularly in the inclusivity criteria. However, the key stakeholder mentioned under these criteria differed, with CARE highlighting the need to improve inclusive decision-making within the organisation (national staff vs country office senior management) while WHO referred to inclusivity with government authorities. Lastly, CARE participants rated their decision-making as less rigorous than WHO's, citing their unfamiliarity with the evidence underlying the decisions. By contrast, WHO participants cited use of the latest scientific findings and alignment with health systems strengthening frameworks.

Interrater agreement

There was very low interrater agreement amongst participants in both WHO (Kappa = -0.000948, P= 0.977) and CARE (Kappa = 0.0684, P= 0.172) audit groups with a slightly higher interrater agreement amongst CARE participants, indicating high heterogeneity of scores.

For peer review only

Perceptions of the audit tool

Utility

Participants described clearly seeing the purpose and value of the audit. For example, one participant from WHO said, "*We learned that decisions are not just simply taken as decisions, but you have to have a clear decision-making process, a methodology, or criteria, or something, but not necessarily that we just come and say, "Oh, let's do this and that"*. Participants articulated the ongoing need for such an exercise, arguing: "*If there's no understanding of how a decision happened, there's no way to improve on it" (WHO Participant 5).* Critically, they understood that this exercise was meant as an internal audit: *"It was really for us to be able to critically evaluate ourselves as an organisation" (CARE Participant 3).* Some participants also saw the potential utility in a wider range of contexts, beyond epidemics: "*I think [the audit tool] can apply to any decision in humanitarian emergency response" (WHO Participant 2).*

Participants mentioned learning new things about the decision process including recognizing neglected characteristics of good decision-making:

> *"When I was trying to fill in some of the scores, I realized that these are very important [aspects] that were neglected before. For example, devolving certain decisions, it is very important. Not just like you sit up at national level, you decide for anyone and you decide for people at all levels, I think if we devolve certain functions it is good". WHO Participant 1*

They also were able to identify areas for improvement. "*I think for accountability most of the evidence was very minimal. It just got me thinking of ways in which we can involve the communities in decision-making." (WHO Participant 4).*

Participants also highlighted that the audit was useful in allowing them to be more reflective. *"It helped me look at things objectively. If I was not part of the Incident Management Support Team (IMST) would I have advocated for this decision or not?" (WHO Participant 4).* They highlighted the relevance of the decision-making critical characteristics as useful in their work and recognised these as reflective of best-practice: *"I think the tool helped a lot to give me a reflection as to what should be done. I think I learned a lot going through the questions." (WHO Participant 1).*

For peer review only

Participants mentioned the gradual understanding they accrued throughout the audit process as strengthening their engagement with the audit. *"I think some of it was clear, but some of it was also not very clear. I think I understood the methodology with time, of course, when you explained and when we were having the group discussions. That is why in the last forms, I revised and added a few components and sent back to you. I think as usual when people read something, it might not be clear in the first place, but when you explain it, it becomes clear". (WHO Participant 4).*

Participants also noted the benefit of the group format in deriving new insights and creating a useful forum for reflection to review decisions that otherwise didn't exist in their typical organisational practices:

> *"We all had our own ideas, but when we met as a group, there were a few other things that came out that probably I didn't think about as an individual, which were very valid". WHO Participant 4*

> *"I also liked that we were able to meet as a group and look at these things because to be very honest, I don't feel like we have that as a group, as an IMST team to review the decisions that we made or look at what we could have done differently, or whether we felt this decision was important or not. This was a very unique exercise and I'm glad that we got an opportunity to do this." WHO Participant 7*

Participants expressed their commitment to use the lessons learned in future decision making:

> *"I will inform my colleagues, 'Please, let us have certain criteria to use, let us make our methodology clear, and let us document everything,' because at some point down the road, we should know our decision-making process. This has helped a lot and will change a lot in the future." WHO Participant 5*

However, participants mentioned that there was a certain element of subjectivity which was difficult to overcome:

> *"It might be very difficult to understand exactly the reality on the ground, because it depends on the person. Not only the person but also, the involvement of the person in the response activity, and also the understanding of the context. If you are someone who is quite new to Somalia, and maybe who doesn't have detailed*

For peer review only

> *involvement, you might give a different ranking than a person who's spent more time in Somalia and directly engaged in response activity". WHO Participant 2*

Feasibility

In terms of the audits' feasibility, participants expressed that they were easily able to comprehend the process. *"[The tool] was very understandable and also, it was very user friendly." (CARE Participant 1)*. However, a few participants noted some areas for improvement: *"I'm looking at this tool being used for an outbreak. If that is to happen, as I said, we need to really condense some aspects, and lump some of these criteria together to cut on time." (CARE Participant 3).*

In terms of time, participants expressed that the duration of the audit was acceptable but that the time of year chosen for the implementation could have been improved. *"In terms of length of the time, this is fine. This amount of time we need to give. The issue is the timing of the programming, because in some of the months, we are very busy, like last quarter of the year we are very busy." (CARE Participant 5).*

As a result, some participants expressed their confidence that they could implement this audit independently at a more opportune time. However, others expressed their preference for this work to be led by an independent evaluator.

> *As the independent outsider, everybody can open up to you, and this is the best advantage I can see. I also saw something similar in my previous organization, that again, it is the power dynamic or the personality thing that not everybody speaks openly, or the culture doesn't allow it but when somebody from outside comes, everybody opens up. At the end, you come up with some really good findings and recommendations" WHO Participant 1*

In their engagement with the audit tool, participants faced a challenge in coming to a consensus on the limited number of decisions to score. *"Considering the response that we are doing in the country, it might be a bit challenging to come up with one or two out of so many critical decisions." (WHO Participant 2).*

For peer review only

Additionally, there were challenges in accessing documentation relevant to the audit. "*Maybe the big challenge is getting the evidence, documentation, preparing the documentation, extracting the email, finding the document, it might be time-consuming." WHO Participant 3*. Where documents did exist, some participants stated that not all staff may have access. Lastly, some participants highlighted language or conceptual understanding barriers as challenges in the audit. *"At some point, I felt like we were not looking at the decisions, but we were looking at pillars." (WHO Participant 4).*

Selection of decision-making committee

Participants noted that the composition of the decision-making committee had the potential to affect the audit outcome because of a lack of detailed knowledge on how decisions were made. "*If somebody is not involved in a certain pillar, they would struggle to look for evidence or they might not be able to direct somebody to a particular document." (WHO Participant 2)*

They mentioned that all who were part of the audit had some role to play in COVID-19 decision making but conversely not all who had decision-making roles participated in the audit.

Some participants highlighted the potential of overlooking critical decisions when the primary responsible staff member was not present in the audit.

> *"Later I thought about COVID-19 vaccine, and I wondered if we could have included the focal person and whether they would have felt that the introduction of the COVID-19 vaccine into the country was a critical decision. I thought about it, and I thought maybe nobody mentioned it, or nobody talked much about it during the discussion because the focal point was not there. WHO Participant 4*

Participant Recommendations

Participants made several recommendations to improve the utility and feasibility of the audit for future iterations. These included broadening the scope of participants by more clearly outlining who should participate.

Participants also suggested incorporating the emergency response cycle into the evaluation tool. *"There are emergency response cycle phases such as preparedness, implementation,*

For peer review only

monitoring, and evaluation […] There are critical decisions at each phase which need to be evaluated." (WHO Participant 2)

They also made several suggestions to improve the implementation including ensuring more face-to-face rather than virtual interactions amongst participants and undertaking a joint review of documentation to improve participant familiarity with the available documents.

Views of senior management

Senior management largely agreed with the findings of their respective scorecards. They did, however, provide further detail and rationale for some aspects of the selected decisions. For example, senior managers in both organisations cited mitigating factors in explaining lower-than-expected scoring in some dimensions. Referring to closure of offices and restriction of staff movements, one CARE senior manager explained:

> *"When the first case of COVID-19 was reported in Somalia in March, I think it was around 16, 17. There was a bit of panic in that it was not business as usual. When it comes to between life and death, certain positions have to be taken. As a result of that, yes, the senior management team made a decision to ensure that because we are accountable to everyone and every staff, and every staff exposed to any risk as a result of negligence of the organization, then the organization takes responsibility." CARE Senior Manager 1*

A WHO senior manager explained the circumstances behind a decision to increase testing capacity:

> *"Obviously there's scope for improvement, but sometimes when we are in a pressured situations and we see that the PCR based labs are overwhelmed and at the peripheral level there is no access or testing facilities are not there, and the GeneXpert machines are also not functioning, then sometimes we make decisions and implement swiftly, and then [later] we provide other evidence and information of the value and effectiveness of those strategies". WHO Senior Manager 1*

They did however agree on gaps highlighted through the scorecard stating: "*I would say that [decision-making] can become more inclusive. I think that we are just talking internally amongst ourselves and we are not engaging sufficiently with our government counterparts. I think that that's a fundamental mistake that we are doing, and we continuously do so, because of maybe convenience, because of maybe comfort."* *(WHO Senior Manager 2).*

For peer review only

Discussion

This pilot evaluation of a tool to audit decisions taken during epidemic response demonstrates that the tool can be successfully deployed even in the midst of an ongoing response and even in circumstances where responders are dealing with unusually high workload. The audit also elicited very positive user feedback with participants expressing a willingness to implement it in future epidemic responses. Importantly, participants viewed the audit as reflecting good decision-making practice indicating high levels of acceptability.

As populations affected by epidemics continue to grow[24], there is an urgent need to improve epidemic response particularly through improved decision-making. Much of the focus has been on improving decision outcomes and less attention has been paid to decision-making processes[25]. However, strengthening decision processes can improve decision-making quality[26] and, accordingly, outcomes[27] including more efficient allocation of resources, improved accountability and greater coordination.

The literature on decision-making in emergencies has been underpinned by an analytically oriented[28] conceptualization of decision making in which the 'correct' decision is sought[29,30]. This is in contrast to the process conceptualization in which the decision is pursued correctly. Towards this end, decision support approaches have been developed to optimize the decision-making process to achieve efficient[2] and timely decisions[31] but have largely not considered the aspect of quality. The evaluation of this tool within the COVID-19 response in Somalia contributes towards filling this gap.

The audit sheds light on shortcomings in the quality of decision processes within piloting organisations and provided participants opportunity for reflection and key areas for improvement. The tool was found to be highly adaptable as it allowed organizations to evaluate decisions that were considered significant by their staff. Nevertheless, while the audit instructed organisation to select participants that were largely reflective of staff making or implementing response decisions, some participants expressed reservations about the final composition of the evaluation panel. The scoring of some decision dimensions was however largely reflective of the panel's

For peer review only

experiences and characteristics. For example, amongst CARE participants, the lower scoring on the contestability of some decisions might be due to their roles as national staff who may not have had sufficient opportunity to contest decisions. The rollout of the tool allowed for gradual understanding to develop and for participants to question their basic assumptions. For example, participants were able to acknowledge elements that were absent from their decision-making process. They were also surprised by the paucity of documentary evidence within their organisations and were able to contrast this with their initial self-assessed high scoring. The lower-than-expected agreement amongst raters could possibly due to lack of consensus on what various levels of evidence represented. While limited documentation does not necessarily equate with poor decision processes, it does make subsequent evaluations more challenging.

Furthermore, the audit tool was found to be highly flexible as users highlighted its potential utility in assessing the quality of decision-making in broader humanitarian response by generating a quantitative measure of decision quality that can allow for tracking over time. In addition to the retrospective assessment of decisions, the tool can also be used in real time to improve decision-processes.

Limitations

Although this pilot was undertaken within two organisations, it assessed decision-making related to a single epidemic within one country. While this pilot study was led by the first author who was external to both organisations, future iterations led entirely by internal staff members may be more vulnerable to censorship if results reveal low decision quality. However this is a challenge that is present in global health arena in general[32] and a number of resources have been published to strengthen independence of evaluations[33–35]. Additionally, much of the scoring relied on the subjective assessment of the individual rater and may thus have been reflected in the lower-than-expected interrater correlation. Furthermore, this pilot focused on face validity (the extent to which a tool appears to measure a concept) rather than on content validity which requires further exploration and methods. Additionally, we did not use quantitative methods such as factor analysis to reduce the number of items within the critical decision criteria as there were too few items. Instead, we relied exclusively on direct feedback from the participant interviews.

For peer review only

Definitions for the decision- making criteria were not exhaustive and could have been further expanded. For example with regards to community engagement criteria we provided a minimum definition rather than broader definition[36]. Finally, the methods described in this study assessed only organisational decision-making rather than individual and as such cannot be used to assess the decision-making of individual epidemic responders.

Recommendations

The audit can be further piloted in a wider range of crisis settings and amongst different response actors to ascertain its feasibility and utility in diverse settings. Additionally, the audit should be conducted internally by response actors in order to compare how the audit is implemented when it is entirely independent of an external facilitator. Furthermore, the audit should be conducted periodically in order to determine whether there has been a quantitative change in the decision quality scoring. Lastly, the audit tool could also be integrated within the WHO recommended intra-action reviews[37] as well as after action reviews[38] for health emergencies.

Conclusion

Strengthening decision-making processes is key to realizing the objectives of epidemic response. This pilot evaluation contributes towards this goal by the testing what, to our knowledge, may be the first tool designed specifically to assess quality of decision-making processes in epidemic response. The tool has proven feasible and acceptable in assessing decision-making quality in an ongoing response and has potential applicability in assessing decision-making in broader humanitarian response.

For peer review only

Declarations

Contributorship statement

This study was designed by AW with input from FC. The data collection was undertaken by AW, AO, FH and AA. The analysis was undertaken by AW with input from KB, FC, and JP. The first draft was written by AW. All authors have contributed significantly to the final draft.

Competing interests

We declare no competing interests.

Funding

This work was supported by UK Research and Innovation as part of the Global Challenges Research Fund, grant number ES/P010873/

Data sharing statement

All data relevant to the study are included in the article or uploaded as supplementary information

Ethics approval and consent to participate

Ethical approval for this study was obtained from the National Institute of Health Research of the Federal Republic of Somalia (Ref: NIHS0102208) and the ethics review committee of the London School of Hygiene & Tropical Medicine (Ref: 26369). Informed written consent was taken from all key informants.

Acknowledgements

We would like to acknowledge Md Mamunur Malik WHO country representative and Iman Abdullahi CARE country representative for agreeing to pilot this tool in their respective organisations. We would like to thank participants for sharing their insights.

Reference

1 Glarum J, Adrianopoli C. *Decision Making in emergency management*. 1st ed. Oxford: : Elsevier 2020.

2 Cosgrave J. Decision making in emergencies. *Disaster Prev Manag An Int J* 1996;**5**:28–35. doi:10.1108/09653569610127424

3 Campbell L, Knox P, Alnappaper C. *Making operational decisions in humanitarian response: A literature review*. 2018. https://www.alnap.org/our-topics/leadership. (accessed 3 Jun 2020).

4 Knox Clarke P, Campbell L. Decision-making at the sharp end: a survey of literature related to decision-making in humanitarian contexts. *J Int Humanit Action* 2020;**5**:1–14. doi:10.1186/s41018-020-00068-2

5 Warsame A, Murray J, Gimma A, *et al.* The practice of evaluating epidemic response in humanitarian and low-income settings: a systematic review. *BMC Med* 2020;**18**. doi:10.1186/s12916-020-01767-8

6 Horton R. Offline: The mistakes we made over Ebola. Lancet. 2019;**394**:1494. doi:10.1016/S0140-6736(19)32634-0

7 S. M, D. S, M.A. P, *et al.* Will Ebola change the game? Ten essential reforms before the next pandemic. the report of the Harvard-LSHTM Independent Panel on the Global Response to

For peer review only

Ebola. *Lancet* 2015;**386**:2204–21. doi:http://dx.doi.org/10.1016/S0140-6736%2815%2900946-0

8 García-Basteiro A, Alvarez-Dardet C, Arenas A, *et al.* The need for an independent evaluation of the COVID-19 response in Spain. Lancet. 2020;**396**:529–30. doi:10.1016/S0140-6736(20)31713-X

9 Warsame A, Fuje M, Checchi F, *et al.* Evaluating COVID-19 decision-making in a humanitarian setting: the case study of Somalia. *Plos Glob Public Heal* 2022.

10 Stenfors S, Tanner L, Syrjänen M, *et al.* Executive views concerning decision support tools. *Eur J Oper Res* 2007;**181**:929–38. doi:10.1016/J.EJOR.2006.06.024

11 Bujar M, McAuslane N, Walker SR, *et al.* Evaluating quality of decision-making processes in medicines' development, regulatory review, and health technology assessment: A systematic review of the literature. *Front Pharmacol* 2017;**8**:189. doi:10.3389/FPHAR.2017.00189/BIBTEX

12 Immunization, Vaccines and Biologicals Vaccination in acute humanitarian emergencies: a framework for decision making. Published Online First: 2013.http://www.who.int/immunization/sage/sage_wg_hum_emergencies_jun11/en/ (accessed 13 Feb 2022).

13 Vargas Florez J, Lauras M, Okongwu U, *et al.* A decision support system for robust humanitarian facility location. doi:10.1016/j.engappai.2015.06.020ï

14 UNOCHA. Humanitarian Response Plan Somalia Humanitarian Programme Cycle 2021 Issued February 2021. 2021. www.unocha.org/somalia (accessed 22 Apr 2021).

15 Warsame A, Blanchet K, Checchi F. Towards systematic evaluation of epidemic responses during humanitarian crises: A scoping review of existing public health evaluation frameworks. *BMJ Glob Heal* 2020;**5**. doi:10.1136/bmjgh-2019-002109

16 Warsame A, Blanchet K, Checchi F. Towards systematic evaluation of epidemic responses during humanitarian crises: A scoping review of existing public health evaluation frameworks. *BMJ Glob Heal* 2020;**5**:e002109. doi:10.1136/bmjgh-2019-002109

17 Wood NL, Highhouse S, Brooks M, *et al.* INDIVIDUAL DIFFERENCES IN DECISION-MAKING STYLES AS PREDICTORS OF GOOD DECISION MAKING. 2012.

18 Kapiriri L, LaRose LB. Priority setting for disease outbreaks in Uganda: A case study evaluating the process. *Glob Public Health* 2019;**14**:241–53. doi:10.1080/17441692.2018.1498532

19 Peniwati K. Criteria for evaluating group decision-making methods. *Math Comput Model* 2007;**46**:935–47. doi:10.1016/J.MCM.2007.03.005

20 Bang D, Frith CD. Making better decisions in groups. R. Soc. Open Sci. 2017;**4**. doi:10.1098/rsos.170193

21 Knox Clarke P. How can we improve decision-making in humanitarian coordination? 2016. www.alnap.org/coord-meeting. (accessed 27 Aug 2020).

22 Bryman A. *Social Research Methods*. Oxford: 2012.

23 World Health Organization (WHO). Emergency Response Framework Second Edition. Taiwan Rev. 2017;**69**:76.

24 OCHA. Global Humanitarian Overview 2021 [EN/AR/FR/ES] - World | ReliefWeb.

For peer review only

https://reliefweb.int/report/world/global-humanitarian-overview-2021-enarfres (accessed 18 Mar 2021).

25 Warsame Id A, Fuje Id M, Id FC, *et al.* Evaluating COVID-19 decision-making in a humanitarian setting: The case study of Somalia. *PLOS Glob Public Heal* 2022;**2**:e0000192. doi:10.1371/JOURNAL.PGPH.0000192

26 George B, Desmidt S. Strategic-Decision Quality in Public Organizations: An Information Processing Perspective: *https://doi.org/101177/0095399716647153* 2016;**50**:131–56. doi:10.1177/0095399716647153

27 Schippers MC, Rus DC. Optimizing Decision-Making Processes in Times of COVID-19: Using Reflexivity to Counteract Information-Processing Failures. *Front Psychol* 2021;**12**:2261. doi:10.3389/FPSYG.2021.650525/BIBTEX

28 Sanderson C. Analytical models for decision-making. *Open Univ Press* 2006.

29 Berger L, Berger N, Bosetti V, *et al.* Rational policymaking during a pandemic. *Proc Natl Acad Sci U S A* 2021;**118**. doi:10.1073/PNAS.2012704118/-/DCSUPPLEMENTAL

30 Morgan O. How decision makers can use quantitative approaches to guide outbreak responses. Philos. Trans. R. Soc. B Biol. Sci. 2019;**374**. doi:10.1098/rstb.2018.0365

31 Gouglas D, Marsh K. Prioritizing investments in new vaccines against epidemic infectious diseases: A multi-criteria decision analysis. *J Multi-Criteria Decis Anal* 2019;**26**:153–63. doi:10.1002/MCDA.1683

32 Storeng KT, Abimbola S, Balabanova D, *et al.* Action to protect the independence and integrity of global health research. *BMJ Glob Heal* 2019;**4**:e001746. doi:10.1136/BMJGH-2019-001746

33 Sager F, Plegger L. Evaluation and Independence Existing evaluation policies and new approaches. 2016.

34 OECD. PRINCIPLES FOR EVALUATION OF DEVELOPMENT ASSISTANCE. Paris: 1991.

35 Evaluation W office for. Decentralized evaluation for evidence-based decision making WFP Office of Evaluation Decentralized Evaluation Quality Assurance System (DEQAS) Technical Note Independence and Impartiality.

36 Palmer J. How is community engagement normally done? | COVID-19 Hygiene Hub Resources. 2022.https://resources.hygienehub.info/en/articles/4028678-how-is-community-engagement-normally-done (accessed 14 May 2022).

37 Mayigane LN, de Vázquez CC, Vente C, *et al.* The necessity for intra-action reviews during the COVID-19 pandemic. *Lancet Glob Heal* 2020;**8**:e1451. doi:10.1016/S2214-109X(20)30414-9

38 WHO. The global practice of after action review. Geneva: 2019. https://www.who.int/publications/i/item/WHO-WHE-CPI-2019.9 (accessed 16 Feb 2022).

Figure Legend

Figure 1 Structure of the audit tool

Figure 2 Data collection timeline of evaluation

For peer review only

Figure 3 Extract of decision scorecards where accountability was rated low

For peer review only

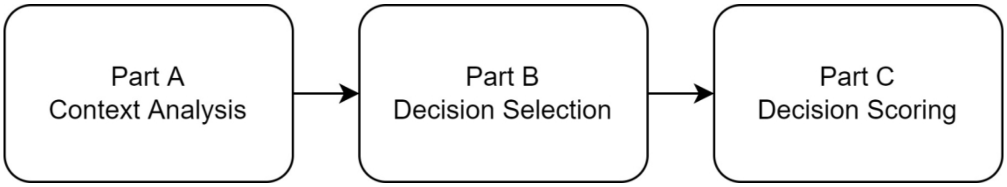

Figure 1 Structure of the audit tool

636x114mm (72 x 72 DPI)

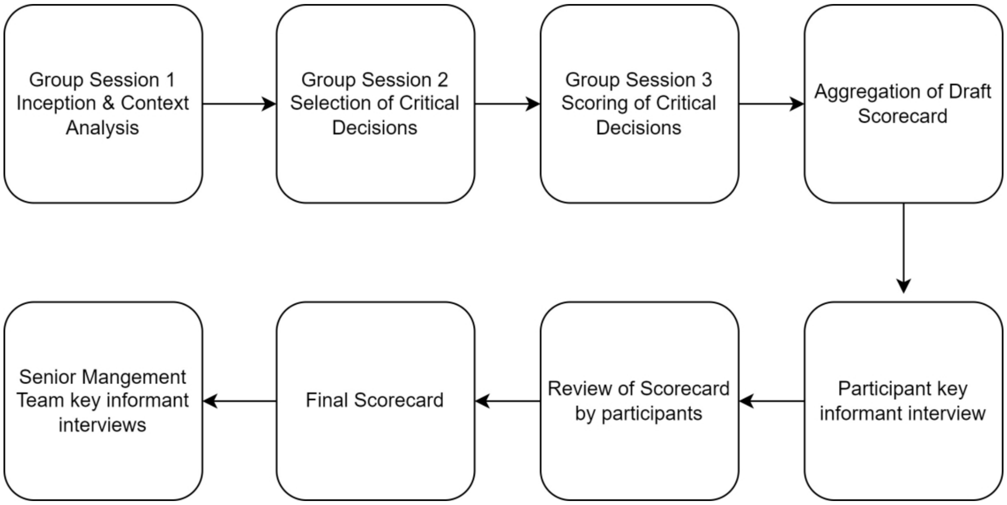

Figure 2 Data collection timeline of evaluation

855x424mm (72 x 72 DPI)

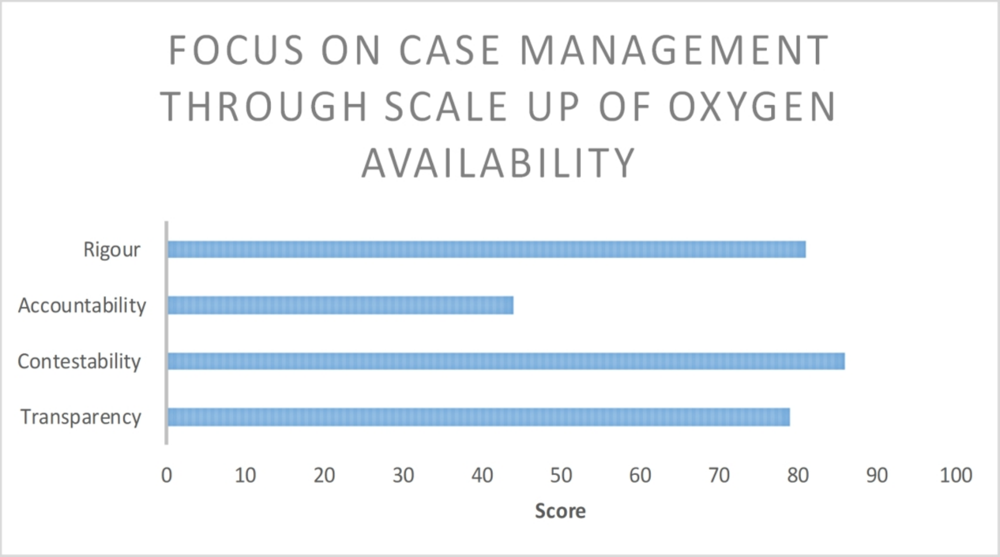

Figure 3 Extract of decision scorecards where accountability was rated low

871x484mm (59 x 59 DPI)

Evaluating the quality of epidemic decision-making in epidemics & humanitarian crises

Standard Operating Procedures (SOP)

For peer review only

Version 1.0
June 30th, 2021

Abdihamid Warsame
Infectious Disease Epidemiology
London School of Hygiene & Tropical Medicine
Abdihamid.warsame@lshtm.ac.uk

Contents

Evaluating epidemic decision-making in humanitarian crises 1
Standard Operating Procedures (SOP) 1
Abbreviations 4
Introduction 5
Rationale 6
Definitions 6
Target audience 6
Evaluation team composition 6
Timeline 7
Section 1- Context analysis 8
1.1 Purpose 8
1.2 Roles and responsibilities 8
1.3 Procedures 8
1.4 Outputs 8
1.5 Associated Documents 8
Section 2- Decision Selection 8
2.1 Purpose 9
2.2 Roles and Responsibilities 9
2.3 Procedures 9
2.4 Outputs 9
2.5 Associated Documents 9
Section 3- Decision Scoring 10
3.1 Purpose 10
3.2 Roles and responsibilities 10
3.3 Procedures 10
3.4 Outputs 10
3.5 Associated Documents 10
Section 4- Dissemination/ Review 11
4.1 Purpose 11
4.2 Roles and responsibilities 11
4.3 Procedures 11
4.4 Outputs 11
4.5 Associated Documents 11
Reference 12

For peer review only - http://bmjopen.bmj.com/site/about/guidelines.xhtml

Annex 13
Document Checklist 13
Decision-making evaluation tool .... **Error! Bookmark not defined.**
Background .... **Error! Bookmark not defined.**
Part A- Context analysis .... **Error! Bookmark not defined.**
Part B- Selection of critical decision points .... **Error! Bookmark not defined.**
Key informant interview guide .... **Error! Bookmark not defined.**
Flow chart .... **Error! Bookmark not defined.**

For peer review only

For peer review only - http://bmjopen.bmj.com/site/about/guidelines.xhtml

Abbreviations

CO- Country Office

COVID-19- Coronavirus disease 2019

RO- Regional Office

HQ- headquarters

Sitrep- Situation report

PHSA- Public Health Situation Analysis

RRA- Rapid risk assessment

SMT- Senior Management Team

KII- Key Informant Interview

FGD- Focus Group Discussion

For peer review only

For peer review only - http://bmjopen.bmj.com/site/about/guidelines.xhtml

Introduction

While epidemics of infectious disease continue to pose a considerable threat to populations in low-income and humanitarian settings, the evaluation of these epidemic responses are limited and heterogeneous [1]. Populations in need of humanitarian assistance or living within low income settings continue to grow[2] and the ongoing COVID-19 pandemic has further exacerbated the impact of these crises[3]. Decision-making in the context of an ongoing epidemic is particularly challenging[13,14]. Previous research has identified a limited number of decision-making factors contributing to poor response [15–17]. However, these findings need further study in order to confirm their relevance to a wider array of contexts experiencing ongoing epidemics

Such settings not only must deal with the epidemic directly and its indirect impacts but must also contend with a host of urgent competing emergencies. As such decision-makers are under heavier strain than those in better resourced and stable environments and require adequate support. COVID-19 is unlikely to be the last epidemic faced by decision-makers in such settings and therefore evaluations such as this are especially critical to strengthening future preparedness and improving response.

In decision-making research, there are two common approaches: process and evaluation. Process oriented approach describes how decisions are actually made within a specific context whereas analytical oriented is more prescriptive and frames decision-making as a problem-solving approach intended to lead to the best outcome. In short the former looks at whether a decision is made correctly while the latter considers whether the correct decision is made. This evaluation is predicated on the former approach.

For peer review only - http://bmjopen.bmj.com/site/about/guidelines.xhtml

Rationale

- This SOP is intended to evaluate the process of epidemic decision-making rather than the impact of any specific decision. Improved decision-making practises can improve outcomes, timeliness and accountability in epidemic response in humanitarian settings.

- In order to contribute to better humanitarian performance, the UKRI awarded a consortium led by the London School of Hygiene & Tropical Medicine to undertake research and produce evidence to support humanitarian responders. This SOP includes stepwise actions required to be undertaken by evaluators as well as the necessary timeframes, roles and responsibilities and resources required.

Definitions

Decision making: choosing between number of options/course of action or a process of problem solving in a specific context (Campbell 2018)

Evaluation- assessment, as systematic and impartial as possible of an activity, project, programme, strategy, policy, theme, sector, operational area or institutional performance.

Target audience

- This standard operating procedure is intended for the evaluation lead within the humanitarian organization. However, it can also be utilized by third party monitors as part of an overarching review of a humanitarian organizations epidemic response.

Evaluation team composition

Although evaluation should ideally be done by independent third party, staff members in the evaluated organization have a significant role to play in the planning, design, implementation and use of this evaluation.

For peer review only

For peer review only - http://bmjopen.bmj.com/site/about/guidelines.xhtml

Timeline

- The evaluation should ideally take place after the first X weeks since the onset of the epidemic. It may also take place immediately after the conclusion of the epidemic

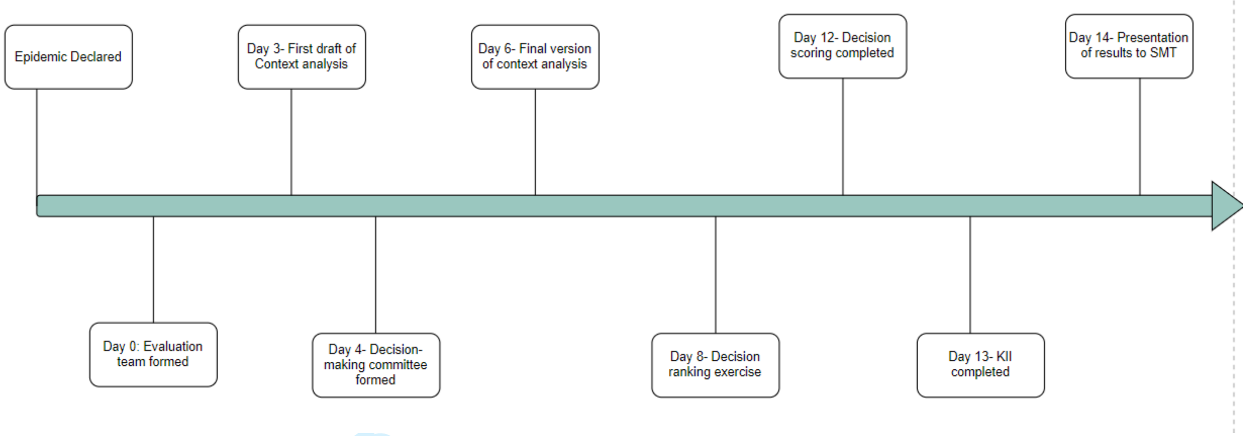

For peer review only

For peer review only - http://bmjopen.bmj.com/site/about/guidelines.xhtml

Section 1- Context analysis

1.1 Purpose

- The purpose of the context analysis is to provide a common base from which to understand the epidemic setting and the actions that have been taken.

1.2 Roles and responsibilities

- The evaluation team lead will lead the exercise and convene the evaluation team to undertake the context analysis.
- The evaluation team will identify principle decision-makers to form decision-making committee

1.3 Procedures

- The evaluation team will undertake a secondary data review in order to produce the first draft of the context analysis
- The review should gather all relevant documentation including:
  - Previous Public Health Situation Analyses (PHSA)
  - Rapid risk assessments (RRA)
  - Response planning documentation
  - Organizational organograms
  - Sitreps
  - Health bulletins
  - Communication products
  - Meeting minutes
- The evaluation team will also identify key internal decision-makers involved in the particular epidemic response who have had significant input in the planning, implementation or evaluation of the epidemic response. These maybe situated within the country office or regional or HQ levels. These may include technical experts, organizational leaders and others
- These decision-makers will be invited to form a committee to contribute to the remaining sections of this evaluation
- The evaluation team lead will circulate the context analysis draft and solicit feedback in a timely manner from the decision-makers
- The evaluation team will consolidate context analysis feedback into a final version

1.4 Outputs

- Consolidated and agreed upon context analysis which summarizes epidemiological description of the epidemic, health resources and services available to the population, health system performance, organizational resources expended in the epidemic response to date, as well as challenges faced in the response

1.5 Associated Documents

See Annex for document checklist, participant list and section 1 of the tool

For peer review only - http://bmjopen.bmj.com/site/about/guidelines.xhtml

Section 2- Decision Selection

2.1 Purpose

The purpose of this section is to produce a unified list of key decisions to evaluate as prelude to evaluate decision-making in the epidemic response.

2.2 Roles and Responsibilities

- The evaluation team lead is responsible for sharing key documentation and producing an initial listing of key decisions
- The decision-making committee is responsible for reviewing, contributing and evaluating the criticality of decisions

2.3 Procedures

- The evaluation team lead will circulate the context analysis and introduce the characteristics for selecting key decisions to the evaluation team.
- In conjunction with the evaluation team, he/she will produce up to 5 key decisions for initial consideration by key decision-making committee identified through the context analysis
- The key decision-makers will review the proposed decisions
- Decision-makers may contribute further decisions for consideration
- Decision-makers will score each of the proposed decisions based on the scoring criteria
- Evaluation team will summarize (take the mean) the score for each decision and select the top 5 decisions using the following formula

2.4 Outputs

- Unified list of no more than 5 key decisions to be further assessed in section 3.

2.5 Associated Documents

See annex for section 2 of the tool

For peer review only

For peer review only - http://bmjopen.bmj.com/site/about/guidelines.xhtml

Section 3- Decision Scoring

3.1 Purpose

The purpose of this section is to ensure that selected critical decisions are evaluated consistently across the assessment criteria and to produce a summary score for the overall epidemic decision-making.

3.2 Roles and responsibilities

- The evaluation team lead is responsible for explaining the evaluation dimensions and criteria
- The evaluation team is responsible for assessing each decision on the criteria

3.3 Procedures

- For each of the selected decisions (maximum 5) the evaluation team will assess whether the necessary decision-making criteria has been met
- They will compile evidence through review of documentation and score the decision on a scale of 1 (no evidence/not met) to 5 (criteria fully met)
- Where there is little or no evidence of a criteria being met, the evaluation team may revert to decision-making committee to provide evidence
- The decision-making committee members are responsible for providing evidence for any specific criteria as requested by the evaluation team
- The evaluation team lead will review the final score for each decision as well compute an overall score for the exercise

3.4 Outputs

- An agreed upon set of scores for each of the critical decisions as well as an overall score

3.5 Associated Documents

Refer to annex for section3 of the tool

For peer review only

For peer review only - http://bmjopen.bmj.com/site/about/guidelines.xhtml

Section 4- Dissemination/ Review

4.1 Purpose

- The purpose of this section is to review and disseminate the findings of the evaluation in order to improve response processes

4.2 Roles and responsibilities

- The evaluation team lead is responsible for compiling the views of organizational members on the overall evaluation process
- The evaluation team lead is responsible for presenting the findings of the evaluation back to the organization
- The organizational leadership is responsible for working with relevant staff members in implementing improvements

4.3 Procedures

- The evaluation team lead should convene staff who have participated in the evaluation and implement focus group discussion (FGD) or key informant interviews(KII) to elicit feedback on the process
- The evaluation team should present the results of both the exercise and participant views to organizational leadership/ Senior Management Team (SMT)in a brief
- The SMT should implement recommendations derived from the exercise

4.4 Outputs

- Identification of decision rated as strong
- Recommendations of decision making dimensions that require strengthening
- Feedback on the utility and feasibility of evaluation
- Identification of areas of the evaluation process that require improvement

4.5 Associated Documents

- See annex for focus group guide and key informant interview guide

For peer review only

For peer review only - http://bmjopen.bmj.com/site/about/guidelines.xhtml

Reference

https://jhumanitarianaction.springeropen.com/articles/10.1186/s41018-020-00068-2#Fn3
https://www.who.int/hac/techguidance/tools/standard_operating_procedures_african_region_en_2014.pdf

https://healthcluster.who.int/docs/librariesprovider16/meeting-reports/updated-phsa-long-form-template-english-june-2019.docx?sfvrsn=81cd6a71_3

For peer review only

For peer review only - http://bmjopen.bmj.com/site/about/guidelines.xhtml

Annex

Document Checklist

No.	Document Type	Details (e.g.)
1	Organogram	*CO organogram March 2021*
2	Meeting Minutes	*Weekly minutes (may 2021-June 2021)*
3		
4		
5		

Evaluation Participants

Evaluation Team	Name (e.g.)	Role
1	*John Doe*	*Team lead*
2	*Pers Indi*	*M&E officer*
3		
Decision-making committee	Name	Role
1	*Fulan*	*Incident manager*
2	*Jama Yulo*	*Operations officer*
3	*Qofa Kale*	*Technical officer*

For peer review only - http://bmjopen.bmj.com/site/about/guidelines.xhtml

LONDON SCHOOL of HYGIENE & TROPICAL MEDICINE

Decision Making Evaluation Instrument

Organization:

Date:

Outbreak:

Part A- Summary of Response

(i) Describe briefly the historical, geographic and health context in which the outbreak is occurring *(maximum 250 words).*

(ii) Briefly describe the course of the epidemic from detection up the present focusing on epidemiology, risk factors and effected populations *(maximum 250 words).*

(iii) Describe what actions your organization has taken in response to the epidemic *(maximum 300 words).*

For peer review only

Part B- Selection of critical decision points

Guide

Considering the following guiding Characteristics, propose 3 -5 critical decision in the response to date. Justify your selection with specific with reference to the characteristics as well as specific examples. A decision does not need to meet all of the following characteristics to be considered critical. These can include decision on beginning, ending or scaling a response, deciding on response options, deciding on target population, as well as deciding on modality of response.

Characteristics of critical decision

I. Consequential- critical decision is consequential and shapes the response to a significant degree
II. Reversibility- a critical decision can be difficult to overturn or reverse at least in the short term)
III. Strategic- entails shift in terms of action taken, resources committed or precedent set
IV. Uncertainty –Wide range of uncertainty/ complex array of options
V. Reputionally Risky- Entails a high level of organisational reputational risk

Decision 1

Description
Describe the decision

Rationale
Justify your proposed decision by selecting the relevant Characteristics
☐ Characteristic 1

☐ Characteristic 2

☐ Characteristic 3

☐ Characteristic 4

☐ Characteristic 5

Narrative
Describe any other reason for considering this decision critical (maximum 200 words)

Decision 2

For peer review only - http://bmjopen.bmj.com/site/about/guidelines.xhtml

LONDON SCHOOL of HYGIENE &TROPICAL MEDICINE

Description

Describe the decision

Rationale

Justify your proposed decision by selecting the relevant Characteristics

☐ Characteristic 1

☐ Characteristic 2

☐ Characteristic 3

☐ Characteristic 4

☐ Characteristic 5

Narrative

Describe any other reason for considering this decision critical (maximum 200 words)

Decision 3

Description

Describe the decision

Rationale

Justify your proposed decision by selecting the relevant Characteristics

☐ Characteristic 1

☐ Characteristic 2

☐ Characteristic 3

☐ Characteristic 4

☐ Characteristic 5

Narrative

Describe any other reason for considering this decision critical (maximum 200 words)

For peer review only - http://bmjopen.bmj.com/site/about/guidelines.xhtml

LONDON SCHOOL of HYGIENE &TROPICAL MEDICINE

For peer review only

For peer review only - http://bmjopen.bmj.com/site/about/guidelines.xhtml

Part C-Decision-making criteria

For each selected decision in Part B, complete the following scoring exercise, keeping in mind to provide the necessary evidence.

Decision: __*e.g. Decision to conduct mobile vaccinations in region Y in May 2020*

Transparency

To what degree were the following criteria met?

Criteria	Fulfilment of criteria<br>*To what extent was the criteria fulfilled in this particular decision*	Evidence of fulfilment<br>*Provide reference to specific sources to support your assessment*
Inclusivity[1]	5- Very Strong evidence	*e.g. Refer to meeting minutes March 24, 2020 as well as response plan April 2020. These show presence of diverse stakeholders in this decision from all departments as well as MoH*
Usage of explicit decision-making criteria[2]	Choose an item.	
Following clear process or method[3]	Choose an item.	
Usage of mechanism to publicise rationale [4]	Choose an item.	

Guide

1. Consider who was involved. Was there heterogeneity in ranks and roles?

2. Were the goals and objectives clearly pre-specified or were decisions rationalized after the fact?

For peer review only - http://bmjopen.bmj.com/site/about/guidelines.xhtml

3. Were there documented priority setting process and/or use of priority setting framework? Was there a decision-tree

4. Was there clear documentation on decisions reached?

For each selected decision in Part B, complete the following scoring exercise, keeping in mind to provide the necessary evidence.

Decision: ____

Contestability

To what degree was the following criteria met?

Criteria	Fulfilment of criteria<br>*To what extent was the criteria fulfilled in this particular decision*	Evidence of fulfilment<br>*Provide reference to specific sources to support your assessment*
Opportunity for revision[5]	Choose an item.	
Was the decision devolved?[6]	Choose an item.	

Accountability

To what degree was the following criteria met?

Criteria	Fulfilment of criteria<br>*To what extent was the criteria fulfilled in this particular decision*	Evidence of fulfilment<br>*Provide reference to specific sources to support your assessment*
Engagement with effected communities[7]	Choose an item.	

For peer review only - http://bmjopen.bmj.com/site/about/guidelines.xhtml

LONDON SCHOOL of HYGIENE &TROPICAL MEDICINE

Rigour

Criteria	Fulfilment of criteria<br>*To what extent was the criteria fulfilled in this particular decision*	Evidence of fulfilment<br>*Provide reference to specific sources to support your assessment*
Explicit outcome[8]	Choose an item.	
Feasible outcome[9]	Choose an item.	
Strengthen healthcare system[10]	Choose an item.	
Evidence based [11]	Choose an item.	

Guide

5. Was there any scope to revise and overturn a decision? How open was the discussion? Were alternatives debated? What were they? Was consensus achieved and if so, how was this reached?

6. What role did respondents in closest proximity to the epidemic (e.g. subnational level) or local technical experts play? What role did organizational leadership play? Who was the most junior decision-maker? Who was the most senior?

7. To what degree were the effected communities involved in the response? Who was involved from the effected community? Were they informed of the response activities? Did this notice make any difference for those notified?

8. Were intended outcomes of the decision clearly articulated? Were targets set? What were they?

9. Were alternatives debated? What were they?

10. Was there evidence that this decision was in-line with wider strategy? What pillar of the health system were targeted?

For peer review only - http://bmjopen.bmj.com/site/about/guidelines.xhtml

11. Was “Strong public health rationale and solid scientific information provided to justify health measures?” What evidence was utilized? What evidence was missing?

Part D- Summary Decision-making Score

Scoring formula

For each assessed decision, utilize the formulae below to populate the table

Transparency score = average of criteria 1-4

Contestability= average of criteria 5-6

Accountability= score of criteria 7

Rigour= average of criteria 8-11

Dimension	Average	Score
Transparency		
Contestability		
Rigour		
Accountability		

For peer review only

For peer review only - http://bmjopen.bmj.com/site/about/guidelines.xhtml

LONDON SCHOOL of HYGIENE &TROPICAL MEDICINE

Recommendations

Based on the results above, briefly describe what specific steps you recommend in order to improve the decision-making process in this epidemic *(maximum 300 words).*

For peer review only - http://bmjopen.bmj.com/site/about/guidelines.xhtml

LONDON SCHOOL of HYGIENE &TROPICAL MEDICINE

For peer review only

For peer review only - http://bmjopen.bmj.com/site/about/guidelines.xhtml

For peer review only

For peer review only - http://bmjopen.bmj.com/site/about/guidelines.xhtml

Decision-making Scorecard
WHO Somalia's COVID-19 response

Evaluation study

Nov 22nd- December 8th 2021

Abdihamid Warsame
Faculty of Epidemiology and Population health
London School of Hygiene and Tropical Medicine

For peer review only

Critical Decisions

Participants were advised to select important decisions for critical evaluation based on the following criteria on what constitutes a critical decision.

Characteristics

A critical decision is one which has one or more of the following characteristics:

I. Consequentiality-: A critical decision is consequential and shapes the response to a significant degree
II. Reversibility-: A critical decision can be difficult to overturn or reverse at least in the short term
III. Strategic: A critical decision entails a significant shift in terms of action taken, resources committed or precedent set
IV. Uncertainty: A critical decision entails a wide range of uncertainty or complex array of options
V. Reputational Risk: A critical decision entails a high level of organisational reputational risk

Decision	Criteria for Selection
i. Formation of Rapid Response Teams	☒ Characteristic 1<br>☐ Characteristic 2<br>☒ Characteristic 3<br>☒ Characteristic 4<br>☒ Characteristic 5
ii. Establishment of 3 strategic PCR labs	☒ Characteristic 1<br>☒ Characteristic 2<br>☒ Characteristic 3<br>☒ Characteristic 4<br>☒ Characteristic 5
iii. Launch of Incident Management System	☒ Characteristic 1<br>☐ Characteristic 2<br>☒ Characteristic 3<br>☒ Characteristic 4<br>☒ Characteristic 5
iv. Focus on case management through scale up of Oxygen	☒ Characteristic 1<br>☐ Characteristic 2<br>☒ Characteristic 3<br>☒ Characteristic 4<br>☒ Characteristic 5

For peer review only

Summary Decision Scorecard

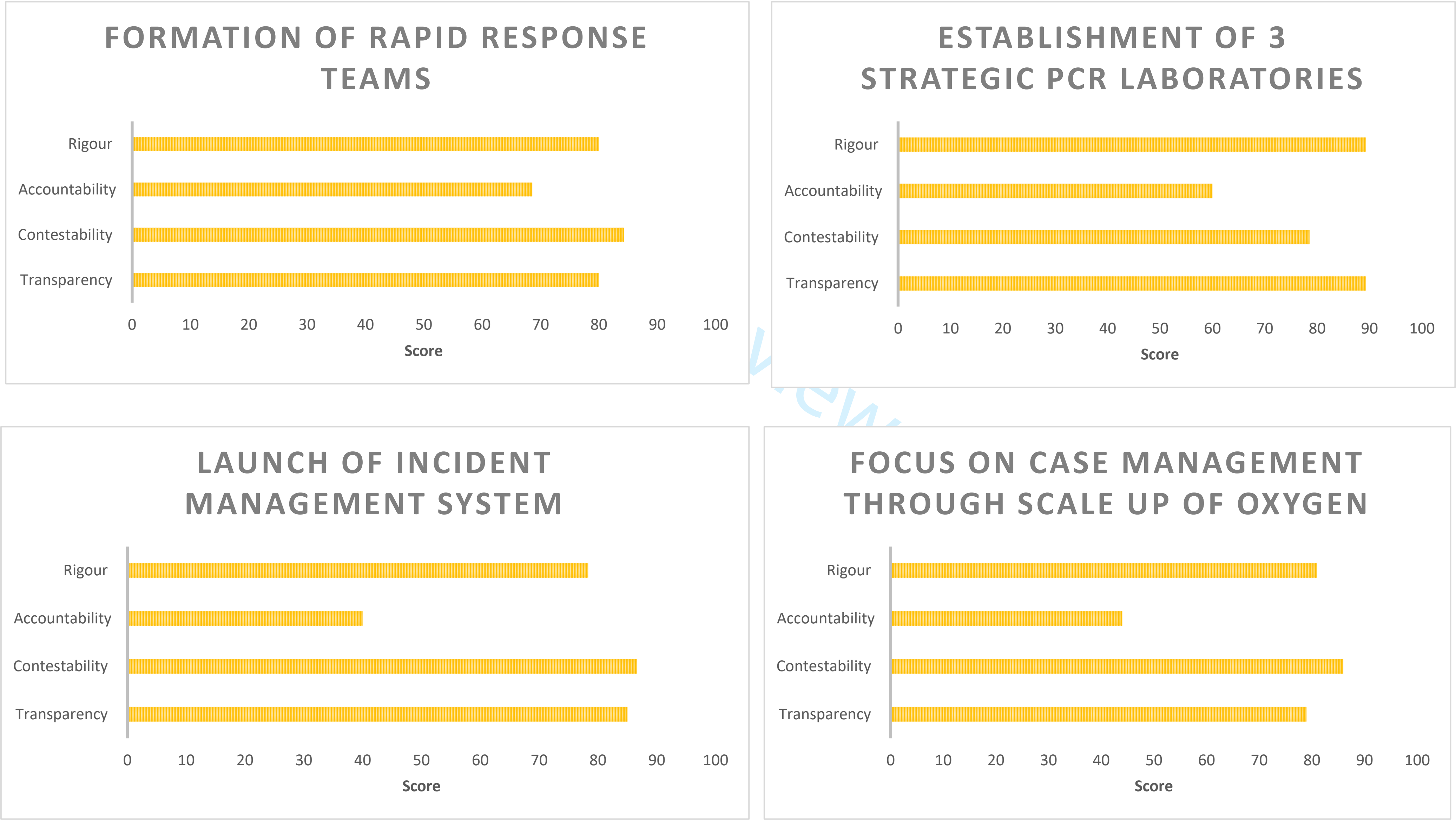

Detailed Scores & Interpretation

i. Formation of Rapid Response Teams

The Rapid Response Teams (RRT) and Community Health Workers (CHW) were recruited early in the COVID-19 response in order to strengthen and expand the surveillance capacity in high priority districts. A decision was undertaken to train and equip members of the RRTs and CHWs for case detection, notification, testing and contact tracing.

Inclusivity 86%	Decision criteria 74%	Process 77%	Public rationale 83%

Participants scored the transparency of this decision as high citing existing documentation such as response plan, IMST meeting minutes and proposal documents. They stated that there was strong evidence of inclusive decision-making as the decision included WHO staff from all programs as well as program managers. They also stated that some of the decision-makers were assigned to liaise/coordinate with the stakeholders including MoH and other partners and discuss the decision with them and publicise the rationale. They also noted following clear method for arriving at this decision including utilizing risk assessments. Participants referenced the usage of COVID-19 transmission classification for Somalia drawn from the interim guidelines by WHO for considerations for implementing and adjusting public health and social measures in the context of COVID-19 as providing clear criteria for this decision.

Devolution 74%	Revision 94%	Community engagement 69%	Explicit outcome 77%

Contestability was the highest scored dimension. Participants noted there were sufficient opportunities to revise the decision and cited the inter-action review of November 2020 as evidence. Additionally, the decision was discussed in various IMST meeting with document shared and commented, feedbacks incorporated into the final decision. The MoH was also said to be involved in the selection of RRTs and CHWs, implementation and modification of the structure and role and responsibilities. They also noted that revision of this decision resulted in expansion of the RRT mechanism to further district. Participants also noted that the IMST structure allowed for devolution in the decision-making in which decision-making was shared amongst senior decision-makers (country level IMST team members under the leadership of the WR) and junior decision makers (state level and district level staff)

In terms of accountability, this was scored lower as there was difference of scoring amongst participants as to the degree of engagement with the effected communities. Some participants stated that there was strong engagement with communities in implementing the RRT strategy but limited engagement in the decision to use this mechanism.

Feasibility 88%	Strengthen Health system 74%	Evidence based 77%

Participants noted this decision was taken with a high degree of rigour. In particular, they noted that strengthening healthcare system was among the objectives. Furthermore, they noted that the outcomes of this decision were explicitly articulated and include bridging the gap between health facilities and communities. They also noted that feasibility criteria were considered to be high due to staffing the RRT via the existing network of CHWs. Lastly they noted that targets were set and tracked through an interactive dashboard.

For peer review only

ii. Establishment of 3 strategic PCR labs

At the outset of the pandemic in Somalia, there did not exist national capacity to test samples for COVID-19. Responders relied in the first weeks, upon sending samples for testing abroad which were found to be time consuming and expensive. The lack of national labs to undertake this critical function was found to be a gap which required urgent attention. As a result, the decision was taken to establish three strategic PCR capable laboratories in Mogadishu, Garowe and Hargeisa.

Inclusivity 91%	Decision criteria 89%	Process 89%	Public rationale 89%

The transparency score is comprised of 4 criteria. In terms of inclusivity, participants scored this decision highly. terms of transparency. The decision was taken in conjunction with the MoH and UN partners. The rationale behind this decision was said to have been shared in internal coordination meetings as well as external coordination meetings with partners. A number of documents were cited as evidence including WHO Somalia Technical Program Update Quarterly, IMST meeting minutes, CERF Project Action Plan and WCO FCDO report.

Devolution 65%	Revision 91%	Community engagement 60%	Explicit outcome 91%

In terms of contestability, this decision was reviewed in the initial proposal development meeting with the MoH in which there was opportunity for revision. Additionally, participants stated that a review of the decision prompted further procurement of PCR machines and scaling up of PCR testing capacity to remaining states.

Participants expressed mixed scoring on the accountability dimension. Some stated that there was high accountability through interaction with state level MoH while others expressed that there was limited/no consultation with communities in these 3 cities regarding the establishment of PCR capacity in these labs. Some participants stated this criterion was inapplicable as the labs were pre-existing and upgrading did not require further engagement.

Feasibility 91%	Strengthen Health system 89%	Evidence based 89%

Participants scored this decision highly in terms of scientific rigour. They stated that the usage of PCR is global standard to detect COVID-19. Additionally, they stated the decision was built on evidence extracted from a previous 2019 influenza testing lab assessment which provided rationale and process for the decision to set up these labs for COVID-19 testing. Participants also cited the National Action Plan for Health Security as providing evidence for this decision. The decision was also found to have satisfied the strengthened the health system criteria as the establishment of these 3 labs allowed for testing beyond COVID-19.

For peer review only

iii. Launch of Incident Management System

As part of efforts to enhance the effectiveness of the Health Emergencies response and in line with the Emergency response framework, WHO Somalia launched the Incident Management Support Team to coordinate and guide the response to the COVID-19 pandemic within Somalia.

Inclusivity 90%	Decision criteria 83%	Process 80%	Public rationale 87%

Participants scored the inclusivity of this decision as high as they stated this decision was taken after a series of consultation with staff led by the WR. These deliberations were captured in meeting minutes and which detailed the process and rationale. The final decision was communicated by WR to all staff through an office memo. Participants also noted the engagement with the MoH in the formation of the IMST system as evidence of inclusivity.

Devolution 80%	Revision 93%	Community engagement 40%	Explicit outcome 77%

In terms of revision, participants noted that there was some opportunity which resulted in the change of incident managers but not in the structure itself. With regards to accountability, participants noted limited to no engagement with effected communities. However other participants stated that parallel state and district-level IMST did engage with the local community. While these lower level IMST did not feed into the formation of the national IMST, they maintain a certain level autonomy.

Feasibility 83%	Strengthen Health system 80%	Evidence based 73%

Participants cited a number of routine information products as evidence of decision outcomes and feasibility considerations. They cited COVID-19 Response plan and action trackers as evidence for the articulation of clear outcomes and evidence based targets. They also cited this response plan which articulated heath system strengthening as an explicit goal and an important pillar in the IMST approach. Participants also cited the WHO Global guidance on strategic response to C19 as providing evidence to support this decision.

iv. Focus on case management through scale up of Oxygen

Due to the presentation of symptoms and the aetiology of the disease as well as gaps in case management capacity, WHO Somalia undertook concerted effort to scale up oxygen therapy across the country in order to improve outcomes.

Inclusivity 76%	Decision criteria 76%	Process 76%	Public rationale 88%

The decision-making was scored as somewhat inclusive by participants. They cited the IMST meeting minutes in June 2020 as evidence as well as the COVID-19 response plan which detailed the discussion and decision criteria. They noted close deliberation with the case management focal persons within the MoH. Participants also noted that the rationale was communicated clearly through specific information products and web stories on WHO and other websites.

For peer review only

Devolution 84%	Revision 88%	Community engagement 44%	Explicit outcome 76%

Participants noted close discussion with federal member states on the modality of scale up as evidence of devolved decision-making. Participants mentioned that there were several opportunities to revise this decision in which the need for oxygen provision was reaffirmed but the modality was revised. They noted that the initial decision was for WHO to support procurement of oxygen locally. This changed to procurement of cylinders and ventilators and eventually to supporting the establishment of oxygen plants. Participants mentioned limited evidence of community engagement in the decision itself but significant engagement through health messaging after the fact. They cited dashboards as containing evidence of clear outcomes and targets that underlay this decision.

Feasibility 80%	Strengthen Health system 84%	Evidence based 84%

Participants noted that this decision supported strengthening of the health system by fortifying performance of isolation facilities and optimizing case management. They also cited later documents as providing evidence that underlay this decision such as the technical update on survival among hospitalized COVID-19 patients.

Recommendations

A number of recommendations were put forward by participants in order to strengthen the decision-making process within the organisation. They include the following:

- Improve documentation on decision-making deliberations as participants were aware of many discussions that were not reflected in official internal or external records.
- To further strengthen decision mechanisms by clearly outline who is involved, their responsibilities and the mechanisms by which they arrived at the decision.
- Strengthen engagement with affected communities in addition to government officials *during* the decision-making process rather than solely communicating finalised decisions. This will strengthen accountability.
- Consider sustainability as a potential criterion for good decision-making
- Repeat this decision-making exercise on a more routine basis by adopting it as a component of internal evaluation

For peer review only

Annex

Score Criteria

Dimension	Criteria	Description
Transparency	Inclusivity	The extent to which the process was inclusive, reflected in heterogeneity in rank and roles amongst decision makers involved.
	Use of explicit decision-making criteria	The extent to which the goals and objectives of the decision were clearly pre-specified. The absence of post-decision rationalization.
	Following clear process or method	The extent to which a priority setting process was in place, reflected in demonstrated use of priority setting frameworks, decision trees or other mechanism.
	Use of mechanism to publicise rationale	The extent to which clear documentation on the decision exists as well as the method used to communicate decisions.
Contestability	Opportunity for revision	The extent to which there existed scope to revise and overturn a decision including the debating of alternatives and description of how consensus was reached.
	Was the decision devolved?	The degree to which participants in closest proximity to the epidemic (e.g. subnational level) or local technical experts participate in the decision, including consideration of rank.
Accountability	Engagement with affected communities	The degree to which affected communities were involved in the response decision-making including at a minimum whether they were informed of the response activities and what effect this notification had on the communities.
Rigour	Explicit outcome	The extent to which intended outcomes of the decision were clearly articulated, including through setting of targets.
	Feasible outcome	The extent to which feasibility was considered in decision-making including debating of alternatives.
	Strengthens healthcare system	The extent to which the decision was in-line with wider strategy including the strengthening of the health system
	Evidence based	The extent to which the decision was based on strong public health rationale and robust scientific information.

For peer review only

Decision-making audit scorecard

CARE Somalia's COVID-19 response

Evaluation study

November 8th -Nov 22nd2021

Abdihamid Warsame

Faculty of Epidemiology and Population health

London School of Hygiene and Tropical Medicine

21 January 2022

For peer review only

For peer review only - http://bmjopen.bmj.com/site/about/guidelines.xhtml

Background

On the 30th of January 2020, the World Health Organization (WHO) declared the emergence of SARS-CoV-2 corona virus as a Public Health Emergency of International Concern (PHEIC) because of evident human-to-human transmission and renamed it COVID-19. On the 16th of March 2020, Somalia reported its 1st COVID- 19 confirmed case, a Somali national with travel history to China. As of the 20th of January 2022, Somalia had 24,261 confirmed cases of COVID-19 with 1335 deaths. The Federal Ministry of Health launched the National COVID-19 Preparedness & Response Plan on the 26th March 2020 with a budget of USD 57 million and estimated caseload of 6,000 confirmed cases.

Somalia's capacity to prevent, detect, and respond to any global health security threat scored six out of 100 on the 2016 Health Emergency Preparedness Index. Poor urban communities, internally displaced persons(IDPs), the elderly, households of minority clans, riverine communities, women and child headed households, and children are most at risk due to social exclusion and decreased accessibility to health services, sanitation and water.

The COVID-19 pandemic weakened Somalia's already fragile health system that has been negatively impacted by protracted conflict, cyclic drought, and locust infestation. The Somali health system has not been able to cope adequately with the demands of a COVID-19 response with poor and few testing facilities, low capacity of health care workers and poor funding. Social cultural practices, rumours, misconceptions, poor hygiene practices and communal caring for the sick in homes and communities, have additionally amplified the spread of the virus.

Methods

The evaluation occurred in Garowe, Somalia from 8- 18th of November 2021. A total of 8 participants from various units within CARE Somalia took place in this evaluation.

The evaluation was undertaken following the standard operating procedures (SOP) developed by the LSHTM research team (See annex) and with the appointment of a CARE focal point. The CARE focal point gathered the necessary background documentation as well as supported the formation of the decision-making committee through recruitment of evaluation participants from the organization. The focal point completed the first draft of the contextual analysis as well as consolidated input from the other participants. He was also instrumental in facilitating the group work sessions.

For peer review only - http://bmjopen.bmj.com/site/about/guidelines.xhtml

Critical Decisions

Participants were advised to select important decisions for critical evaluation based on the following criteria on what constitutes a critical decision.

Characteristics

A critical decision is one has one or more of the following characteristics:

I. Consequentiality-: A critical decision is consequential and shapes the response to a significant degree
II. Reversibility-: A critical decision can be difficult to overturn or reverse at least in the short term
III. Strategic: A critical decision entails a significant shift in terms of action taken, resources committed or precedent set
IV. Uncertainty: A critical decision entails a wide range of uncertainty or complex array of options
V. Reputational Risk: A critical decision entails a high level of organisational reputational risk

Decision	Criteria for Selection
i. Adaptation of nutrition programming to COVID-19	☐ Characteristic 1<br>☒ Characteristic 2<br>☒ Characteristic 3<br>☐ Characteristic 4<br>☐ Characteristic 5
ii. Closure of CARE offices and restriction of staff movement	☒ Characteristic 1<br>☐ Characteristic 2<br>☒ Characteristic 3<br>☐ Characteristic 4<br>☒ Characteristic 5
iii. Implementation of COVID-19 project in Sool and Sanaag	☒ Characteristic 1<br>☒ Characteristic 2<br>☒ Characteristic 3<br>☒ Characteristic 4<br>☐ Characteristic 5

For peer review only - http://bmjopen.bmj.com/site/about/guidelines.xhtml

Summary Decision Scorecard

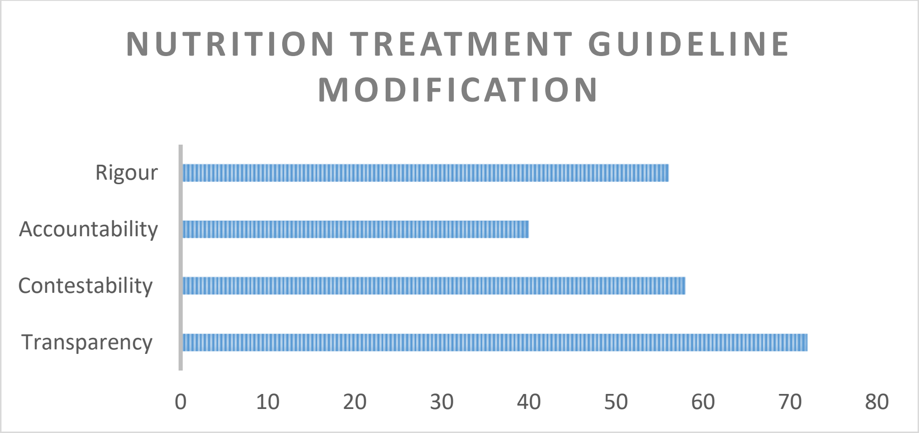

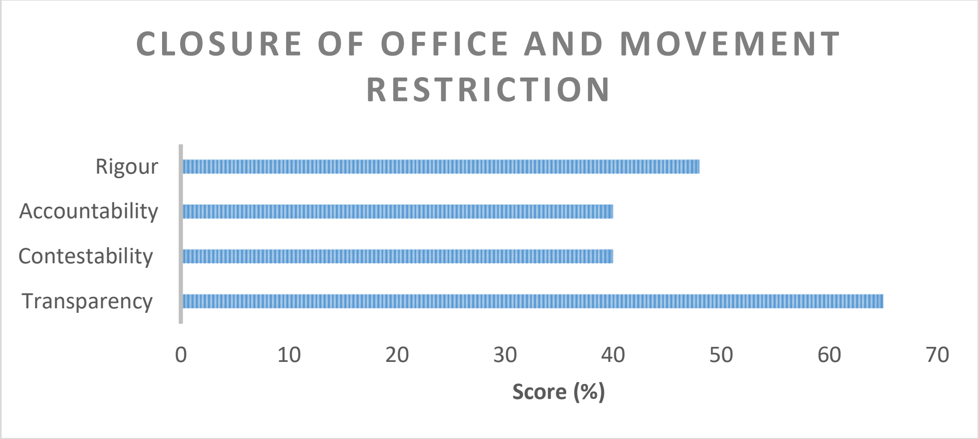

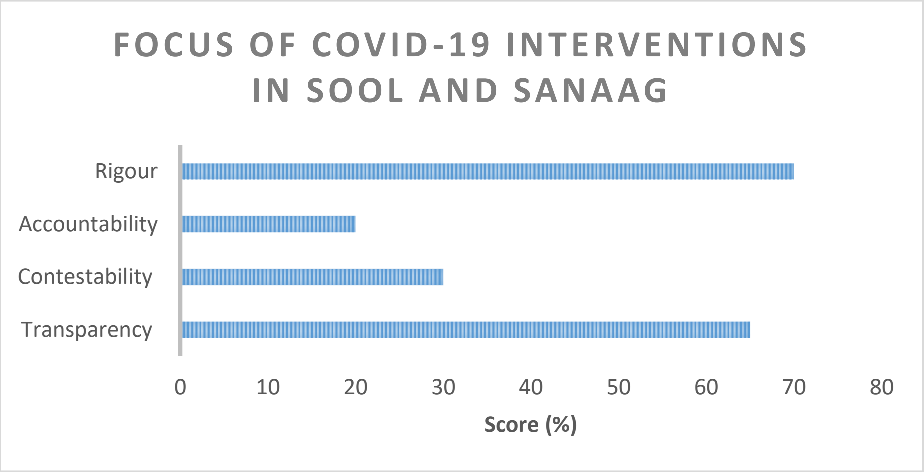

For peer review only - http://bmjopen.bmj.com/site/about/guidelines.xhtml

LONDON SCHOOL of HYGIENE &TROPICAL MEDICINE

Detailed Scores & Interpretation

1. Nutrition treatment guideline modification

Inclusivity 72%	Decision criteria 76%	Process 64%	Public rationale 76%

In terms of transparency, the decision to modify nutrition programme guidelines to be COVID-19 compatible was found to be fairly transparent. However, participants described a deficit in accountability as these modifications were reportedly made at the cluster level without the input of all partners. Additionally, participants reported that the affected communities were not consulted and could not feed into this decision-making process.

Devolution 44%	Revision 72%	Community engagement 40%	Explicit outcome 56%

Moreover, participants noted they did not have opportunity to contest these adaptations before they were adopted. They did however agree that there was a clear rationale to minimize transmission risk to the population. Nevertheless, participants did make clear the missed opportunity in engaging with the community to prepare them for these modifications.

Feasibility 55%	Strengthen Health system 60%	Evidence based 53%

Participants expressed concern that these adaptations would reduce patient follow-up, risk missing eligible program beneficiaries and could lower the effectiveness of the program.

2. Closure of Office and movement restriction

Inclusivity 60%	Decision criteria 76%	Process 64%	Public rationale 60%

The closure of CARE offices and restriction of staff movement was done early in the response. The decision was taken by the senior management and communicated via email to all staff. As a result, evaluation participants felt that this approach lessened the transparency as an opportunity was not provided to staff to feed into this decision. There was no clear documentation provided on the deliberations of this decision and the various options considered.

Participants did concede that it was possible that there were prioritization and decision-frameworks utilized but they were not disseminated to all staff.

Feasibility 60%	Strengthen Health system 40%	Evidence based 36%

Participants noted that in terms of supporting evidence, WHO and other international guidelines were possibly followed. No specific document was cited. However, the driving factor was to preserve business continuity and an abundance of caution as there were limited evacuation options for staff who felt ill.

Devolution 36%	Revision 44%	Community engagement 40%	Explicit outcome 60%

Participants mentioned that the decision to close offices was not devolved to sub-offices but was made at the country level. Additionally, there was limited community engagement with participants noting that as this could possibly affect program delivery, there should have been some level of community engagement.

For peer review only - http://bmjopen.bmj.com/site/about/guidelines.xhtml

3. Focus of COVID-19 Interventions in Sool and Sanaag

Inclusivity 40%	Decision criteria 80%	Process 80%	Public rationale 60%

CARE has largely been conducting its humanitarian and development programming in rural areas prior to the pandemic. When the pandemic occurred, fundraising and response resources were focused to areas which CARE had a strong field presence. These were in Sool and Sanaag regions in largely rural areas. However, the majority of COVID-19 cases were in urban centres and in higher density areas of the country. Thus there was a mismatch between where resources were directed and where they were most required. Participants explained that as a result of this prioritization of existing programme areas, there was inadequate strategic focus and limited contestability in the decision.

Devolution 40%	Revision 20%	Community engagement 20%	Explicit outcome 60%

Participants reported limited community engagement. However, there was ample opportunities to revise this decision according to some participants. There was also sufficient devolution with the design of the COVID-19 intervention done by both senior management as well as some technical staff based in the sub-offices.

Feasibility 80%	Strengthen Health system 80%	Evidence based 60%

Participants did not feel that this decision strengthened the health system as much as it could have if resources were more precisely directed where needed. They did cite usage of early available WHO guidelines as having a role in how the decision was undertaken. Participants rated the feasibility criteria as high based on the consideration by decision-makers to implement the COVID-19 interventions in existing program areas.

Conclusion

Overall very few primary or secondary documents were specifically cited by participants as evidence for the decision-making criteria. Participants were aware to varying degrees that some documents such as minutes and guidelines did influence decisions but were largely unable to cite them for this evaluation. They instead relied primarily on their recollection of events and discussions with fellow participants to piece together the events that transpired.

For peer review only - http://bmjopen.bmj.com/site/about/guidelines.xhtml

Recommendations

Decision-making

In order to improve the quality of decision-making in CARE's COVID-19 response and more broadly in its humanitarian responses, the following recommendations have been put forward:

1) Improve the inclusivity of key critical decision-making through expanding the membership of decision-makers to include junior staff and field staff.
2) Improve trust and buy-in of decisions through strengthening reporting of decision rationale and criteria.
3) Strengthen documentation of response activities to allow for more in-depth real-time or post response evaluation.
4) Consider more critically the relevance of scientific evidence and public health measures to the context rather than blanket adoption.
5) Allow for more contextually specific factors to shape decisions rather than blanket decisions

Evaluation Process

1) Allow for more time pre-evaluation in order for evaluation team to orient themselves with the methodology.
2) Translate evaluation tools and documentations into local language to allow for better understanding and involvement of evaluation participants
3) Allow for remote or asynchronous completion of evaluation tools by participants
4) Broaden the evaluation participants to include non-emergency staff and representatives of the community and local government
5) Consider practise scenario to allow for better uptake by evaluation team prior to commencing evaluation

For peer review only - http://bmjopen.bmj.com/site/about/guidelines.xhtml

Annex

Evaluation Schedule

Step	Action	Responsible	Date
1	Recruitment of evaluation team and signing of consent forms	All participants	November 8th
2	First Part A- Draft of Context Analysis	CARE Evaluation Focal Person	November 11
3	Review of Part A- Context Analysis	All Participants	November 13th
4	Set up Meeting	LSHTM & CARE focal person	November 14th
6	Completion of Part B- Decision Selection	All participants	November 16
7	Decision Ranking Exercise	All Participants as group	November 16
8	Completion of Part C- Scoring of Decisions	All Participants separately	November 17
8	Completion of Part D- Overall Scoring	LSHTM	November 25th
9	Key Informant Interview	All Participants	November 18- 21st

For peer review only - http://bmjopen.bmj.com/site/about/guidelines.xhtml

LONDON SCHOOL of HYGIENE &TROPICAL MEDICINE

Score Criteria

Dimension	Criteria	Description
Transparency	Inclusivity	The extent to which the process was inclusive, reflected in heterogeneity in rank and roles amongst decision makers involved.
	Use of explicit decision-making criteria	The extent to which the goals and objectives of the decision were clearly pre-specified. The absence of post-decision rationalization.
	Following clear process or method	The extent to which a priority setting process was in place, reflected in demonstrated use of priority setting frameworks, decision trees or other mechanism.
	Use of mechanism to publicise rationale	The extent to which clear documentation on the decision exists as well as the method used to communicate decisions.
Contestability	Opportunity for revision	The extent to which there existed scope to revise and overturn a decision including the debating of alternatives and description of how consensus was reached.
	Was the decision devolved?	The degree to which participants in closest proximity to the epidemic (e.g. subnational level) or local technical experts participate in the decision, including consideration of rank.
Accountability	Engagement with affected communities	The degree to which affected communities were involved in the response decision-making including at a minimum whether they were informed of the response activities and what effect this notification had on the communities.
Rigour	Explicit outcome	The extent to which intended outcomes of the decision were clearly articulated, including through setting of targets.
	Feasible outcome	The extent to which feasibility was considered in decision-making including debating of alternatives.
	Strengthens healthcare system	The extent to which the decision was in-line with wider strategy including the strengthening of the health system
	Evidence based	The extent to which the decision was based on strong public health rationale and robust scientific information.

For peer review only - http://bmjopen.bmj.com/site/about/guidelines.xhtml

**No.**
S1
S2
S3
S4
S5
S6
S7
S8
S9
S10
S11
S12
S13
S14
S15
S16
S17
S18
S19
S20
S21

For peer review only

Requirement
Title
Abstract
Problem Formulation
Purpose or research question
Qualitative approach and research paradigm
Researcher characteristics and reflexivity
Context
Sampling strategy
Ethical issues pertaining to human subjects
Data collection methods
Data collection instruments and technologies
Units of study
Data processing
Data analysis
Techniques to enhance trustworthiness
Synthesis and Interpretation
Links to empirical data
Integration with prior work, implication, transferability and contributions to the field
Limitations
Conflicts of Interest
Funding

For peer review only

Location in manuscript
Page 1
Page 2
page 2 lines
Page 3
page 9
page 8
Page 7
page 6
page 20
pages 6-8
page 4 and supplementary material
pages 7-8
page 8
page 9
page 4 and supplementary material (publication of audit tools and reports)
pages 9-16
pages 11-16
pages 4, 16-18
page 18
Page 20
Page 20

For peer review only
